# An ancestral NB-LRR with duplicated 3′UTRs confers stripe rust resistance in wheat and barley

**DOI:** 10.1038/s41467-019-11872-9

**Published:** 2019-09-06

**Authors:** Chaozhong Zhang, Lin Huang, Huifei Zhang, Qunqun Hao, Bo Lyu, Meinan Wang, Lynn Epstein, Miao Liu, Chunlan Kou, Juan Qi, Fengjuan Chen, Mengkai Li, Ge Gao, Fei Ni, Lianquan Zhang, Ming Hao, Jirui Wang, Xianming Chen, Ming-Cheng Luo, Youliang Zheng, Jiajie Wu, Dengcai Liu, Daolin Fu

**Affiliations:** 1 State Key Laboratory of Crop Biology, Shandong Agricultural University, 271018 Tai’an, Shandong China; 2 Department of Plant Sciences, University of Idaho, Moscow, ID 83844 USA; 3 Triticeae Research Institute, Sichuan Agricultural University, 611130 Chengdu, Sichuan China; 4 Department of Plant Pathology, Washington State University, Pullman, WA 99164 USA; 5 Department of Plant Pathology, University of California, Davis, CA 95616 USA; 6 Wheat Health, Genetics, and Quality Research Unit, USDA-ARS, Pullman, WA 99164 USA; 7 Department of Plant Sciences, University of California, Davis, CA 95616 USA; 8 State Key Laboratory of Crop Gene Exploration and Utilization in Southwest China, Sichuan Agricultural University, 611130 Chengdu, Sichuan China

**Keywords:** Agricultural genetics, Genetic linkage study, Biotic

## Abstract

Wheat stripe rust, caused by *Puccinia striiformis* f. sp. *tritici* (*Pst*), is a global threat to wheat production. *Aegilops tauschii*, one of the wheat progenitors, carries the *YrAS2388* locus for resistance to *Pst* on chromosome 4DS. We reveal that *YrAS2388* encodes a typical nucleotide oligomerization domain-like receptor (NLR). The *Pst*-resistant allele *YrAS2388R* has duplicated 3’ untranslated regions and is characterized by alternative splicing in the nucleotide-binding domain. Mutation of the *YrAS2388R* allele disrupts its resistance to *Pst* in synthetic hexaploid wheat; transgenic plants with *YrAS2388R* show resistance to eleven *Pst* races in common wheat and one race of *P*. *striiformis* f. sp. *hordei* in barley. The *YrAS2388R* allele occurs only in *Ae. tauschii* and the *Ae. tauschii*-derived synthetic wheat; it is absent in 100% (*n* = 461) of common wheat lines tested. The cloning of *YrAS2388R* will facilitate breeding for stripe rust resistance in wheat and other Triticeae species.

## Abstract (web summary)

Stripe rust is a serious threat to wheat production. Here, the authors reveal that the resistance gene, only present in the wheat progenitor *Aegilops tauschii* and its derived synthetic wheat, encodes a nucleotide oligomerization domain-like receptor and confers resistance in common wheat and barley.

## Introduction

Wheat (*Triticum* spp.) is the largest acreage crop in the world. With an approximate 220 million hectares and 760 million tons in 2018, wheat was ranked second in global production after maize^1^. As a staple food crop, wheat provides about 20% of global calories for human consumption^2^. Because the world population is projected to increase by nearly two billion people within the next three decades^3^, the increasing human population worldwide will place an even greater demand for wheat production globally.

Wheat stripe rust (or yellow rust; abbreviated as Yr), caused by *Puccinia striiformis* f. sp. *tritici* (*Pst*), is a serious fungal disease that poses a huge threat to wheat production in regions with cool and moist weather conditions^4^, including major wheat-producing countries, such as Australia, Canada, China, France, India, the United States, and many others^5,6^. Planting wheat cultivars with adequate levels of resistance is the most practical and sustainable method to control stripe rust. Host resistance of wheat against *Pst* is normally classified as either all-stage resistance (ASR) or adult-plant resistance (APR). Whereas ASR is effective starting at the seedling stage through the late stages of plant growth, APR is mainly effective at the late stages of plant growth^7^. In wheat, ASR confers high levels of resistance to specific *Pst* races, but the underlying genes, such as *Yr9*^8^ and *Yr17*^9^, are often circumvented by the emergence of new virulent races. In contrast, APR typically provides a partial level of resistance, but is more durable and is effective against all or a wider spectrum of *Pst* races than ASR. High-temperature adult-plant (HTAP) resistance is a major type of APR; HTAP typically provides durable and non-race-specific resistance to *Pst*^10^. Incorporating multiple ASR and HTAP genes appears to be an excellent strategy for maintaining sustainable resistance to wheat stripe rust^10^.

Over 80 wheat stripe rust resistance (*R*) genes (*Yr1–Yr81*) have been permanently named^11^. Of the seven genes cloned so far, *Yr5*, *Yr7* and *YrSP*, a gene cluster, encodes nucleotide-binding (NB) and leucine-rich repeat (LRR) proteins^12^; *Yr15* has two kinase-like domains^13^; *Yr36* has a kinase domain and a lipid binding domain^14^; *Lr34/Yr18* encodes a putative ABC transporter^15^; and *Lr67/Yr46* encodes a predicted hexose transporter^16^. While the *Yr5/Yr7/YrSP* cluster and *Yr15* confer ASR resistance to wheat stripe rust, *Yr18*, *Yr36*, and *Yr46* confer APR or HTAP resistance. Of the three cloned APR genes, only the *Yr18* gene has been widely used in wheat cultivars^17^; however, *Yr18* alone does not confer adequate resistance under high disease pressures. *Yr7* and *YrSP* confer high levels of resistance, but *Pst* races virulent to *Yr7* are common globally and those virulent to *YrSP* occur in some countries^18^. *Yr5* and *Yr15* confer high levels of resistance to a wide range of *Pst* races^12,19^, but the increasing adoption of them in wheat cultivars may cause the emergence of virulent races. Characterization of additional *R* genes is essential in order to assemble effective resistance to constantly changing populations of *Pst*.

*Aegilops tauschii* Coss. (2*n* = 2 × = 14, DD) is the D genome progenitor of common wheat^20,21^. The diverse *Ae. tauschii* D genome offers a valuable gene pool for stripe rust resistance^20,22^. To date, several stripe rust resistance genes have been mapped in *Ae. tauschii*, including *YrAS2388*^23^ and *Yr28*^24^ on 4DS^22,25^, and *YrY201*^26^ on 7DL. Synthetic hexaploid wheat (SHW) lines, which contain a diversity of *Ae. tauschii* accessions^27^, are potential breeding stocks. However, many biotic and abiotic resistance genes are suppressed in the hexaploid background^28^. To prevent a linkage drag of undesirable traits and resistance suppression, it is best to identify *R* genes and use them precisely in gene pyramids. In this study, we have cloned the stripe rust resistance gene *YrAS2388* from *Ae. tauschii*. Additionally, we have demonstrated that this gene can express effectively in hexaploid wheat and barley. Deployment of *YrAS2388R* in wheat cultivars together with other effective genes should sustainably protect wheat production from the devastating disease wheat stripe rust.

## Results

### *YrAS2388R* confers resistance to wheat stripe rust

**Fig. 1:**
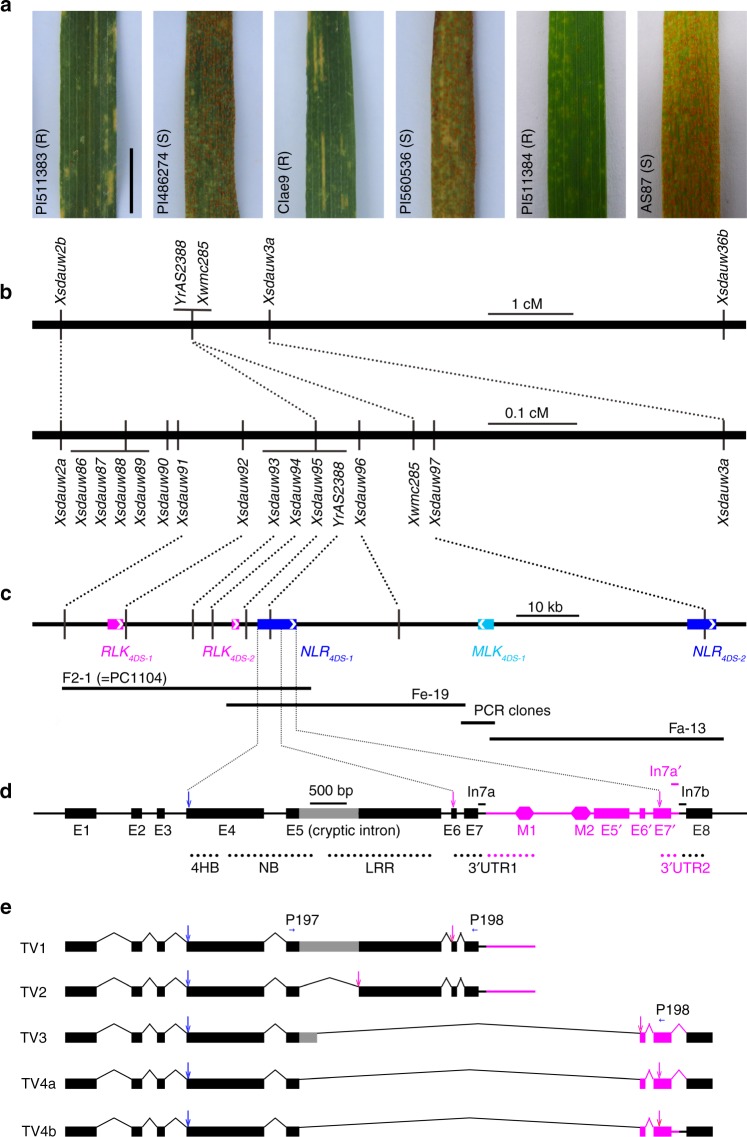
Map-based cloning of the *YrAS2388* gene. **a** Adult plant responses (R = resistant; S = susceptible) of parental lines to natural spores in the field. Scale bar = 1 cm. **b** Genetic maps are based on popC-1 (upper) and popA-2 (lower). **c** Physical maps are based on three fosmid clones: F2-1 (= PC1104), Fe-19 and Fa-13, which contain five genes (colored rectangles; arrows pointing to 3′ ends) that encode for two 4-helix bundle-nucleotide binding-leucine rich repeat (NLRs), a malectin-like kinase (MLK), and two receptor-like kinases (RLKs). A 3.9-kb physical gap between Fe-19 and Fa-13 was closed by sequencing PCR clones. **d** Genomic structure of *NLR*_*4DS-1*_ in PI511383. The conserved domains and the duplicated 3′UTRs are labelled; their approximate genomic locations are highlighted with dotted lines. The 3′UTR duplication was caused by a 2668-bp insertion (magenta region), which has three regions (with a prime symbol) similar to exons 5, 6, and 7. Introns 7a and 7b, and exon 8 are the original components of the ancestral 3′UTR, but the 2668-bp insertion disrupted the ancestral 3′UTR and then formed two 3′UTRs, each containing both ancestral (black dots) and inserted (magenta dots) segments. The cryptic intron in exon 5 is highlighted by a gray box. Introns 7a, 7a′, and 7b have a size bar below their names. **e** Transcript variants of *NLR*_*4DS-1*_ in accession PI511383. Cloning and sequencing of the *NLR*_*4DS-1*_ cDNA clones identified five transcript variants, designated TV1 to TV4b, of *NLR*_*4DS-1*_ in accession PI511383. Grey boxes indicate portions of the retained intron in mature messenger RNA. Rectangles and straight lines indicate regions present in mRNA; the caret-shaped lines represent regions that are absent in mRNA. Part of the cryptic intron in exon 5 is retained in TV3. TV4a and TV4b encode an identical protein, called TV4. P197 and P198 are primers that detect all five splicing variants in one PCR. Abbreviations include exon (E), four-helical bundle (4HB), intron (In), leucine-rich repeat (LRR), two miniature inverted-repeat transposable elements (M1 and M2), nucleotide-binding (NB), and start (downwards arrows in blue) and stop (downwards arrows in magenta) codons. Source data of Fig. 1a are provided as a Source Data file

*Aegilops tauschii* CIae9, PI511383 and PI511384 (all from the subspecies (subsp.) *strangulata*) possess *YrAS2388R*^22^. At the two-leaf seedling (juvenile) stage, CIae9, PI511383, and/or PI511384 were resistant with infection types (IT) between 1 and 5 to nine *Pst* races (PSTv-3, PSTv-4, PSTv-11, PSTv-18, PSTv-37, PSTv-41, PSTv-51, PSTv-52, and PSTv-172), under low temperature (LT) and/or high temperature (HT) regimes (Table 1). These races are virulent on a wide range of wheat germplasm (Supplementary Data 1). CIae9, PI511383 and PI511384 have shown *Pst* resistance (IT scores = 1–3; Fig. 1a) under natural infections in the Sichuan basin in China since 1995. In contrast, *Ae. tauschii* AS87, PI486274 and PI560536 (all subsp. *tauschii* accessions) do not have the *YrAS2388R* gene^22^, and were always susceptible under either natural infections or controlled inoculation (IT scores = 7–9; Fig. 1a, Table 1).

**Table 1:** Seedling responses of selected lines to *Puccinia striiformis* f. sp. *tritici*

Materials^a^	Genomes	*YrAS2388R* ^b^	Infection types^c^				TR^d^
			PSTv-3	PSTv-11	PSTv-41	PSTv-172	HT/LT1
Clae9	DD	+^b^	2	2	1	2	HT
PI511383	DD	+	2	2	2	2	HT
AvSYr28NIL	AABBDD	+	1	1	*6*	2	HT
AvS	AABBDD	−^b^	8	8	8	8	HT
Clae9	DD	+	2	2	2	2	LT1
PI511383	DD	+	2	1	2	2	LT1
AvSYr28NIL	AABBDD	+	2	1	*8*	2	LT1
AvS	AABBDD	−	9	9	9	9	LT1
			PSTv-4	PSTv-18	PSTv-51	Field^e^	LT2
PI511383	DD	+	1	3	1	1	LT2
PI511384	DD	+	2	5	2	1	LT2
PI486274	DD	−	8	9	8	8	LT2
AS87	DD	−	8	8	8	8	LT2
SW3	AABBDD	+	*8*	*8*	*7*	*8*	LT2
SW58	AABBDD	−	8	8	5	8	LT2

^a^PI511384 = AS2388; SW3 = Langdon/CIae9^32^; SW58 = Langdon/AL8/78^32^; AvS = Avocet Susceptible^18^; AvSYr28NIL = Avocet + *Yr28*

^b^A plus sign = positive for the *YrAS2388R* (or *Yr28*) locus; a minus sign = negative for the *YrAS2388R* (or *Yr28*) locus

^c^Responses from 0 (immune) to 9 (massive sporulation) are according to McNeal’s scale^59^. Unexpected *Pst* responses are highlighted by an italicized font

^d^TR, temperature regimes: HT = high diurnal temperature cycle of 12°C/30 °C; LT (LT1 and LT2) = low diurnal temperature cycle of 4 °C/20 °C

^e^UI seedling test with field spores (from Parker Farm) under a low temperature regime in 2018. Field spores likely included PSTv-37 and/or PSTv-52

Previously, we hypothesized that *YrAS2388* and *Yr28* are the same gene^22^. AvSYr28NIL and AvS are near-isogenic lines (NILs) for the *Yr28* gene. During a *Pst* test with PSTv-3, PSTv-11, PSTv-41, and PSTv-172, AvS was susceptible to all races under both LT and HT, but AvSYr28NIL was highly resistant to PSTv-3, PSTv-11, and PSTv-172 (Table 1). We additionally tested two synthetic hexaploid wheat (SHW) lines, SW3 and SW58, that were derived from the durum wheat Langdon but have different D-genome donors: the *Pst*-resistant CIae9 and the *Pst*-susceptible AL8/78, respectively. Despite a dominant *YrAS2388R* gene in CIae9, SW3 was highly susceptible to PSTv-4, PSTv-18, PSTv-37 and PSTv-52 (Table 1), which was comparable to SW58 under LT. Thus, *YrAS2388R* can be suppressed when it is introgressed into certain hexaploid wheat genotypes.

### *YrAS2388R* was delimited to a 50-kb region in PI511383

CIae9, PI511383, PI511384 and eleven other accessions were found to have the *YrAS2388R* gene or allelic genes on 4DS^22^. We previously developed three F_2_ populations: popA (PI511383/PI486274), popB (CIae9/PI560536) and popC (PI511384/AS87). The *YrAS2388R*-based *Pst* responses are inherited as a Mendelian trait in all three F_2_ populations^22^. Here, among 1910 F_2_ plants of popC-2 (PI511384/AS87), 1,432 were resistant and 478 were susceptible in Wenjiang, Sichuan, China, which fits single dominant gene inheritance (Chi-Square goodness of fit test, *χ*^2^_3:1_ = 0.001, *P* = 0.98).

Using the wheat 10k iSelect array^29^, we genotyped CIae9, PI486274, PI511383, PI560536, and 17 *Pst*-susceptible F_2_ plants (10 from popA and 7 from popB) for bulked segregant analysis (BSA) of the *Pst*-susceptible allele of *YrAS2388* (*YrAS2388S*). Among 3276 applicable single nucleotide polymorphisms (SNPs), we selected 20 SNPs that were mostly associated with a *Pst*-susceptible phenotype; eight of them, including AT4D3406, AT4D3410, AT4D3411, AT4D3412, AT4D3413, AT4D3417, AT4D3418, and AT4D3419 (Supplementary Table 1), were in the 4DS distal region^29^. Based on specific genotypes per marker per plant, *YrAS2388S* was mapped distal to the AT4D3406 region (Supplementary Table 1).

The AT4D3406 region (Supplementary Fig. 1a) was initially targeted to map the *YrAS2388* gene in popA and popC. Using the F_2_ and F_3_ data, we mapped *YrAS2388* to the same region in popA-1 and popC-1 (Supplementary Fig. 1b, c). In popC-1, *YrAS2388* is between *Xsdauw2b* (= AT4D3403) and *Xsdauw3a* (= AT4D3405) (Supplementary Table 2), an approximate 2.4-cM interval (Supplementary Fig. 1c). To assure that we defined the correct region, we targeted a large interval, *Xsdauw2a* (= AT4D3403)*-Xsdauw36a* (= AT4D3410), for screening recombinants in popA-2. Additional markers were designed from the linkage map^29^ and genome sequence^30^ of *Ae. tauschii*. First, we retrieved the AT4D3403, AT4D3404 and AT4D3405 corresponding genomic sequence^29^, prioritized the low-copy number regions, created nine PCR markers (*Xsdauw86* to *Xsdauw91*, *Xsdauw93*, *Xsdauw95* and *Xsdauw97*) among six parental lines, and placed *YrAS2388* between *Xsdauw91* and *Xsdauw97* (Supplementary Fig. 1d). Second, we constructed a fosmid genomic library from the *Pst*-resistant genotype PI511383. *Xsdauw92*, *Xsdauw94* and *Xsdauw96* were then developed using the fosmid clones of the *YrAS2388* region. After analyzing 4205 popA F_3_ plants, which were from 11 F_2_ plants heterozygous in the *Xsdauw2a-Xsdauw36a* region, we precisely mapped *YrAS2388* between *Xsdauw92* and *Xsdauw96*, about a 0.13-cM interval, and added to the *YrAS2388* interval with three completely linked markers (*Xsdauw93*-*Xsdauw95*) (Fig. 1b, Supplementary Fig. 1d, Supplementary Table 2).

The fosmid genomic library of PI511383 has approximately one million clones and represents an eight-fold coverage of the *Ae. tauschii* genome (≈ 4.3 Gb^30^). Twenty fosmid clones were identified in the *YrAS2388* region. In the physical map (Fig. 1c, Supplementary Fig. 2), *Xsdauw91* and *Xsdauw96*, which delimited the *YrAS2388* gene, were anchored to the two overlapping fosmids, F2-1 and Fe-19. After sequencing F2-1 and Fe-19, we primarily analyzed the *Xsdauw91-Xsdauw96* region (ca. 50 kb). RNA sequencing (RNA-seq) in PI511383 revealed three active genes in the *Xsdauw91-Xsdauw96* interval, including two receptor-like kinase genes^31^ (*RLK*_*4DS-1*_ and *RLK*_*4DS-2*_), and a classic *R* gene, *NLR*_*4DS-1*_. Both *RLK*_*4DS-1*_ and *RLK*_*4DS-2*_ are wall-associated receptor kinases. *RLK*_*4DS-1*_ has an N-terminal galacturonan-binding and a C-terminal serine/threonine kinase (STK) domain, whereas *RLK*_*4DS-2*_ has only the STK domain. *NLR*_*4DS-1*_ has a classic four-helix bundle (4HB) that was previously classified as a coiled-coil, a NB domain and a LRR domain with 12 or more leucine-rich repeats. All three genes are highly conserved among the *Ae. tauschii* accessions tested and the collinearity of the *YrAS2388* region is conserved between *Ae. tauschii* and common wheat (Supplementary Fig. 2). Transcription of *RLK*_*4DS-1*_, *RLK*_*4DS-2*_ and *NLR*_*4DS-1*_ was confirmed by reverse-transcription PCR (RT-PCR) (Supplementary Fig. 3a, b). Genome sequence analysis, RNA-seq and RT-PCR further revealed that the *NLR*_*4DS-1*_ gene in PI511383 contains a 2668-bp insertion, which resulted in duplicated 3′ untranslated regions (3′UTR1 and 3′UTR2) and five transcript variants (Fig. 1d, e). However, the *NLR*_*4DS-1*_ gene in AL8/78 lacks the 2668-bp insertion and has only one transcript product.

The closest distal marker, *Xsdauw92*, placed *RLK*_*4DS-1*_ outside of the *YrAS2388* interval. Consequently, *NLR*_*4DS-1*_ and *RLK*_*4DS-2*_ became the most likely candidates for *YrAS2388*. Both genes were expressed in the *Pst*-resistant parents (CIae9, PI511383 and PI511384) and in two *Pst*-susceptible genotypes (AL8/78 and AS87), but were inactive in two *Pst*-susceptible parents (PI486274 and PI560536) (Supplementary Fig. 3a, b). In a comparison of *NLR*_*4DS-1*_ in the *Pst*-resistant (CIae9, PI511383 and PI511384) and the *Pst*-susceptible (AS87 and AL8/78) genotypes, the cDNA and protein sequences are only 94% and 91% identical, respectively (Supplementary Data 2). The *Pst*-susceptible genotypes (AL8/78 and AS87) had a premature stop codon in *RLK*_*4DS*-*2*_, which was absent in the *Pst*-resistant parents (CIae9, PI511383 and PI511384). Thus, both *NLR*_*4DS-1*_ and *RLK*_*4DS-2*_ remained as candidates for the *YrAS2388* gene.

### Haplotype markers indicated that *NLR*_*4DS-1*_ is *YrAS2388*

To help identify the correct gene, we genotyped 159 *Ae. tauschii* accessions using five markers for *NLR*_*4DS-1*_ (HTM3a to HTM3e, or collectively called HTM3S), one for *RLK*_*4DS-1*_ (HTM1a) and one for *RLK*_*4DS-2*_ (HTM2a) (Supplementary Tables 2 and 3, Supplementary Data 3). The *R*-type allele (e.g. “A” in PI511383) of *NLR*_*4DS-1*_ was completely associated with *Pst* resistance in resistant haplotypes R1 to R3 (Supplementary Data 3). All non-A scores of the *NLR*_*4DS-1*_ markers were associated with *Pst* susceptibility. The coding region (ATG to 3′UTR2; Fig. 1d) of *NLR*_*4DS-1*_ is identical amongst eight *Pst*-resistant *Ae. tauschii* accessions, including AS2386, AS2387, AS2399, AS2402, CIae9, PI349037, PI511383, and PI511384. In contrast, in *RLK*_*4DS-1*_ and *RLK*_*4DS-2*_, the *R*-type allele (e.g. “A” in PI511383) was present in the *Pst*-susceptible genotypes (S1-S3 and S5), indicating that both genes do not confer *Pst* resistance. Similarly, the absence of *RLK*_*4DS-1*_ and/or *RLK*_*4DS-2*_ in the R2 and R3 haplotypes suggested that neither gene is essential for *Pst* resistance. Thus, *NLR*_*4DS-1*_ is the only candidate for *YrAS2388R*.

### *Pst*-susceptible SHW mutants have more mutations in *NLR*_*4DS-1*_

**Fig. 2:**
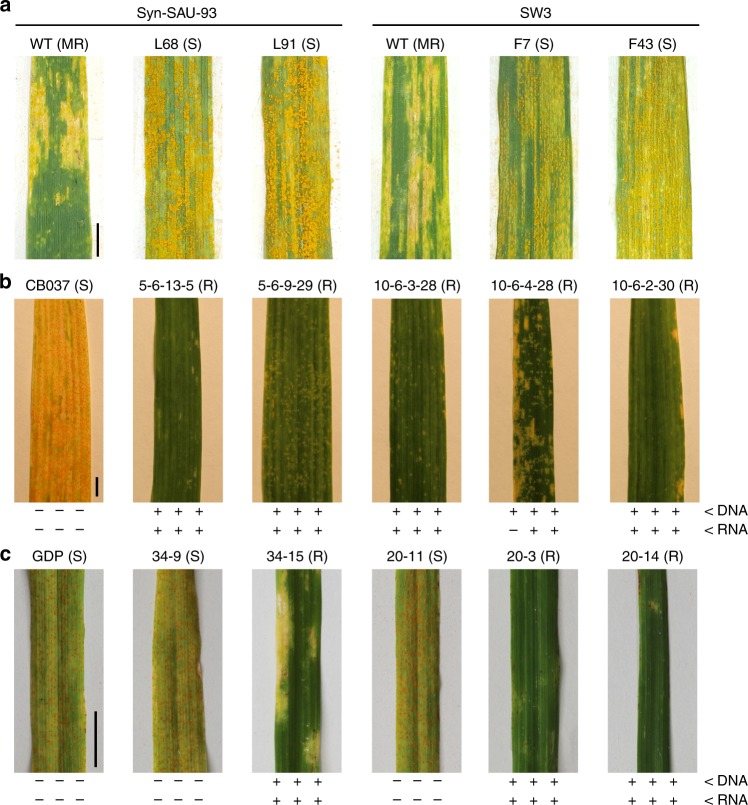
The *YrAS2388* locus confers stripe rust resistance in wheat and barley. **a** Syn-SAU-93 and SW3 are two synthetic hexaploid wheat (SHW) lines that express the *YrAS2388R* gene. WT is the resistant wild-type control with necrotic lesions. L68 (G117D), L91 (V267I), F7 (S394N) and F43 (V482I) are susceptible mutants with sporulating *Pst*. Plant responses (MR = moderate resistance; R = resistant; S = susceptible) to *Pst* are indicated in parentheses. **b** The susceptible hexaploid wheat CB037 was transformed with the intact PC1104 (= F2-1). Transgenic T_3_ wheat (all from the No. 5 and 10 T_2_ subfamilies) was challenged with PSTv-239 at the adult plant stage. Under each picture, PCR results as positive (plus signs) or negative (minus signs) for DNA amplification (upper) and RNA expression (lower) of the three target genes: *RLK*_*4DS-1*_ (left), *RLK*_*4DS-2*_ (middle) and *NLR*_*4DS-1*_ (right). RT-PCR is illustrated in Supplementary Fig. 4. **c** The susceptible barley Golden Promise (GDP) was transformed with the intact PC1104 ( = F2-1). Transgenic T_1_ barley seedlings were inoculated with race PSH-72 of *Puccinia striiformis* f. sp. *hordei* (*Psh*). Scale bar = 1 cm. Source data are provided as a Source Data file

Synthetic hexaploid wheat (SHW) SW3^32^ and Syn-SAU-93^33^ acquire the *YrAS2388R* gene from their D-genome donor; both SW3 and Syn-SAU-93 displayed moderate *Pst* resistance (IT scores = 3–5; Fig. 2a) in Sichuan, China. Using ethyl methanesulfonate (EMS), we generated 1132 M_2_ families of SW3 and 613 M_2_ families of Syn-SAU-93. Under field conditions, we identified 103 *Pst*-susceptible plants (IT scores = 7–9; Fig. 2a, Supplementary Data 4). For the *NLR*_*4DS-1*_, *RLK*_*4DS-1*_, and *RLK*_*4DS-2*_ genes, 51 *Pst*-susceptible mutants (49.5%) had a deletion in the *NLR*_*4DS-1*_ gene, of which 50 deletion events extended into *RLK*_*4DS-2*_ but only 11 deletion events extended further to *RLK*_*4DS-1*_ (Supplementary Data 4). However, no deletion only occurred in *RLK*_*4DS-1*_ and/or *RLK*_*4DS-2*_. Among the remaining 52 non-deletion mutants, 18 *Pst*-susceptible mutants had at least one base change in the *NLR*_*4DS-1*_ gene, and 16 of those mutations (89%) either caused an amino acid change or formed a premature stop codon (Supplementary Data 4). Seven amino acid variations were identified in the *NLR*_*4DS-1*_ gene, including Gly117Asp, Val267Ile, Ser394Asn, Leu421Phe, Thr456Ile, Val482Ile, and Gln557Stop(*) (Supplementary Data 4). However, all 52 non-deletion mutants had no mutation in the coding region of *RLK*_*4DS-2*_. Thus, *NLR*_*4DS-1*_ confers resistance to *Pst* in SW3 and Syn-SAU-93 and likely represents the *YrAS2388R* gene.

### *NLR*_*4DS-1*_ confers stripe rust resistance in wheat and barley

We transformed wheat and barley with the fosmid PC1104 ( = F2-1; Supplementary Table 4), which has a 40-kb genomic fragment with *NLR*_*4DS-1*_, *RLK*_*4DS-1*_ and *RLK*_*4DS-2*_. The spring wheat CB037^34^ was selected as the primary wheat recipient genotype because it is highly susceptible (IT scores = 7–9) to 11 *Pst* races, including PSTv-4, PSTv-14, PSTv-37, PSTv-39, PSTv-40, PSTv-47, PSTv-143, PSTv-221, PSTv-239, PSTv-306, and PSTv-353 (Supplementary Data 1). After bombarding 1,590 wheat immature embryos, we obtained 24 putative transgenic plants. *NLR*_*4DS-1*_, *RLK*_*4DS-1*_ and *RLK*_*4DS-2*_ were detected in four T_0_ plants (No. 4, 5, 10, and 22), but only two plants (No. 5 and 10) were positive for the *NLR*_*4DS-1*_ cDNA (Table 2, Supplementary Fig. 4). For No. 5 and 10 transgenic plants, we selected 13 subfamilies that were homozygous resistant to PSTv-40 in the T_2_ generation, and tested the T_3_ plants against nine *Pst* races (PSTv-37, PSTv-39, PSTv-47, PSTv-143, PSTv-221, PSTv-239, PSTv-306, PSTv-352, and PSTv-353) and *Pst* spores from the field (Supplementary Data 1). The T_3_ plants were resistant (IT scores = 0–4) to all *Pst* races at the seedling and adult-plant stages (Fig. 2b, Supplementary Fig. 5, Supplementary Data 1).

**Table 2:** Transgene expression and plant responses to *Puccinia striiformis*

Groups^a^	Constructs (treatment) ^b^	Events^c^	*RLK*_*4DS-1*_	*RLK*_*4DS-2*_	*NLR*_*4DS-1*_^d^	Responses *to Pst* (or *Psh*)^e^
G1	PC1104 (I)	1+3	+^d^	+	+	Resistant
G2	PC1104 (X1)	1	+	+	−	Susceptible
G3	PC1104 (I)	1	−^d^	+	+	Resistant
G4	PC1104 (X1, XK1)	2	+	−	−	Susceptible
G5	PC1104 (B1, N1, XK1)	5	−	+	−	Susceptible
G6	PC1104 (B1, N1, X1)	7+2	−	−	−	Susceptible
G7	PC1101 (*Ubi::NLR*_*4DS-1 TV1*_) ^f^	9+4	−	−	+	Susceptible
G8	PC1102 (*Ubi::NLR*_*4DS-1 TV2*_) ^f^	10+13	−	−	+	Susceptible

^a^Groups G1-G6 are based on genomic DNA, and Groups G7-G8 are based on cDNA

^b^PC1104 was either intact (I) or linearized with *Bsr*GI (B1), *Not*I (N1), *Xba*I (X1) and *Xba*I plus *Kpn*I (XK1). Intact or linearized plasmid per enzyme was introduced into recipient genotypes separately

^c^Per cell, the first number indicates the number of wheat transformants; when there are two numbers, the second number indicates the number of barley transformants

^d^A plus sign = positive for full-length gene expression by PCR; a minus sign = negative for gene expression. RT-PCR is illustrated in Supplementary Fig. 4

^e^PSTv-40 and PSH-72 were used to test the transgenic wheat and barley, respectively.

^f^The *NLR*_*4DS-1*_ cDNAs were under the maize *Ubi* promoter^68^; no digestion was applied to them

Barley cultivar Golden Promise is susceptible to race PSH-72 of the barley stripe rust pathogen, *P. striiformis* f. sp. *hordei* (*Psh*). After bombarding 2,200 barley immature embryos with PC1104, we obtained five putative transgenic lines. Only three T_1_ families (No. 20, 34 and 35) segregated for their responses to PSH-72 (Table 2, Fig. 2c). T_1_ plants with functional *NLR*_*4DS-1*_, *RLK*_*4DS-1*_ and *RLK*_*4DS-2*_ were resistant (IT scores = 1-5), while the ones lacking the three genes were susceptible (IT scores = 7–8). Therefore, the fosmid PC1104 confers stripe rust resistance in transgenic wheat and barley.

Because PC1104 has *NLR*_*4DS-1*_, *RLK*_*4DS-1*_ and *RLK*_*4DS-2*_ genes, we cut PC1104 using a specific restriction enzyme (either *Bsr*GI, *Kpn*I, *Not*I, *Xba*I, or *Kpn*I + *Xba*I) to cleave/inactivate each of them, and then used the DNA fragments from each digestion to transform wheat separately (Supplementary Fig. 6). After bombarding 6,790 wheat immature embryos, we obtained 148 putative transgenic plants. Transgenic T_1_ and T_2_ plants were tested with *Pst* spores from the Parker Farm field (Moscow, ID, USA). Only transgenic plants that expressed *NLR*_*4DS-1*_ were resistant to *Pst* (Table 2, Supplementary Fig. 4). Therefore, *NLR*_*4DS-1*_ represents a strong candidate for *YrAS2388* (Table 2).

The *Pst*-resistant *NLR*_*4DS-1*_ has duplicated 3′UTRs (Fig. 1d) in all *Pst*-resistant parents (CIae9, PI511383 and PI511384) and each 3′UTR is associated with multiple transcript variants: TV1 and TV2 with 3′UTR1, TV3 and TV4a (and 4b) with 3′UTR2 (Fig. 1e). We overexpressed the *Pst*-resistant *NLR*_*4DS-1*_ cDNA under the maize *Ubi* promoter (Supplementary Table 4). All 36 transgenic wheat and barley lines that expressed TV1 (or TV2) did not confer resistance to stripe rust (Table 2), suggesting that one cDNA isoform was insufficient to confer stripe rust resistance. For stripe rust resistance, the *NLR*_*4DS-1*_ gene may require the activity of multiple cDNA isoforms and/or regulatory elements in the genomic sequence.

### Innate and external factors regulate *NLR*_*4DS-1*_ expression

In the *Pst*-resistant *NLR*_*4DS-1*_, the most abundant isoforms are TV1 (for a 1068-aa protein with complete 4HB, NB, and LRR domains) and TV4 (for a 471-aa protein with a complete 4HB and a partial NB domain) (Fig. 1e, Supplementary Fig. 7a). The less abundant isoform TV2 might result from either a partial exon skipping from TV1 or the retention of an 833-bp cryptic intron in exon 5, which disrupts the NB and LRR domains. TV3 is also a less abundant isoform and is structurally similar to TV4, but retains the first 244 bp in the 833-bp cryptic intron, which only disrupts the LRR domain. In contrast, the *Pst*-susceptible *NLR*_*4DS-1*_ either remained completely silent in PI486274 and PI560536 or produced only the TV1-type transcript in AL8/78 and AS87 (Supplementary Fig. 3b).

In *Pst*-resistant PI511383, TV1 to TV4 cDNAs were all expressed in the seedling and adult leaves (Supplementary Fig. 3c). When exposed to alternating low (10 °C) and high (25 °C) temperatures, the high temperature upregulated TV2 and downregulated TV4 (Supplementary Fig. 3d), which is correlated with increased *Pst*-resistance at elevated temperatures. In response to *Pst* race PSTv-306, the TV1 cDNA levels in the *Pst*-infected plants were comparable to those in the mock-inoculated control plants (Supplementary Fig. 3e). In contrast, *Pst* infections upregulated TV2 at 2, 5, and 10 days post inoculation (dpi) but not at 3 dpi, downregulated TV3 at 3, 7, and 14 dpi, and downregulated TV4 at 3 dpi (Supplementary Fig. 3e). Thus, both temperature and *Pst* infection regulate the transcription of *NLR*_*4DS-1*_. However, a change in the relative levels of either the individual four transcripts and/or the proteins or protein complexes may affect the induction of stripe rust resistance. Among the *Pst*-susceptible mutations of NLR_4DS*-1*_, Ser394Asn and Gln557Stop(*) only affect TV1 and Thr456Ile only affects TV4, which indicates that both TV1 and TV4 are essential for stripe rust resistance (Supplementary Data 4). Collectively, we hypothesize that TV1 plays a major role in the induction of stripe rust resistance, TV2 acts as a positive co-factor, and TV4 (or possibly TV3) act either as negative regulators when its expression is high or as positive regulators when its expression is low (Supplementary Fig. 8).

Using a yeast two-hybrid system, we tested the interaction among the native (TV1, TV2, and TV4) and mutant (TV1^G117D^, TV2^G117D^ and TV2^V267I^; Supplementary Data 4, Supplementary Fig. 7a) isoforms of the *Pst*-resistant *NLR*_*4DS-1*_. The NLR_4DS-1_ isoforms, both native and mutant forms (NM forms) had no autoactivity. A strong interaction occurred amongst the TV2 proteins (NM forms; Supplementary Fig. 7b). We observed a weak interaction between TV2 mutants and TV1 (NM forms), and between TV2 proteins (NM forms) and TV4. Apparently, TV2 can mediate protein interactions amongst multiple isoforms of NLR_4DS-1_.

### The *Pst*-resistant *NLR*_*4DS*-*1*_ occurs only in *Aegilops tauschii*

The D genome of common wheat was derived from *Ae. tauschii* subsp. *strangulata* or *tauschii*^20^. The resistance allele of *NLR*_*4DS-1*_ is present in 100% (*n* = 37) and 19% (*n* = 122) of the accessions of subsp. *strangulata* and *tauschii* tested, respectively (Supplementary Data 3). Similarly, the resistance allele of *NLR*_*4DS-1*_ is present in 30% (*n* = 23) of the *Ae. tauschii* accessions used as a parent in developing SHW lines (Supplementary Data 5). Surprisingly, the resistance allele is absent in all (*n* = 461) of the common wheat lines tested (Supplementary Table 5, Supplementary Data 6). The *NLR*_*4DS-1*_ allele in Chinese Spring (CS) is nearly identical to the *Pst*-susceptible alleles from the subsp. *tauschii* accessions PI486274 and PI560636 (Supplementary Data 2). In addition, the resistant *NLR*_*4DS-1*_ allele is also absent in all the tested *T. monococcum* subsp. *aegilopoides* (*n* = 24), *T. monococcum* subsp. *monococcum* (*n* = 24), *T. turgidum* subsp. *dicoccoides* (*n* = 140), *Ae. comosa* (*n* = 17), *Ae. comosa* var. *subventricosa* (*n* = *6*), *Ae. longissimi* (*n* = 8), *Ae. sharonensis* (*n* = 28), *Dasypyrum villosum* (*n* = 10), and *Hordeum vulgare* subsp. *spontaneum* (*n* = 5) (Supplementary Table 5, Supplementary Data 6).

### The *Pst*-resistant *NLR*_*4DS-1*_ may arise from paralogous genes

All *Pst*-resistant *NLR*_*4DS-1*_ genes contain two duplicated regions. The first region includes the 3′ end of exon 5, exons 6 and 7, and intron 7a; and the second region includes the pseudo-exon 5′, exons 6′ and 7′, and intron 7a′ (Fig. 1d, e, Supplementary Fig. 9a). This duplication is not present either in *Pst*-susceptible *NLR*_*4DS-1*_ alleles or in any *NLR*_*4DS-1*_-like genes. To examine the origin of the duplicated regions, we built separate phylogenetic trees for each of six selected fragments (exons 5, 6, 7, and 8; and introns 7a and 7b) of 7 to 15 *NLR*_*4DS-1*_ homologues in Triticeae (Supplementary Fig. 9b). The trees indicate that exons (5–8) and introns (7a and 7b) of the *Pst*-resistant *NLR*_*4DS-1*_ are more related to those of the *Pst*-susceptible *NLR*_*4DS-1*_ in CS (CS-4D:1821950..1825589); all the duplicated fragments (exons 5′ to 7′ and intron 7a′) are in separate clades. In addition, the duplicated 3′UTR1 and 3′UTR2 DNA of *NLR*_*4DS-1*_ in PI511383 are only 87% identical in the conserved 373 bp (GenBank MK736661: 3735..4107 versus 6409..6781, counted forward from the start codon ATG). Thus, the *Pst*-resistant *NLR*_*4DS-1*_ likely arose after a shuffling event between two paralogous genes. Specifically, the 3′UTR2 contains part of a 2668-bp insertion (within a 6-bp target site duplication = TACTGG) that occurred in intron 7 of the ancestral 3′UTR1 region. A similar 3′UTR duplication in the *Pst*-resistant *NLR*_*4DS-1*_ gene is present in the synthetic wheat W7984^35^. In the 2668-bp insertion, a 496-bp region (pseudo-exon exon 5′) is 90% identical to the ancestral exon 5. The insertion also has two miniature inverted-repeat transposable elements, which are frequently adjacent to transcriptionally active genes^36^. Likely, the 2668-bp fragment was derived from another, currently unidentified, *NLR*_*4DS-1*_ homologue in *Ae. tauschii*.

In Triticeae, there are multiple *NLR*_*4DS-1*_-like genes; three copies were identified in the *YrAS2388* region (Supplementary Fig. 2). In common wheat CS, there are at least five transcriptionally active homologues of the *NLR*_*4DS-1*_ gene (Supplementary Fig. 10). None of the *NLR*_*4DS-1*_-like homologues in CS has duplicated 3′UTRs. The *Pst*-susceptible *NLR*_*4DS-1*_ homologues in CS share only 86%-94% identity with the *Pst*-resistant *NLR*_*4DS-1*_ in PI511383 at the cDNA level.

### *NLR*_*4DS-1*_ offers a toolbox for solving stripe rust problems

We compared the stripe rust resistance in 81 SHW lines^33^ and their original parents, including 30 SHW lines with the *YrAS2388R* gene (Supplementary Data 5). *YrAS2388R* confers a strong *Pst* resistance (IT scores = 1–3) in *Ae. tauschii*^22^. However, 27% of SHW wheat had significantly less resistance than the parental lines (*T. turgidum* and/or *Ae. tauschii*). In this study, SW3 has the *Pst*-resistant *NLR*_*4DS-1*_ allele and shows the characteristic expression of alternatively spliced transcripts. However, SW3 was susceptible (IT scores = 7–9) to *Pst* in Moscow, ID, USA (Table 1), presumably because of a suppressor in its genetic background. Nonetheless, *Ae. tauschii* accessions with a strong *Pst* resistance frequently conferred moderate to high *Pst* resistance in a derived SHW wheat (Supplementary Data 5), indicating that *Ae. tauschii* is valuable for breeding resistant *NLR*_*4DS-1*_. For example, the SHW wheat Syn-SAU-S9 is based on Langdon/AS313//AS2399, in which the *Ae. tauschii* AS2399 is positive for the *YrAS2388R* gene^22^. Although Syn-SAU-S9 displayed only moderate resistance to *Pst* (IT scores = 4–5), we used Syn-SAU-S9 to transfer the *YrAS2388R* gene into common wheat. Three co-segregating markers were used for marker-assisted selection of *YrAS2388R* (Supplementary Fig. 11, Supplementary Table 2). In 2015, we developed an elite line Shumai 1675, which is an F_6_ line of Syn-SAU-S9/Chuan 07001//Shumai 969. Shumai 1675 is highly resistant to *Pst* in Sichuan, China. In 2017, Shumai 1675 outcompeted the check variety Mianmai 367 with an 11% increase in yield in the regional variety trials of the Sichuan province, China (Supplementary Table 6).

## Discussion

*YrAS2388R* provides robust resistance in a wide spatial and temporal range, including China (current study), Canada^37^, Norway^25^, the United Kingdom^38^ and the United States (TA2450 = CIae9, TA2452 = PI511384^39^; current study). However, *YrAS2388R* has had limited use probably for two reasons: it is absent in common wheat; and it can be suppressed in hexaploid wheat. In the present study, *YrAS2388R*, when separated from potential linkage drag, conferred strong stripe rust resistance in transgenic wheat and barley, indicating that *YrAS2388R* offers a practical solution for stripe rust resistance in Triticeae. The *YrAS2388R* gene-based markers (e.g. *Xsdauw95*, Supplementary Fig. 11) can be used for marker-assisted selection.

*YrAS2388R* is another example of a gene that was either not transferred or lost during domestication. Nevertheless, genes from both progenitors and distantly-related species of wheat can be used to enhance contemporary common wheat. Of the 81 permanently named *Yr* resistance genes, 21 were transferred from either related species or wild relatives of wheat, such as *Yr5* from *Triticum spelta*, *Yr15* and *Yr36* from *T. dicoccoides* and *Yr28* from *Ae. tauschii*^40^. However, alien genes can be accompanied by linkage drag^41^. For example, linked genes to *Yr8* from *Ae. comosa* are associated with tall height and delayed maturity^42^. The *Yr9* gene from the 1BL/1RS translocation improves grain yield but causes inferior quality^43^, which limits its use in wheat especially in the U.S. Pacific Northwest^44^. *YrAS2388R* could be transferred into wheat through a cisgenic approach. Thus, cisgenic *YrAS2388R* can provide an advantage to consumers in comparison to traditional breeding.

*YrAS2388R* (or *Pst*-resistant *NLR*_*4DS-1*_) is associated with duplicated 3′UTRs, which is an apparently rare phenomenon. The ancestral 3′UTR of *NLR*_*4DS-1*_ adjoined the 3′-end of an unknown *NLR*_*4DS-1*_ paralog, resulting in duplicated 3′UTRs in *Pst*-resistant *NLR*_*4DS-1*_. The 3′UTR is an important component of eukaryotic genes^45^. More than half of human genes use alternative polyadenylation to generate mRNAs that differ in the 3′UTR length but encode the same protein^46^. In contrast, there are few reports of genes with two separate 3′UTRs that cause a difference in the protein product. In wheat, the stripe rust resistance gene *WKS1* generated six transcript variants, of which *WKS1.1* differs from the others in the 3′UTRs^14^. *Pst*-resistant *NLR*_*4DS-1*_ also shows alternative splicing (AS) in the NB-LRR region of the gene. AS is prevalent in eukaryotes^47^; 95% of multi-exon genes in human^48^ and 44% of multi-exon genes in Arabidopsis^49^ display AS. In Arabidopsis, the bacterial-resistance gene *RPS4* produces alternative transcripts in response to infection by pathogen *Pseudomonas syringae* pv. *tomato*^50^. Both environmental and developmental stimuli precisely regulate the abundance of functional mRNA isoforms^51^. Here, in keeping with resistance, expression of the *NLR*_*4DS-1*_ isoforms also depends on pathogen infection and the temperature. Thus, abundance of *NLR*_*4DS-1*_ isoforms appears to be a mechanism that wheat can use to robustly resist stripe rust pathogen invasion.

The NLR_4DS-1_ protein is a member of the CC-NB-LRR (CNL) proteins. The coiled-coil domain of the potato virus X resistance protein (Rx) actually forms a four-helix bundle (4HB)^52^. The N-terminal domain of NLR_4DS-1_ is predicted to fold into four helixes, and it is also classified as an Rx-CC-like in the NCBI CDD (E = 9 × 10^−9^) and Rx_N in the Pfam database (E = 6 × 10^−16^). Although CNL genes are often race-specific and not durable^53^, some CNL genes such as the rice blast resistance gene *Pigm R*^54^ have been durable. Here, we showed that *YrAS2388R* confers resistance to a broad array of *Pst* races and has been effective to all natural infections of *Pst* in China since 1995. As a typical *NLR* gene, we hypothesize that the NLR_4DS-1_ proteins change their state via a competition model (Supplementary Fig. 8). The full-length TV1 protein plays a central role in signal transduction, but it requires other variant proteins (TV2 and TV4) for a proper conformation, which together form an active TV1 complex for defense signaling.

Here, *YrAS2388R* was fully expressed without suppression in transgenic hexaploid wheat and in barley. In addition, we have produced Shumai 1675, which has *YrAS2388R* and is strongly resistant to *Pst*, suggesting that either *YrAS2388R* is not suppressed in Shumai 1675 or that *YrAS2388R* worked positively with other *Yr* genes to confer resistance to *Pst*. However, in the current study, the resistance levels of parental lines (*T. turgidum* and/or *Ae. tauschii*) were suppressed in nearly 27% of the SHW wheat lines. *Yr28*, which is probably the same gene as *YrAS2388*^22^, was effective in seedlings and adult plants of SHW Altar 84/*Ae. tauschii* accession W-219^24^. Here, we observed that *YrAS2388R* in SHW SW3 was suppressed, i.e., it was fully susceptible to natural *Pst* races at adult-plant stages in Moscow (ID, USA), probably because the suppressor responds more to the cooler night temperatures in this area. When *YrAS2388R* is suppressed in a specific hexaploid wheat such as SW3, *Pst*-resistance levels might be increased by disrupting the unknown suppressor, as was previously done by inactivating a suppressor of stem rust resistance^55^.

In the case of wheat powdery mildew, pyramiding of closely related *NLR* genes can cause dominant-negative interactions and that lead to *R* gene suppression^56^. For example, the *Pm8* resistance gene from rye was suppressed in wheat by a susceptible allele of the wheat ortholog *Pm3*^57^. In the present study, the *Pst*-resistant *NLR*_*4DS-1*_ in PI511383 shares 86–94% identity with cDNA from the transcriptionally active homologues in common wheat (Supplementary Fig. 10). Thus, *YrAS2388R* suppression might conceivably be caused by close homologues of *NLR*_*4DS-1*_ that are present in Triticeae. To test this hypothesis, in the future, one could mutagenize a SW3 line, screen for truncation mutations in the *NLR*_*4DS-1*_ homologues, and test whether the homologues’ mutations have any effect on stripe rust resistance. Regardless, because the transgene *NLR*_*4DS-1*_ induces effective *Pst* resistance in hexaploid wheat, we predict that sustainable *Pst* resistance can be achieved with either a cisgenic strategy with *Pst*-resistant *NLR*_*4DS-1*_ or a conventional strategy that combines both the incorporation of a *Pst*-resistant *NLR*_*4DS-1*_ and either avoidance or inactivation of the apparently linked latent suppressor(s) from *Ae. tauschii*.

## Methods

### Plant materials

This study was performed on *Aegilops tauschii*, *Hordeum vulgare*, *Triticum aestivum* and synthetic hexaploid wheat (SHW^58^) (Supplementary Table 7). Sources of accessions used for haplotype analysis are indicated in Supplementary Data 3 and 6. To map *YrAS2388*, we used six *Ae. tauschii* accessions (Supplementary Table 7), in which the *Pst*-resistant parents, CIae9, PI511383 and PI511384 (= AS2388), all have *YrAS2388R*^22^.

We developed three F_2_ populations (popA: PI511383/PI486274; popB: CIae9/PI560536; and popC: PI511384/AS87^23^). These populations were used for preliminary and fine mapping, and popC was also used to confirm the single Mendelian inheritance of *YrAS2388*. In popA, we selected 11 F_2_ plants that were heterozygous in the *YrAS2388* region (*Xsdauw2*-*Xsdauw36*), and allowed them to self-pollinate to produce F_3_ seeds. After screening 4,205 F_3_ plants, we identified 467 plants with crossovers in the *Xsdauw2*-*Xsdauw36* interval, and used them to generate a high-density map.

### Stripe rust inoculum and infection assays

Wheat stripe rust tests were conducted in four institutions: Shandong Agricultural University (SDAU), Tai’an, China; Sichuan Agricultural University (SCAU), Chengdu, China; Washington State University (WSU), Pullman, USA; and University of Idaho (UI), Moscow, USA. Avocet Susceptible (AvS), Huixianhong, Mingxian 169, and/or SY95-71 were used as susceptible checks and also planted surrounding the plots to increase and spread urediniospores for adequate and uniform rust levels for reliable screening. For winter-growth genotypes tested in greenhouses or growth chambers, seeds were vernalized in wet germination paper (Anchor Paper Co., Saint Paul, MN, USA) at 4 °C in darkness for 45 d; vernalized shoots were transplanted into soil in the greenhouse and maintained at 25 °C during the day and 15 °C at night with 16 h photoperiod.

Infection types (IT) were recorded using a 0-9 scale^59^ and the following categories: resistant (R, IT scores = 0–3), moderate reactions (M, IT scores = 4–6) that include moderate resistance (MR, IT scores = 4–5) and moderate susceptibility (MS, IT score = 6), and susceptible (S, IT scores = 7–9). IT scores were recorded 15–18 days post inoculation (dpi) when the uredinial pustules were clearly visible on susceptible plants. Responses of SHW and their parental lines to *Pst* are shown in Supplementary Data 5.

At SDAU, urediniospores were obtained from the Institute of Plant Protection, Chinese Academy of Agricultural Sciences, Beijing, China. Due to changes in race frequency and spore availability, different *Pst* races were used in different years (mixed spores of Chinese *Pst* races CYR29, CYR31, CYR32, CYR33, Su11 and/or Su14 during 2010 to 2012; CYR29 and CYR32 in 2013; and CYR29, CYR31, CYR32 and CYR33 in 2014–2016). Collectively, these races represent the predominant *Pst* races in China in different periods since the 1990’s. Field trials were performed to assess the responses to *Pst* in the parental lines, F_1_, F_2_ and advanced progenies of popA and popB. At the seedling stage, an aqueous spore suspension was manually injected with a 2.5 ml syringe into leaf bundles and repeated after 10 days. For preliminary mapping, F_2_ plants of popA and popB were evaluated in 2011, and the corresponding F_3_ progeny were then tested in 2012. Critical recombinants of popA were evaluated in 2013-2016 (F_3_ to F_6_ generations, one generation per year), and the F_4_–F_6_ generations were additionally tested in SCAU in 2014–2016.

At SCAU, we primarily conducted the *Pst* test in Dujiangyan and Wenjiang, two experimental stations of the Triticeae Research Institute at SCAU. Urediniospores were obtained from the Research Institute of Plant Protection, Gansu Academy of Agricultural Sciences, Lanzhou, China. Using the mixture of Chinese *Pst* races CYR30, CYR31, CYR32, SY11-4, SY11-14, and HY46-8, we evaluated the *Ae. tauschii* germplasm in Dujiangyan for three growing seasons (2006–2009). In 2008–2009, we also tested synthetic wheat and their polyploid parents in Dujiangyan (Supplementary Data 5). Using a mixture of CYR30, CYR31, CYR32, CYR33, SY11-4 and HY46-8, we retested synthetic wheat and their parent lines in Wenjiang in 2011–2012 (Supplementary Data 5)^22^, and then retested five synthetic wheat and their parent lines in Wenjiang in 2016–2017 using a mixture of CYR32, CYR33, CYR34 (= Gui22-9), Gui22-14, and SY11-4 (Supplementary Data 5). To identify *Pst*-susceptible mutants, we screened the Syn-SAU-93 population in 2016–2018 and the SW3 population in 2017–2018 using urediniospores of similar races as 2016–2017. For popC, we assessed the parental lines, F_1_, F_2_ and advanced progenies from 2010 to 2016. In 2011, F_2_ plants were tested in Wenjiang, and the field plots were inoculated at 7 wk after planting with a mixture of CYR30, CYR31, CYR32, CYR33, SY11-4 and HY46-8.

At WSU, urediniospores were produced by the USDA-ARS Wheat Unit at Pullman, WA, USA. The plants were initially grown in a greenhouse at 15 to 25 °C. At the two-leaf stage, we prepared a mixture of urediniospore and talc at 1:20 ratio (v vs. v), dusted it on plants, and then applied a water mist onto the plants. The inoculated plants were incubated in a dew chamber at 10 °C in the dark for 24 h, and then moved to growth chambers for either low or high temperature tests. The low temperature (LT) cycle had a 16-h photoperiod (6 a.m.–10 p.m.) with a diurnal temperature cycle of 4 °C at 2 am and a gradual increase to 20 °C at 2 pm followed by a gradual decrease to 4 °C at 2 am. The high temperature (HT) cycle had a 16-h photoperiod with a gradual temperature gradient from 10 °C at 2 a.m. to 30 °C at 2 p.m. and then back to 10 °C at 2 a.m..

At UI, urediniospores were produced by the USDA-ARS Wheat Unit at Pullman, WA, USA. Transgenic plants and wild-type controls were grown in chambers. At either the two-leaf stage for seedlings or at the 6-leaf stage for adults, plants were dust-inoculated using the urediniospore and talc mixture (1:20), maintained at 10 °C for 48 h in dark, and then maintained under a modified LT cycle (10 °C for 12 h with 8-h of darkness in the middle, 20 °C for 8 h in the middle of 16-h light, with a gradual transition from 10 to 20 °C within a 2-h light period and vice versa for a gradual transition from 20 to 10 °C) or under a modified HT cycle (15 °C for 12 h with 8-h darkness in the middle, 25 °C for 8 h in the middle of a 16-h light, with a gradual transition from 15 to 25 °C within a 2-h light period, and vice versa for a gradual transition from 25 to 15 °C).

### Bulked segregant analysis of the *YrAS2388* gene

Genomic DNA was extracted using the Sarkosyl method^17^. Infinium iSelect genotyping was assayed at the Genome Center (University of California, Davis, CA, USA). Normalized Cy3 and Cy5 fluorescence for each DNA sample was plotted with the GenomeStudio program (Illumina, Inc., San Diego, CA, USA), resulting in genotype clustering for each SNP marker^20^.

We performed bulked segregant analysis (BSA) on four parents (CIae9, PI486274, PI511383 and PI560536) and 17 *Pst*-susceptible F_2_ plants, ten from popA and seven from popB (Supplementary Table 1), using the wheat 10k iSelect array^29^. The *Pst* responses of the tested plants were obtained in the field in 2011. For SNP data, we sequentially eliminated: (1) those with missing data or that were being heterozygous in the parents, (2) those that were being polymorphic between the two resistant parents or between the two susceptible lines, (3) those that were identical among the four parents, and (4) those with four or more missing data points amongst 17 *Pst*-susceptible F_2_ plants. We retained 3276 SNP loci for BSA analysis. Among *Pst*-susceptible F_2_ plants with a clear genotype, the frequency of a homozygous “B” genotype (= susceptible phenotype) was calculated and sorted in descending order for each SNP. The top 20 SNPs were prioritized for further analysis.

### Preliminary and fine mapping of the *YrAS2388* gene

We targeted the AT4D3406 region (Supplementary Fig. 1a) to develop PCR markers, which was facilitated by using the *Ae. tauschii* SNP map^29^, and the genome sequences of *Ae. tauschii*^30^, common wheat (IWGSC RefSeq v1.0^60^), synthetic wheat^35^ and 20 fosmid clones of PI511383. Markers were primarily based on insertion-deletion polymorphisms (InDel), cleaved amplified polymorphic sequences (CAPS) and derived cleaved amplified polymorphic sequences (dCAPS^61^). PCR primers, restriction enzymes and annealing temperatures are described in the Supplementary Table 2. All other oligos used in the current study are documented in the Supplementary Table 8. PCR products were separated in either 6% non-denaturing acrylamide or 2% agarose gels. The 4DS maps (Supplementary Fig. 1) were calculated using the maximum likelihood algorithm and the Kosambi function in JoinMap 4.0 (Kyazma B.V., Wageningen, Netherlands) and were assembled using MapChart v2.3 (www.wur.nl/en/show/Mapchart.htm).

### Construction and screening of the fosmid genomic library

PI511383 leaf tissue was harvested from 4-week-old plants and stored at −80 °C. Megabase-size DNA was prepared by embedding nuclei in 0.5% low-melting agarose, followed by nuclear lysis in the presence of detergent and proteinase-K^62^. Sixty DNA plugs were transferred to individual 1.5-ml tubes with 200-μl TE buffer. DNA in agarose was sheared by 22 freeze-thaw cycles with incubation in liquid nitrogen for 20 s and then a 45 °C water bath for 3 min. The sheared DNA in a 33.5–63.5-kb range was purified from a gel, repaired using the DNA End-Repair enzyme, ligated into the pCC1FOS vector, and packed into the phage particles as instructed by the CopyControl™ Fosmid Library Production Kit (Epicentre Technologies Corp., Madison, WI, USA). Packaged fosmid clones were transformed into the EPI300-T1R competent cells, and the titer of the genomic library was calculated as indicated in the manual (Epicentre). On average, 1,000 or 2,000 clones per plate were obtained from a diluted solution after an 18 h to 24 h incubation at 37 °C. Colonies were recovered using a mix with 6 ml LB and 1.8 ml glycerol, divided into three aliquots (2 ml each, super colony pools), and stored at −80 °C.

PCR screening was performed on each of 622 super colony pools, with 2-μl bacterium stock as template. We screened for markers *Xsdau93*, *Xsdau95* and *O13* (PCR primers P160/P161) (Supplementary Tables 2 and 8). PCR amplification was performed as follows: 95 °C for 5 min, 32 cycles with 95 °C for 30 s, 58 °C for 30 s and 72 °C for 50 s, and a final extension at 72 °C for 10 min. PCR products were separated on a 1% agarose gel and visualized by ethidium bromide staining. For positive super colony pools, 25-μl glycerol stock was inoculated into 5-ml liquid LB supplemented with 12.5 μg chloramphenicol ml^−1^ (LB-C), and cultured on a 250 rpm shaker at 37 °C for 4 h. The culture was diluted in a 10-fold series (10^−1^ to 10^−5^) using liquid LB, and the serial dilutions (300 μl per level) were plated onto the LB-C agar. An ideal dilution yielded 4,000–5,000 clones per 15-cm-diameter plate, from which colonies were collected using the 384-pin replicator with four repeated contacts to collect more representative colonies. After the replicator was used to inoculate a 384-well plate with 50-μl liquid LB-C, the plate was incubated at 37 °C overnight. Each well was screened by PCR. For positive wells, 20-μl culture was enriched in 2-ml liquid LB-C, and grown in a 250 rpm shaker at 37 °C for 2 h. The end culture was diluted 10-fold (from 10^−1^ to 10^−4^) using liquid LB-C, and 100-μl culture was plated onto LB-C agar. An ideal dilution yielded 50–200 clones per 9-cm-diameter plate, from which a positive clone would be revealed among 24 clones.

### Mutagenesis and mutation screening

Synthetic hexaploid wheat SW3 and Syn-SAU-93 were treated with 0.8% EMS (78 mM in water; Sigma-Aldrich Co., St. Louis, MO, USA). Briefly, lots of 400 seeds (M_0_) were soaked in 100 ml EMS solution, treated on a shaker at 150 rpm. at 25 °C for 10 h, and washed with running water at room temperatures for 4 h. M_1_ plants of SW3 were grown in a greenhouse in Taian, China. M_1_ plants of Syn-SAU-93 were grown in the field in Chongzhou, China. To simplify the fieldwork, mutant seeds of SW3 and Syn-SAU-93 were bulk planted at M_2_ to M_4_ generations in Chongzhou and Wenjiang, respectively, and screened for *Pst* resistance using mixed urediniospores of races CYR30, CYR31, CYR32, CYR33, Gui22-1, SY11-4, and HY46-8 in 2016–2018.

The *Pst*-susceptible mutants were screened for structural variations in the coding sequence of *RLK*_*4DS-1*_, *RLK*_*4DS-2*_, and *NLR*_*4DS-1*_. Plant DNA was prepared from the flag leaf using the Sarkosyl method^17^. Mutations of the candidate genes were identified using PCR-based DNA sequencing. For *RLK*_*4DS-1*_, we divided the 2570-bp fragment into two parts: (1) exons 1–3 between P162 and P163, and (2) exon 4 between P164 and P165. For *RLK*_*4DS-2*_, we examined a 1430-bp target region of the exon 3 between P167 and P168. For *NLR*_*4DS-1*_, we divided the 6072-bp fragment into five parts: (1) promoter and exons 1–3 between P169 and P170, (2) exon 4 between P171 and P172, (3) exon 5 between P173 and P174, (4) exon 5–6 between P175 and P176, and (5) the insertion region with 3′UTR2 between P177 and P178. PCR primers are described in the Supplementary Table 8. A standard PCR reaction was performed with Taq polymerase (Promega, Madison, WI, USA). The PCR products were sequenced by the Sangon Biotech Company (Chengdu, Sichuan, China).

### Genetic transformation of wheat and barley

The fosmid PC1104 (= F2-1; Supplementary Table 4) from PI511383 is about 47.7 kb (GenBank MK288012). PC1104 contains genomic copies of *RLK*_*4DS-1*_, *RLK*_*4DS-2*_ and *NLR*_*4DS-1*_. Both intact and/or the restriction enzyme-cleaved PC1104 were used for wheat and barley transformation. For genetic transformations, we used four plasmids: (1) PC1104 for native expression of *RLK*_*4DS-1*_, *RLK*_*4DS-2*_, and *NLR*_*4DS-1*_, (2) *Bsr*GI, *Not*I, *Xba*I, *Kpn*I, or *Xba*I + *Kpn*I-cleaved PC1104 for native expression of *RLK*_*4DS-1*_, *RLK*_*4DS-2*_ and *NLR*_*4DS-1*_, (3) PC1101 for overexpression of the *NLR*_*4DS-1 TV1*_ cDNA, and (4) PC1102 for overexpression of the *NLR*_*4DS-1 TV2*_ cDNA (Supplementary Table 4). To overexpress the candidate genes, we cloned the cDNA copies of *NLR*_*4DS-1 TV1*_ cDNA with PCR primers P181/P182 and *NLR*_*4DS-1 TV2*_ cDNA with PCR primers P181/P183. We then assembled two plant expression constructs: PC1101 (*Ubi::NLR*_*4DS-1 TV1-cDNA*_) and PC1102 (*Ubi::NLR*_*4DS-1 TV2-cDNA*_) (Supplementary Table 4). The fosmid PC1104 has no plant selection marker, and thus required co-transformation with PC174, which has the bialaphos (*BAR*) and hygromycin (*HYG*) selection markers both under the CaMV *35S* promoter (Supplementary Table 4). The other two plant expression constructs (PC1101 and PC1102) have both *BAR* and *HYG* selection markers on their T-DNA, and were used for direct transformation.

Standard methods for biolistic bombardment and tissue culture of wheat were used^63^. Using an intact fosmid PC1104, we bombarded 1,590 immature embryos of CB037 and generated 24 putative transgenic plants. Using the cleaved fosmid PC1104 (Supplementary Fig. 6), we bombarded 5,790 immature embryos of CB037 and 2,013 immature embryos of Bobwhite, and generated 197 and 20 putative transgenic plants, respectively. Using the bombardment protocol for wheat^63^, we also transferred the intact fosmid PC1104 into barley Golden Promise, however the tissue culture and regeneration procedures were specific for barley^64^. We bombarded 2,200 immature embryos of Golden Promise and generated 300 putative transgenic plants.

We also overexpressed the *NLR*_*4DS-1*_ cDNA under the maize *Ubi* promoter in wheat and barley. For the *NLR*_*4DS-1 TV1*_ cDNA (in PC1101), we bombarded 2,200 wheat immature embryos (Bobwhite or CB037), obtained 40 putative T_0_ plants, and tested nine *NLR*_*4DS-1 TV1*_ expressing T_1_ families (seven of Bobwhite and two of CB037) for their response to PSTv-40. Using a standard *Agrobacterium*-mediated transformation^64^, we then infected 800 barley immature embryos (Golden Promise), obtained 15 putative transgenic T_0_ plants, and tested four *NLR*_*4DS-1 TV1*_ expressing T_1_ families against PSH-72. For *NLR*_*4DS-1 TV2*_ cDNA (PC1102), we bombarded 2,500 wheat immature embryos (Bobwhite), obtained 54 putative T_0_ plants, and tested ten *NLR*_*4DS-1 TV2*_ expressing T_1_ families for their response to PSTv-40. We also then infected 800 barley immature embryos (Golden Promise), obtained 28 putative transgenic T_0_ plants and tested 13 *NLR*_*4DS-1 TV2*_ expressing T_1_ families against PSH-72.

Transgene integration was confirmed by a positive amplification of *BAR* with primers P184/P185, *RLK*_*4DS-1*_ with primers P208/P209, *RLK*_*4DS-2*_ with primers P203/P204 and *NLR*_*4DS-1*_ with primers P213/P214 (or in the overexpression experiment with primers P186/P190). Transcription was assessed by RT-PCR with primers P208/P209 for *RLK*_*4DS-1*_, primers P187/P188 for *RLK*_*4DS-2*_, and primers P189/P190 for *NLR*_*4DS-1*_. *ACTIN* primers P191/P192 were used as an internal control for both wheat and barley. PCR primers are described in Supplementary Table 8.

### Haplotype analysis

Haplotype analysis was performed to understand the association of haplotypes and responses to *Pst* and the evolution of the *YrAS2388* region. Haplotype markers (HTM) were specifically designed for *RLK*_*4DS-1*_, *RLK*_*4DS-2*_ and *NLR*_*4DS-1*_. Their physical locations (in Supplementary Tables 3 and 5, Supplementary Data 3) are counted from “A” in the start codon (ATG) in the genomic allele (GenBank accession number MK288012); for each marker, two periods separate the starting and ending nucleotides, and a minus sign indicates a backward count from “A” and a plus sign indicates a forward count from “A”. First, 159 *Ae. tauschii* accessions were genotyped in Sichuan, China using seven markers: HTM1a (= *RLK*_*4DS-1*_), HTM2a (= *RLK*_*4DS-2*_) and HTM3a to HTM3e (= *NLR*_*4DS-1*_) (Supplementary Table 3, Supplementary Data 3). Second, 874 Triticeae lines were genotyped in Shandong, China using four markers: HTM1b (= *RLK*_*4DS-1*_), HTM2b (= *RLK*_*4DS-2*_) and HTM3f to HTM3g (= *NLR*_*4DS-1*_). PCR primers are described in Supplementary Table 2. Markers used to genotype the Triticeae collection in Shandong were different from those used for genotyping the *Ae. tauschii* collection in Sichuan. Genotypes per gene per accession were not necessarily identical between the two tested collections. Thus, grouping of haplotypes should be considered separately for these two collections.

### Gene expression analysis

RT-PCR was used to detect the expression of *RLK1*_*4DS-1*_, *RLK1*_*4DS-2*_, *NLR*_*4DS-1*_ and *ACTIN* (internal control). Plants were maintained at 25 °C during the day and 15 °C at night with a 16 h photoperiod. Total RNA was extracted using TRIzol (Life Technologies, Grand Island, NY, USA). First strand cDNA was synthesized using the M-MLV Reverse Transcriptase (Invitrogen, Carlsbad, CA, USA). RT-PCR was conducted on the 2^nd^-leaf of the juvenile (two-leaf stage) plants. Primers used were P193/P194 for *RLK*_*4DS-1*_, P195/P196 for *RLK*_*4DS-2*_, P197/P198 for *NLR*_*4DS-1*_ and P191/P192 for *ACTIN*. Phusion High-Fidelity DNA Polymerase (Thermo Scientific, Wilmington, DE, USA) was used to perform the PCR reaction with 30 cycles for *ACTIN*, *NLR*_*4DS-1*_ and *RLK*_*4DS-1*_ and 38 cycles for *RLK*_*4DS-2*_.

Quantitative real-time PCR (qRT-PCR) was used to measure four transcript variants of *NLR*_*4DS-1*_. qRT-PCR was conducted with SYBR Green reagents (Applied Biosystems, Foster City, CA, USA) on a StepOne Plus PCR System (Applied Biosystems). Specific PCR primers (Supplementary Table 8) were designed for four identified transcripts of the *NLR*_*4DS-1*_ gene. Wheat *ACTIN* was used as an endogenous control^65^. Primers used were P215/P216 for *NLR*_*4DS-1 TV1*_, P217/P218 for *NLR*_*4DS-1 TV2*_, P219/P220 for *NLR*_*4DS-1 TV3*_, P221/P222 for *NLR*_*4DS-1 TV4+*_ and P223/P224 for *ACTIN*. TV4^+^ also contains TV3, but TV3 only accounts for 2–5% of the total transcripts. The primer efficiencies were between 90% and 105%. Transcript levels were expressed as linearized fold-*ACTIN* levels calculated by the formula 2^(*ACTIN* CT – *TARGET* CT)^. Six biological replicates were used for each data point. Data were analyzed using the SAS program (v9.4).

### Sequence analysis

Fosmid clones were extracted using QIAGEN Large Construct Kits (QIAGEN, Germantown, MD, USA). Library preparation, high-throughput sequencing and quality control were performed by the Berry Genomics Company (Beijing, China). In brief, DNA was fragmented, end-repaired, ligated to Illumina adaptors, and separated on a 2% agarose gel to select fragments about 400–500 bp^66^. Adaptor specific primers were used to amplify the ligation products. The final library was evaluated by qRT-PCR. PE reads (150 bp) were obtained using the Illumina HiSeq2500. Sequence reads of the vector pCC1FOS and the bacterial genome were masked by the crossmatch tool in the Phrap package^67^. A de novo assembly of each fosmid was done using either SPAdes 3.12 [http://cab.spbu.ru/software/spades/] or ABySS 2.0.2 [www.bcgsc.ca/platform/bioinfo/software/abyss/releases/2.0.2]. Orientation and order of small contigs was inferred using the reference sequences of W7984^35^ and AL8/78^30^. Sequence gaps were filled by PCR clones and Sanger sequencing. Candidate genes were identified by searching Ensemble Plants [http://plants.ensembl.org/index.html] and the NCBI databases [http://www.ncbi.nlm.nih.gov/]. The secondary and three-dimensional structures of the NLR_4DS-1_ protein were predicted using PSIPRED [http://bioinf.cs.ucl.ac.uk/psipred/] and Phyre2 [http://www.sbg.bio.ic.ac.uk/phyre2/], respectively.

### Reporting summary

Further information on research design is available in the Nature Research Reporting Summary linked to this article.

## Supplementary information

Supplementary Information

Peer Review

Reporting Summary

Description of Additional Supplementary Files

Supplementary Dataset 1

Supplementary Dataset 2

Supplementary Dataset 3

Supplementary Dataset 4

Supplementary Dataset 5

Supplementary Dataset 6

Source Data

## Data Availability

Data supporting the findings of this work are available within the paper and its Supplementary Information files. A reporting summary for this Article is available as a Supplementary Information file. GenBank accessions include MK288012 for the fosmid PC1104 (= F2-1), MK288013 for the *YrAS2388R* contig, MK736661 for the *YrAS2388R* gene in PI511383, MK736662 for the *YrAS2388R* gene in CIae9, MK736663 for the *YrAS2388R* gene in PI511384 (= AS2388), MK736664 for the *YrAS2388S* gene in PI560536, MK736665 for the *YrAS2388S* gene in PI486274, and MK736666 for the *YrAS2388S* gene in AS87. Source data underlying Figs. 1a and 2, as well as Supplementary Figs. 3–5, 7b, and 11 are provided as a Source Data file. All datasets generated and analyzed during the current study are available from the corresponding author upon request.

## References

[1] WAP. World agricultural production. In: *Circular Series, WAP 03-19*. (United States Department of Agriculture—Foreign Agricultural Service, 2019). https://apps.fas.usda.gov/psdonline/circulars/production.pdf.

[2] Shewry PR, Hey SJ (2015). The contribution of wheat to human diet and health. Food Energy Secur..

[3] United-Nations. World population prospects: The 2015 revision, Key findings and advance tables. (Department of Economic and Social Affairs PD, United Nations, 2015). https://www.un.org/en/development/desa/publications/world-population-prospects-2015-revision.html.

[4] Chen W, Wellings C, Chen X, Kang Z, Liu T (2014). Wheat stripe (yellow) rust caused by *Puccinia striiformis* f. sp. *tritici*. Mol. Plant Pathol..

[5] Solh M., Nazari K., Tadesse W., Wellings C. R. The growing threat of stripe rust worldwide. In: *Borlaug Global Rust Initiative, 2012 Technical Workshop, Beijing, China* (ed. McIntosh R. A.) (Borlaug Global Rust Initiative, 2012). https://www.globalrust.org/sites/default/files/posters/solh_2012.pdf.

[6] Ali S (2017). Yellow rust epidemics worldwide were caused by pathogen races from divergent genetic lineages. Front. Plant Sci..

[7] Chen XM (2005). Epidemiology and control of stripe rust (*Puccinia striiformis* f. sp. *tritici*) on wheat. *Canadian*. J. Plant Pathol..

[8] de Vallavieille-Pope C (2011). Virulence dynamics and regional structuring of Puccinia striiformis f. sp. tritici in France between 1984 and 2009. Plant Dis..

[9] Bayles RA, Flath K, Hovmøller MS, Vallavieille-Pope Cd (2000). Breakdown of the *Yr17* resistance to yellow rust of wheat in northern Europe. Agronomie.

[10] Chen X (2013). High-temperature adult-plant resistance, key for sustainable control of stripe rust. Am. J. Plant Sci..

[11] Gessese M, Bariana H, Wong D, Hayden M, Bansal U (2019). Molecular mapping of stripe rust resistance gene *Yr81* in a common wheat landrace Aus27430. Plant Dis..

[12] Marchal C (2018). BED-domain-containing immune receptors confer diverse resistance spectra to yellow rust. Nat. Plants.

[13] Klymiuk V (2018). Cloning of the wheat *Yr15* resistance gene sheds light on the plant tandem kinase-pseudokinase family. *Nature*. Communications.

[14] Fu D (2009). A kinase-START gene confers temperature-dependent resistance to wheat stripe rust. Science.

[15] Krattinger SG (2009). A putative ABC transporter confers durable resistance to multiple fungal pathogens in wheat. Science.

[16] Moore JW (2015). A recently evolved hexose transporter variant confers resistance to multiple pathogens in wheat. Nat. Genet..

[17] Yuan C (2012). Distribution, frequency and variation of stripe rust resistance loci *Yr10*, *Lr34/Yr18* and *Yr36* in Chinese wheat cultivars. J. Genet. Genom..

[18] Wan A, Chen X (2014). Virulence characterization of *Puccinia striiformis* f. sp. *tritici* using a new set of *Yr* single-gene line differentials in the United States in 2010. Plant Dis..

[19] Chen X, Penman L, Wan A, Cheng P (2010). Virulence races of *Puccinia striiformis* f. sp. *tritici* in 2006 and 2007 and development of wheat stripe rust and distributions, dynamics, and evolutionary relationships of races from 2000 to 2007 in the United States. *Canadian*. J. Plant Pathol..

[20] Wang J (2013). *Aegilops tauschii* single nucleotide polymorphisms shed light on the origins of wheat D-genome genetic diversity and pinpoint the geographic origin of hexaploid wheat. New Phytol..

[21] McFadden ES, Sears ER (1946). The origin of *Triticum spelta* and its free-threshing hexaploid relatives. J. Hered..

[22] Liu M (2013). Stripe rust resistance in *Aegilops tauschii* germplasm. Crop Sci..

[23] Huang L (2011). Molecular tagging of a stripe rust resistance gene in *Aegilops tauschii*. Euphytica.

[24] Singh RP, Nelson JC, Sorrells ME (2000). Mapping *Yr28* and other genes for resistance to stripe rust in wheat. Crop Sci..

[25] Zhang R (2018). Two main stripe rust resistance genes identified in synthetic-derived wheat line Soru#1. Phytopathology.

[26] Zhang H-Q, Jia J-Z, Yang H, Zhang B-S (2008). SSR mapping of stripe rust resistance gene from *Ae. tauschii*. Hered. (Beijing).

[27] Ogbonnaya F. C., et al. Synthetic hexaploids: Harnessing species of the primary gene pool for wheat improvement. In: *Plant Breeding Reviews* (ed. Janick J.) John Wiley & Sons, Inc (2013). https://onlinelibrary.wiley.com/doi/abs/10.1002/9781118497869.ch2.

[28] GHJ Kema, Lange W, CHV Silfhout (1995). Differential suppression of stripe rust resistance in synthetic wheat hexaploids derived from *Triticum turgidum* subsp. *dicoccoides* and *Aegilops squarrosa*. Phytopathology.

[29] Luo M-C (2013). A 4-gigabase physical map unlocks the structure and evolution of the complex genome of *Aegilops tauschii*, the wheat D-genome progenitor. Proc. Natl. Acad. Sci..

[30] Luo M-C (2017). Genome sequence of the progenitor of the wheat D genome *Aegilops tauschii*. Nature.

[31] Afzal AJ, Wood AJ, Lightfoot DA (2008). Plant receptor-like serine threonine kinases: Roles in signaling and plant defense. Mol. Plant-Microbe Interact..

[32] Friesen TL, Xu SS, Harris MO (2008). Stem rust, tan spot, stagonospora nodorum blotch, and hessian fly resistance in Langdon durum-*Aegilops tauschii* synthetic hexaploid wheat lines. Crop Sci..

[33] Zhang L-Q (2010). Frequent occurrence of unreduced gametes in *Triticum turgidum*–*Aegilops tauschii* hybrids. Euphytica.

[34] Peña PA (2017). Molecular and phenotypic characterization of transgenic wheat and sorghum events expressing the barley alanine aminotransferase. Planta.

[35] Chapman JA (2015). A whole-genome shotgun approach for assembling and anchoring the hexaploid bread wheat genome. Genome Biol..

[36] Keidar-Friedman D, Bariah I, Kashkush K (2018). Genome-wide analyses of miniature inverted-repeat transposable elements reveals new insights into the evolution of the *Triticum-Aegilops* group. PLOS ONE.

[37] Brar GS, Dhariwal R, Randhawa HS (2018). Resistance evaluation of differentials and commercial wheat cultivars to stripe rust (*Puccinia striiformis*) infection in hot spot regions of Canada. Eur. J. Plant Pathol..

[38] Knaggs P, Ambrose MJ, Reader SM, Miller TE (2000). Morphological characterisation and evaluation of the subdivision of *Aegilops tauschii* Coss. Wheat Inf. Serv..

[39] Yildirim A, Jones SS, Murray TD, Cox TS, Line RF (1995). Resistance to stripe rust and eyespot diseases of wheat in *Triticum tauschii*. Plant Dis..

[40] Wang Meinan, Chen Xianming (2017). Stripe Rust Resistance. Stripe Rust.

[41] Klindworth DL, Hareland GA, Elias EM, Xu SS (2013). Attempted compensation for linkage drag affecting agronomic characteristics of durum wheat 1AS/1DL translocation lines. Crop Sci..

[42] Riley R, Chapman V, Johnson ROY (1968). Introduction of yellow rust resistance of *Aegilops comosa* into wheat by genetically induced homoeologous recombination. Nature.

[43] Oak MD, Tamhankar SA (2017). 1BL/1RS translocation in durum wheat and its effect on end use quality traits. J. Plant Biochem. Biotechnol..

[44] Qie Y (2018). Development, validation, and re-selection of wheat lines with pyramided genes *Yr64* and *Yr15* linked on the short arm of chromosome 1B for resistance to stripe rust. Plant Dis..

[45] Kuersten S, Goodwin EB (2003). The power of the 3’ UTR: translational control and development. Nat. Rev. Genet..

[46] Lianoglou S, Garg V, Yang JL, Leslie CS, Mayr C (2013). Ubiquitously transcribed genes use alternative polyadenylation to achieve tissue-specific expression. Genes Dev..

[47] Kornblihtt AR (2013). Alternative splicing: a pivotal step between eukaryotic transcription and translation. Nat. Rev. Mol. Cell Biol..

[48] Pan Q, Shai O, Lee LJ, Frey BJ, Blencowe BJ (2008). Deep surveying of alternative splicing complexity in the human transcriptome by high-throughput sequencing. Nat. Genet..

[49] Howard BE (2013). High-throughput RNA sequencing of *Pseudomonas*-infected Arabidopsis reveals hidden transcriptome complexity and novel splice variants. PLOS ONE.

[50] Zhang X-C, Gassmann W (2007). Alternative splicing and mRNA levels of the disease resistance gene *RPS4* are induced during defense responses. Plant Physiol..

[51] Yan L, Thomas E (2018). Transcript-level expression control of plant NLR genes. Mol. Plant Pathol..

[52] Hao W, Collier SM, Moffett P, Chai J (2013). Structural basis for the interaction between the potato virus X resistance protein (Rx) and its cofactor Ran GTPase-activating protein 2 (RanGAP2). J. Biol. Chem..

[53] Maekawa T, Kufer TA, Schulze-Lefert P (2011). NLR functions in plant and animal immune systems: So far and yet so close. Nat. Immunol..

[54] Deng Y (2017). Epigenetic regulation of antagonistic receptors confers rice blast resistance with yield balance. Science.

[55] Williams ND, Miller JD, Klindworth DL (1992). Induced mutations of a genetic suppressor of resistance to wheat stem rust. Crop Sci..

[56] Daniel S (2014). Suppression among alleles encoding nucleotide-binding–leucine-rich repeat resistance proteins interferes with resistance in F_1_ hybrid and allele-pyramided wheat plants. Plant J..

[57] Hurni S (2014). The powdery mildew resistance gene *Pm8* derived from rye is suppressed by its wheat ortholog. Pm3. Plant J..

[58] Baker RJ, Dyck PL (1974). Combining ability for yield of synthetic hexaploid wheats. Can. J. Plant Sci..

[59] Line RF, Qayoum A (1992). Virulence, aggressiveness, evolution, and distribution of races of *Puccinia striiformis* (the cause of stripe rust of wheat) in North America, 1968-87. US Dep. Agric Tech. Bull..

[60] IWGSC. (2018). Shifting the limits in wheat research and breeding using a fully annotated reference genome. Science.

[61] Neff MM, Neff JD, Chory J, Pepper AE (1998). dCAPS, a simple technique for the genetic analysis of single nucleotide polymorphisms: experimental applications in *Arabidopsis thaliana* genetics. Plant J..

[62] Luo M., Wing R. A. An improved method for plant BAC library construction. In: *Methods in Molecular Biology* (ed. Grotewold E.) (2003). https://link.springer.com/protocol/10.1385%2F1-59259-413-1%3A3. 10.1385/1-59259-413-1:3 14501055

[63] Lv B (2014). Characterization of *FLOWERING LOCUS T1* (*FT1*) gene in *Brachypodium* and wheat. PLoS ONE.

[64] Harwood W. A., et al. Barley transformation using *Agrobacterium*-mediated techniques. In: *Transgenic Wheat, Barley And Oats: Production And Characterization Protocols* (eds Jones D. H., Shewry R. P.) (Humana Press, 2009). https://link.springer.com/protocol/10.1007/978-1-59745-379-0_9.

[65] Fu D, Dunbar M, Dubcovsky J (2007). Wheat VIN3-like PHD finger genes are up-regulated by vernalization. Mol. Genet. Genom..

[66] Ni F (2017). Wheat *Ms2* encodes for an orphan protein that confers male sterility in grass species. *Nature*. Communications.

[67] Ewing B, Hillier L, Wendl MC, Green P (1998). Base-calling of automated sequencer traces using Phred. I. Accuracy assessment. Genome Res..

[68] Christensen AH, Sharrock RA, Quail PH (1992). Maize polyubiquitin genes: Structure, thermal perturbation of expression and transcript splicing, and promoter activity following transfer to protoplasts by electroporation. Plant Mol. Biol..

