# Supplementary material for: An ancestral NB-LRR with duplicated 3′UTRs confers stripe rust resistance in wheat and barley
Source: Nat Commun. 2019 Sep 6;10:4023. doi: 10.1038/s41467-019-11872-9 (PMC6731223; doi:10.1038/s41467-019-11872-9)
Supplement: Supplementary file 1 — Supplementary Information [file 41467_2019_11872_MOESM1_ESM.pdf]

# An ancestral NB-LRR with duplicated 3'UTRs confers stripe rust resistance in wheat and barley

Zhang *et al.*

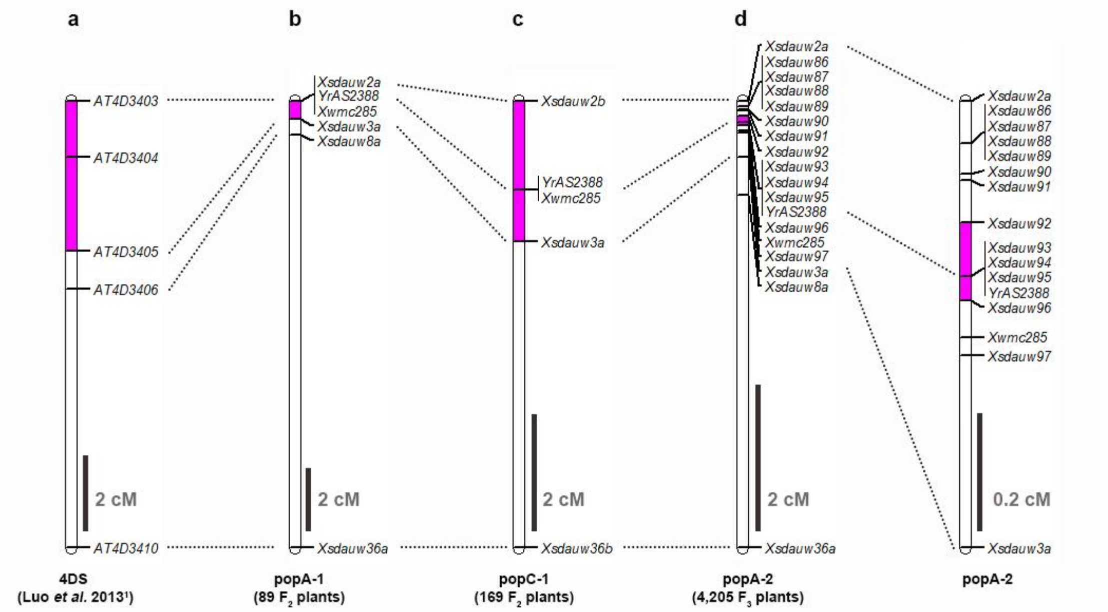


**Supplementary Fig. 1. Mapping of *YrAS2388* in *Aegilops tauschii* lines.** (**a**) The 4DS linkage map of *Aegilops tauschii*[1]. (**b**) A preliminary map from popA-1 (PI511383/PI486274; 89 $F_2$ plants), in which *Xsdauw2a* was completely linked to *YrAS2388*. (**c**) A preliminary map from popC-1 (PI511384/AS87; 169 $F_2$ plants) (**d**) Fine map of the *YrAS2388* region based on popA-2 (PI511383/PI486274; 4,205 $F_3$ plants). A high-resolution map of the *Xsdauw2a*-*Xsdauw3a* interval is illustrated in d. The magenta region represents the *YrAS2388* interval. On this map, *Xsdauw2*[2] and *Xsdauw3a* were developed from gene homologues of $NLR_{4DS-1}$. Variants of the same marker, e.g. *Xsdauw2a* and *Xsdauw2b*, represent the same locus/gene, but are population-specific.

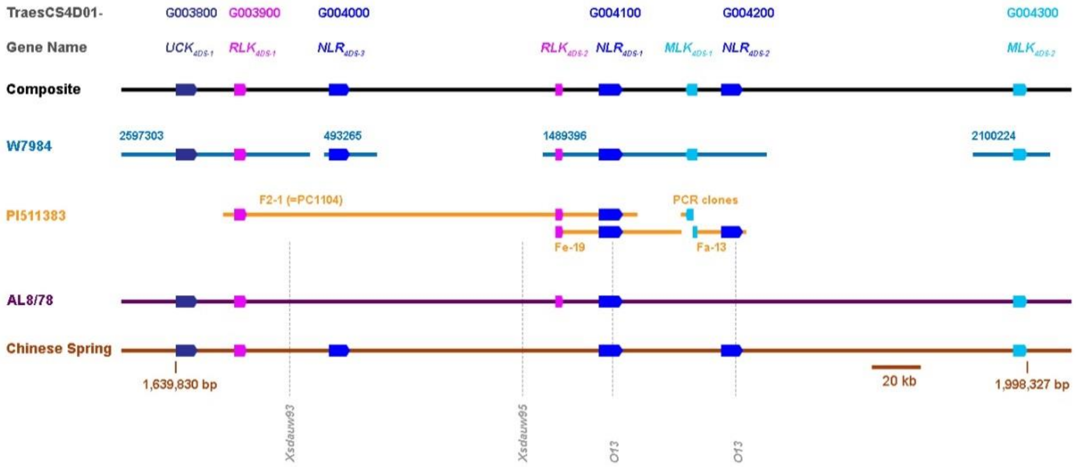


**Supplementary Fig. 2. Physical maps of the *YrAS2388* region.** We illustrate the *YrAS2388* region in four selected genotypes: synthetic hexaploid wheat W7984 (in blue; four scaffolds each with an ID); *Aegilops tauschii* accessions PI511383 (in orange) and AL8/78 (in purple); and common wheat Chinese Spring (in brown). Genes of the same type are displayed in the same color. Three markers, *Xsdau93*, *Xsdau95* and *O13*, were used to screen the PI511383 fosmid DNA library. *O13* was originally designed to amplify the $NLR_{4DS-1}$ gene with primers P160/P161 (Supplementary Table 8); *O13* is present in both the $NLR_{4DS-1}$ and the $NLR_{4DS-2}$ genes. Representative fosmid clones F2-1 (=PC1104), Fe-19 and Fa-13 were sequenced; a physical gap between Fe-19 and Fa-13 was closed by PCR clones and DNA sequencing. The final assembly of the *YrAS2388R* region in PI511383 spans 97.5 kb. A composite map (the top diagram in black) was made using the sequence data from W7984[3] (four scaffolds in blue), PI511383 (three fosmids in orange), AL8/78[4] (one contig in purple) and Chinese Spring (one contig in brown; IWGSC RefSeq v1.0[5]). The exact location (in bp) of two genes is labelled on the chromosome 4D in Chinese Spring. TraesCS4D01- shows the gene number in Chinese Spring[5]. Genes of interest include 4HB (formerly CC)-NB-LRR (NLR), malectin-like kinase (MLK), receptor-like kinase (RLK) and U-box containing protein kinase (UCK). The chromosome length and gene size in Chinese Spring are drawn in scale.

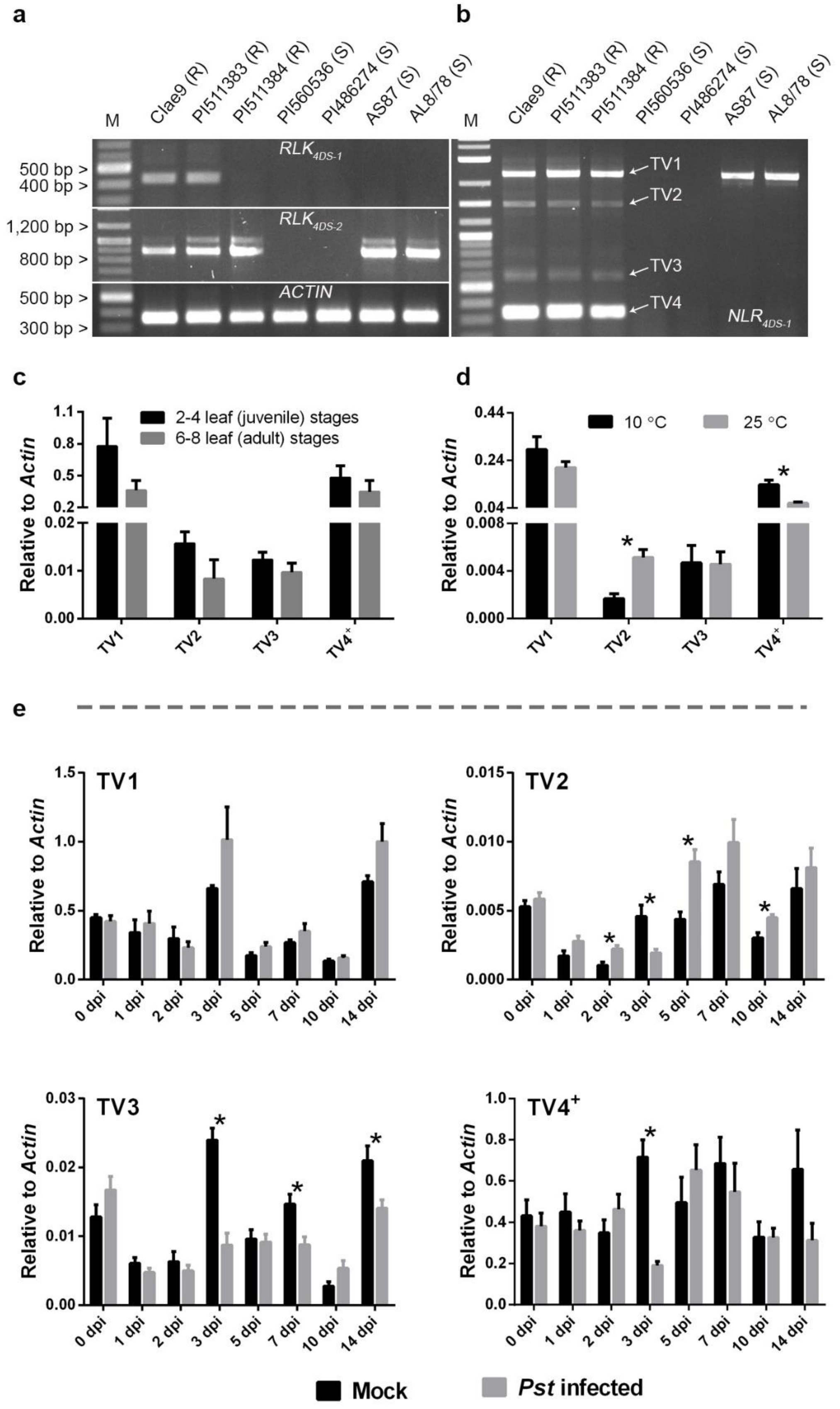
a
b
M
Clae9 (R)
PI511383 (R)
PI511384 (R)
PI560536 (S)
PI486274 (S)
AS87 (S)
AL8/78 (S)
500 bp >
400 bp >
1,200 bp >
800 bp >
300 bp >
RLK4DS-1
RLK4DS-2
ACTIN
TV1
TV2
TV3
TV4
NLR4DS-1
c
d
2-4 leaf (juvenile) stages
6-8 leaf (adult) stages
10 °C
25 °C
Relative to Actin
TV4+
e
Mock
Pst infected
0 dpi
1 dpi
2 dpi
3 dpi
5 dpi
7 dpi
10 dpi
14 dpi

**Supplementary Fig. 3. cDNA expression of the *YrAS2388* candidate genes in *Aegilops tauschii*.** (**a-b**) RT-PCR in selected genotypes. Plant responses (R=resistant; S=susceptible) to *Puccinia striiformis* f. sp. *tritici* (*Pst*) are indicated in parentheses. Target genes were (**a**) $RLK1_{4DS\text{-}1}$, $RLK1_{4DS\text{-}2}$ and *ACTIN* (internal control) and (**b**) $NLR_{4DS\text{-}1}$. TV4 from $NLR_{4DS\text{-}1}$ includes both TV4a and TV4b transcripts. Source data are provided as a Source Data file. (**c-e**) Quantitative real-time PCR of four $NLR_{4DS\text{-}1}$ isoforms in PI511383. $TV4^{+}$ also contains TV3, but TV3 only accounts for 2-5% of the total transcripts. Pairs of treatments (juvenile- vs. adult- stages; 10 °C vs. 25 °C; *Pst*- vs. mock-inoculation) that are marked with an asterisk are significantly different ($\alpha$=0.05) by Student's *t*-test on n=93 to 384 biologically independent samples. Error bars are one standard error of the mean (s.e.m.); they were based on independent datasets. (**c**) Developmental responses were conducted on newly expanded leaves at the juvenile (2 and 4-leaf) and adult (6 and 8-leaf) plants that were maintained at 25 °C during the day and 15 °C at night with a 16 h photoperiod. (**d**) Temporal responses were conducted on the 4th-leaf at the 5-leaf stage. Plants were alternated every 24h between 10 °C (day 1 and day 3) and 25 °C (day 2 and day 4), each with a 16-h photoperiod. (**e**) Responses to *Pst* were determined at the 2-leaf stage. After plants were mock- or inoculated with PSTv-306, they were maintained at 10 °C in darkness for 48 h, and were then maintained under a low temperature cycle (4 °C/20 °C) with a 16-h photoperiod. A mock- or inoculated leaf was sampled after 1, 2, 3, 5, 7, 10 and 14 days post inoculation (dpi). Six biological replicates were used for each data point. Source data are provided as a Source Data file. (**a-e**) All samples except those at 1 and 2 dpi were collected in the middle of the indicated photoperiod. Samples at 1 and 2 dpi were collected in the 10 °C dark cycle. Other abbreviations include base pairs (bp) and molecular standard (M=2-Log DNA Ladder, New England Biolabs, Beverly, MA, USA). Source data are provided as a Source Data file.

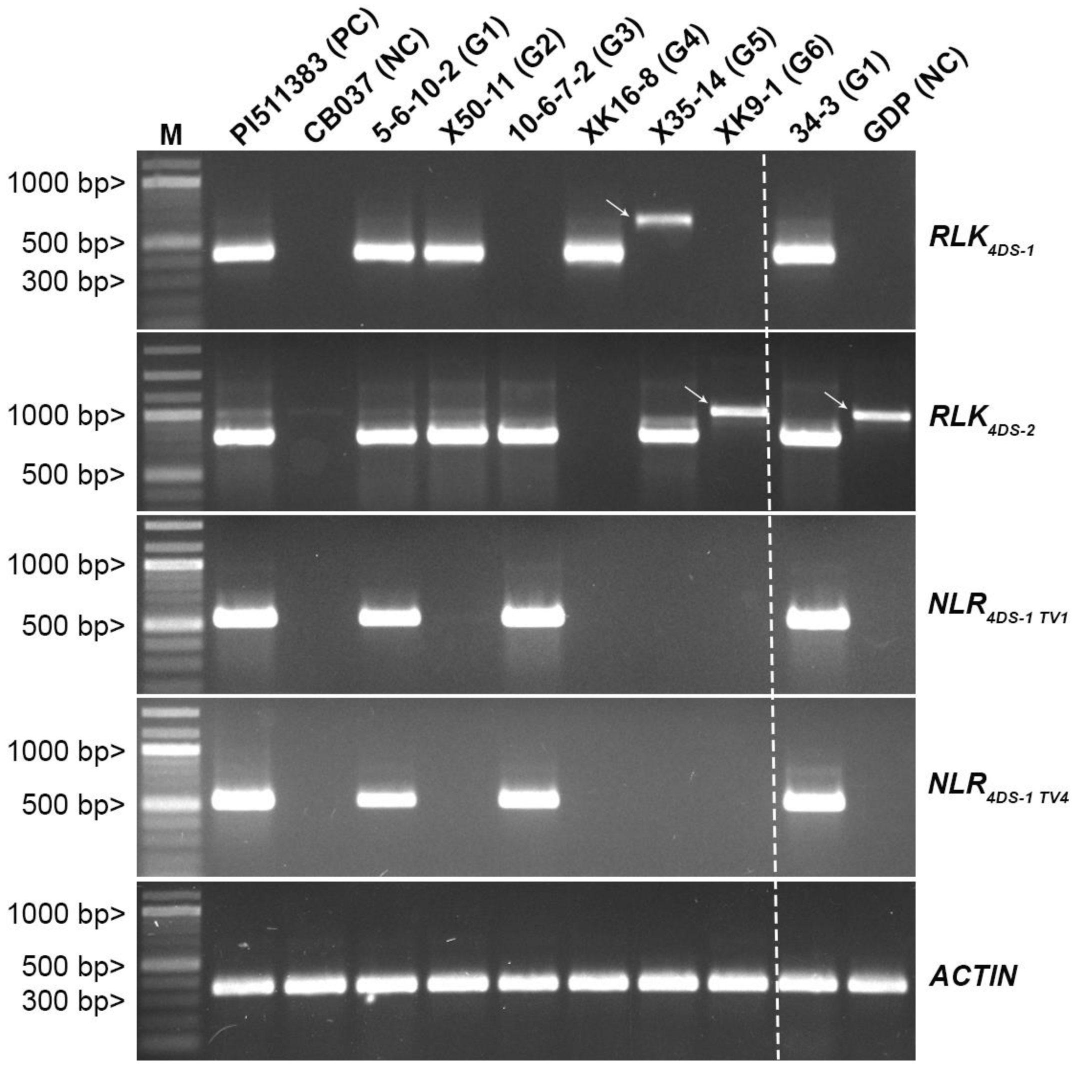


**Supplementary Fig. 4. Full-length transgene expression in transgenic wheat and barley.** Full-length transgene expression is illustrated for representative groups (G1 to G6 in parentheses). The positive control (PC) PI511383 and transformants G1 and G3 were resistant to *Puccinia striiformis* f. sp. *tritici*; all of the other transformants and the negative control (NC) were susceptible. Wheat (on the left) and barley are separated by a vertical dashed line. White arrows indicate either genomic amplification or non-specific amplification. Two rounds of RT-PCR were conducted for each target gene. For the first and second rounds of transgene amplification, the following primers were used: for $RLK_{4DS-1}$, P162/P199 and P193/P194; for $RLK_{4DS-2}$, P200/P188 and P203/P204; for $NLR_{4DS-1\ TV1}$, P189/P201 and P177/P205; and for $NLR_{4DS-1\ TV4}$, P202/P178 and P206/P207. *ACTIN*, with only one round of amplification with PCR primers P191 and P192, was included as the internal control. Other abbreviations include base pairs (bp), Golden Promise (GDP) barley, and molecular standard (M=2-Log DNA Ladder). Source data are provided as a Source Data file.

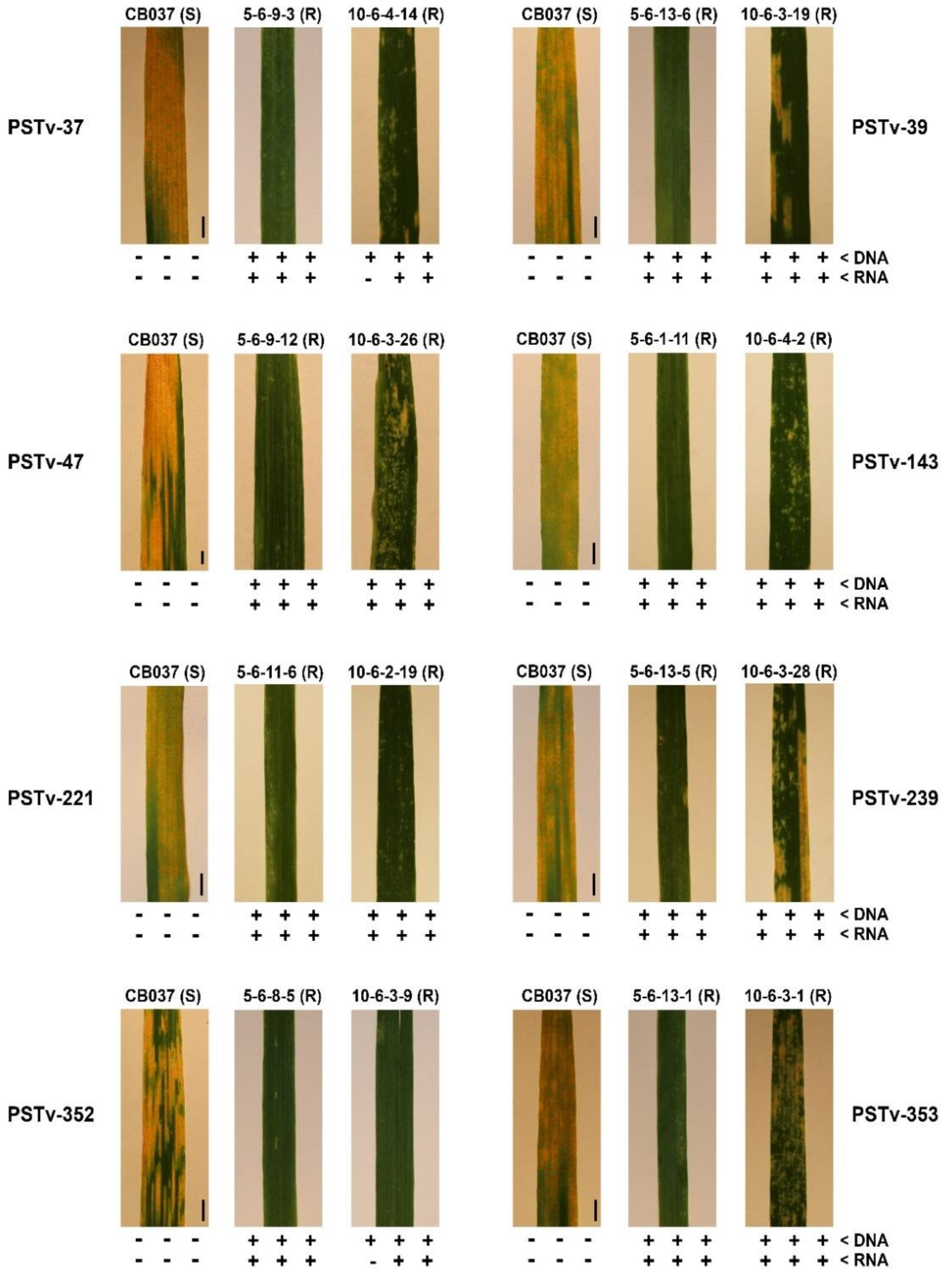


**Supplementary Fig. 5. The *YrAS2388R* locus confers resistance to multiple races of *Puccinia striiformis* f. sp. *tritici* in hexaploid wheat.** Susceptible hexaploid wheat CB037 was transformed with the intact PC1104. Transgenic $T_3$ wheat (all from the No. 5 and 10 $T_2$ subfamilies) was challenged with either one of seven *Pst* (*Puccinia striiformis* f. sp. *tritici*) races at the seedling stage or PSTv-47 at the adult plant stage. Plant responses (R=resistant; S=susceptible) to *Pst* are indicated in parentheses. Under each picture, PCR results illustrate positive (plus signs) or negative (minus signs) for DNA amplification (upper) and RNA expression (lower) of the three target genes: $RLK_{4DS\text{-}1}$ (left), $RLK_{4DS\text{-}2}$ (middle) and $NLR_{4DS\text{-}1}$ (right). RT-PCR is illustrated in Supplementary Fig. 4. Bar=1 cm. Source data are provided as a Source Data file.

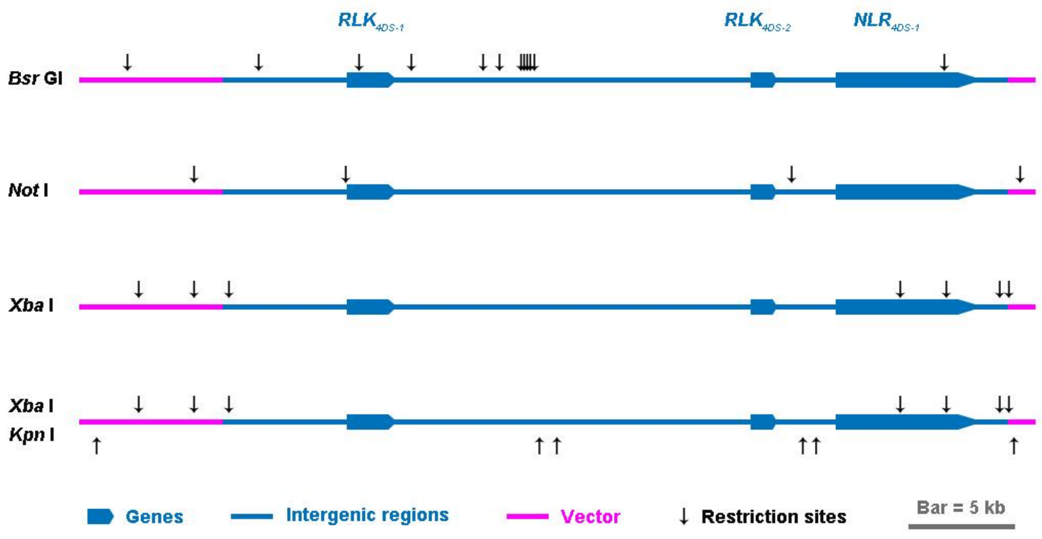


**Supplementary Fig. 6. Restriction enzyme-based digestion patterns of the fosmid PC1104.** Four restriction enzymes were selected that cleave either one or two target genes on the fosmid PC1104. The vector backbone is pCC1FOS (Epicentre Technologies Corp., Madison, WI, USA).

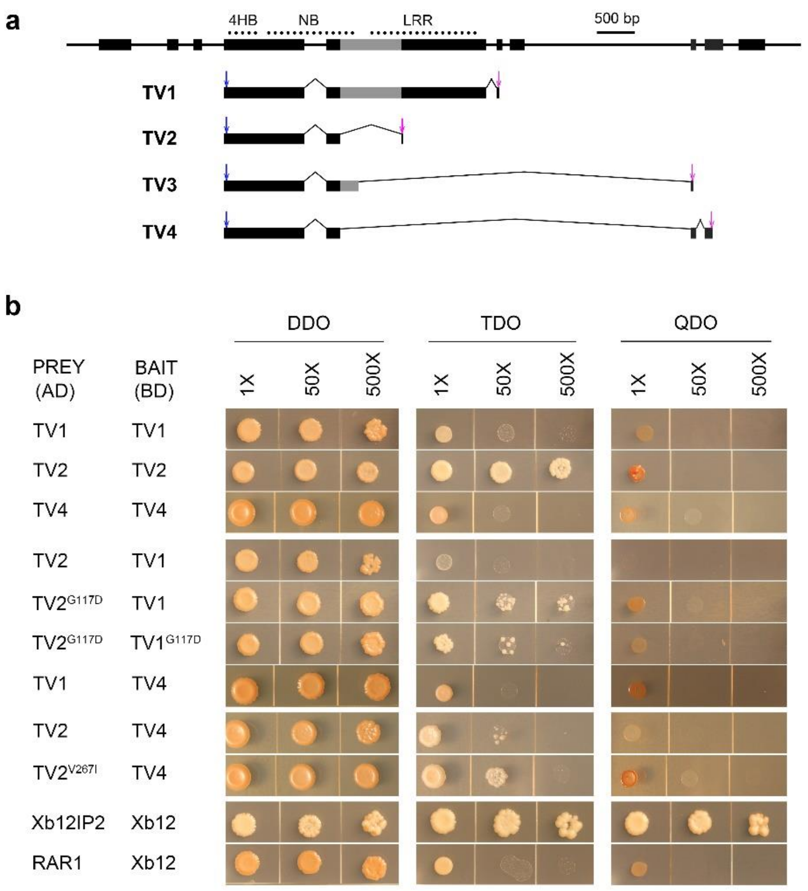


**Supplementary Fig. 7. NLR$_{4DS-1}$ variant proteins and their interactions as revealed by yeast two-hybrid.** (**a**) The coding regions of four NLR$_{4DS-1}$ variant proteins (TV1–TV4). Rectangles indicate exons. Abbreviations include four-helical bundle (4HB), nucleotide-binding (NB), leucine-rich repeat (LRR) and start (downwards arrows in blue) and stop (downwards arrows in magenta) codons. (**b**) Interactions of NLR$_{4DS-1}$ variant proteins (TV1–TV4) in the prey (AD) and bait (BD) vectors. Yeast colonies (1-, 50- and 500-fold dilutions) were grown on the double dropout medium (DDO: SD/-Trp/-Leu), the triple dropout medium (TDO: SD/-Trp/-Leu/-His) and the quadruple dropout medium (QDO: SD/-Trp/-Leu/-His/-Ade) (Clontech, Mountain View, CA, USA). Xb12 (PC332) and Xb12IP2 (PC324) were used as a positive control; Xb12 and RAR1 (PC322) were used as a negative control[6]. Source data of Supplementary Figure 7b are provided as a Source Data file.

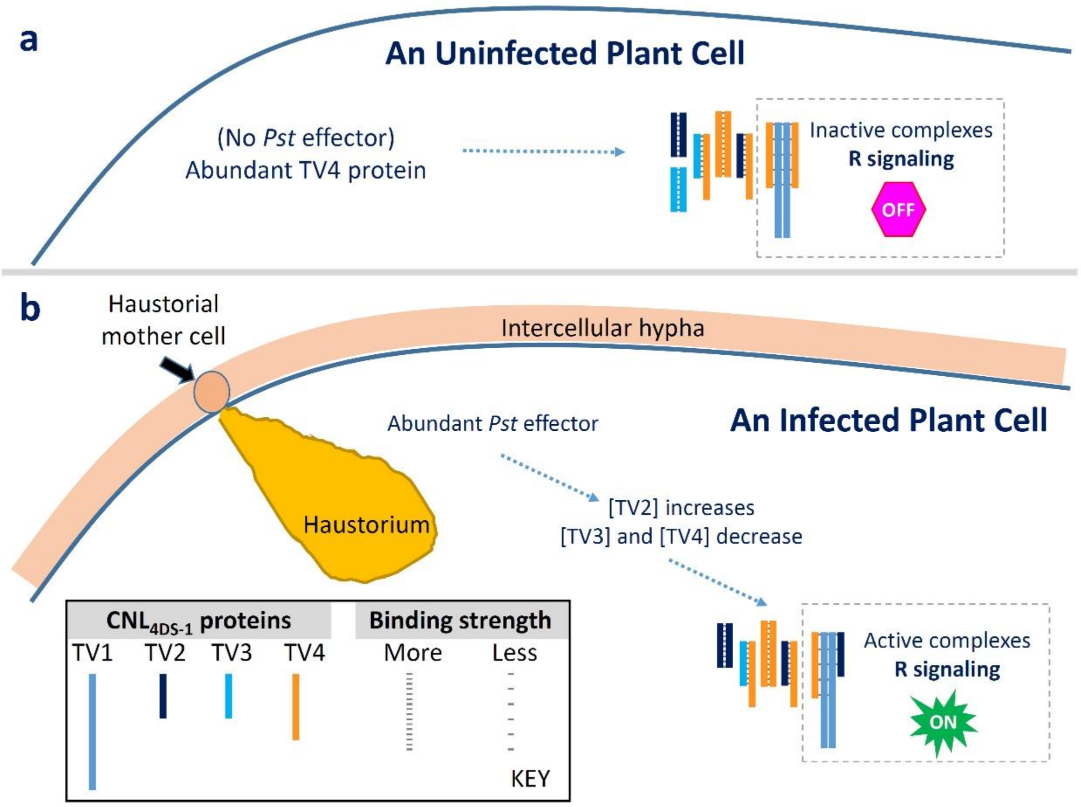


**Supplementary Fig. 8. A competition model for NLR$_{4DS-1}$ resistance to wheat stripe rust.** This model is proposed using the limited data available. The protein complex illustrates only the potential types of proteins involved; whether there are other proteins involved and whether they form a complex through dimerization or polymerization are not known. When there is no *Puccinia striiformis* f. sp. *tritici* (*Pst*) infection, TV4 is highly expressed, competing with TV2 for binding to TV1, forming an inactive TV1 complex, in which no defense responses are induced and plants save energy. When there is a *Pst* infection, an intermediate messenger affects *NLR$_{4DS-1}$* transcription, so TV2 is increased while TV4 and possibly TV3 is reduced, which permits the joining of TV2 to the complex for consequent defense signaling.

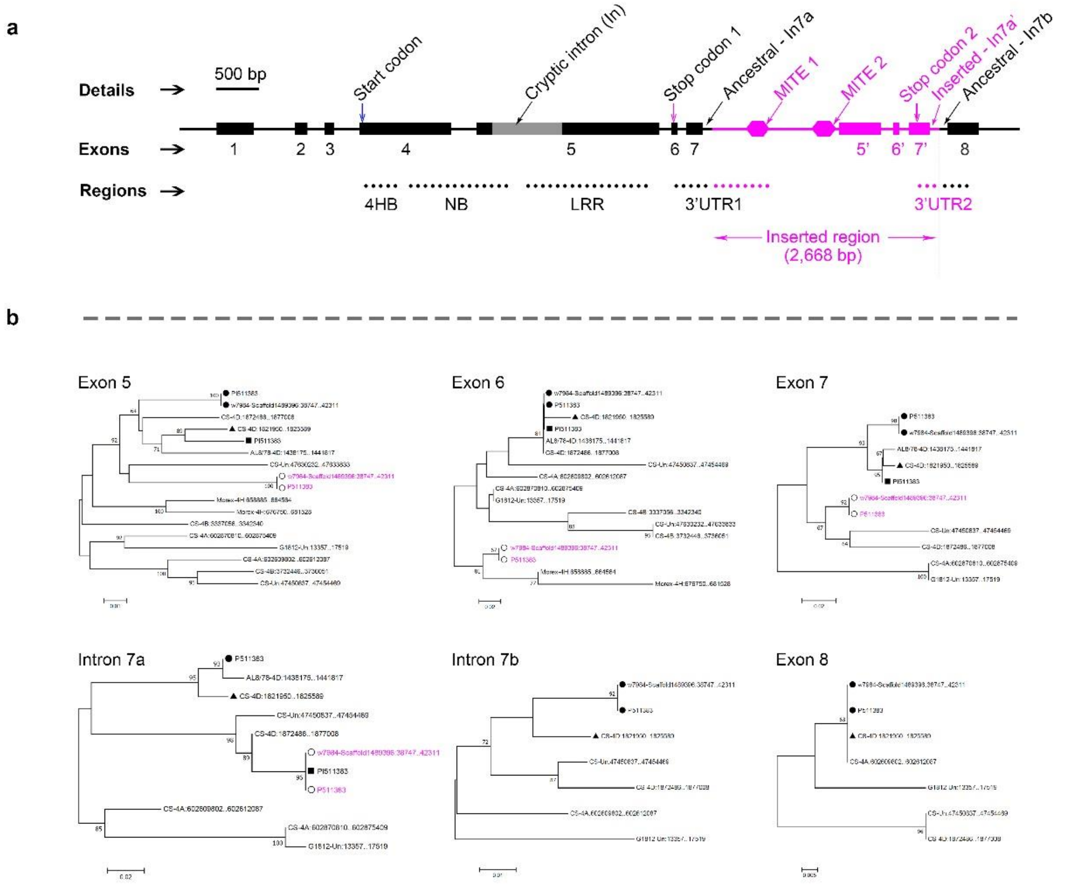


**Supplementary Fig. 9. Structure and duplicated fragments of the *NLR*$_{4DS-1}$ gene in PI511383.** (**a**) Genomic structure of the *NLR*$_{4DS-1}$ gene in PI511383. Regions of the conserved domains and two 3'UTRs are labelled; their approximate genomic locations were highlighted by dotted lines. A 2,668-bp fragment (magenta region), that carries exons 5' (partial), 6' and 7' of another paralog, was inserted into the ancestral 3'UTR of *NLR*$_{4DS-1}$. The current two 3'UTRs each contained both ancestral (black dots) and inserted (magenta dots) segments. Scale bar = 500 bp. (**b**) Phylogenetic trees of selected fragments (exons 5, 6, 7 and 8; introns 7a and 7b) in *NLR*$_{4DS-1}$ homologues in Triticeae. DNA and cDNA sequences of the following accessions were used: the barley Morex, the hexaploid wheat Chinese Spring (CS), and the wheat progenitor species: *Aegilops tauschii* accession AL8/78 and *Triticum urartu* accession G1812. The fragments of the presumed ancestral *Pst*-resistant *NLR*$_{4DS-1}$ are marked with solid circles, the *Pst*-susceptible *NLR*$_{4DS-1}$ in CS with solid triangles, and the *NLR*$_{4DS-2}$ in PI511383 with solid squares. The duplicated fragments (exons 5', 6', and 7'; intron 7a') of the *Pst*-resistant *NLR*$_{4DS-1}$ in PI511383 and W7984 are marked with open circles and magenta script. The program MEGA 7.0.26 [7] was used to align and construct a neighbor-joining tree with 1,000 bootstrap iterations. The distance scale bar indicates nucleotide differences per unit length. Bootstrap numbers (≥60) are shown at the respective nodes. For accessions with a whole genome sequences, genes are denoted as genotype (e.g. CS)-chromosome (or scaffold):location (start..end). Sequences were obtained from the IWGSC RefSeq v1.0 for CS[5], the ASM34745v1 for G1812, and the IBSC_PGSB_v2 for Morex (http://plants.ensembl.org) and the Aet v4.0 for AL8/78

(http://aegilops.wheat.ucdavis.edu/ATGSP/blast.php). Some scaffolds are associated with an unknown (Un) chromosome. Abbreviations include four-helical bundle (4HB), intron (In), leucine-rich repeat (LRR), two miniature inverted-repeat transposable elements (MITE), nucleotide-binding (NB) and start (downwards arrows in blue) and stop (downwards arrows in magenta) codons.

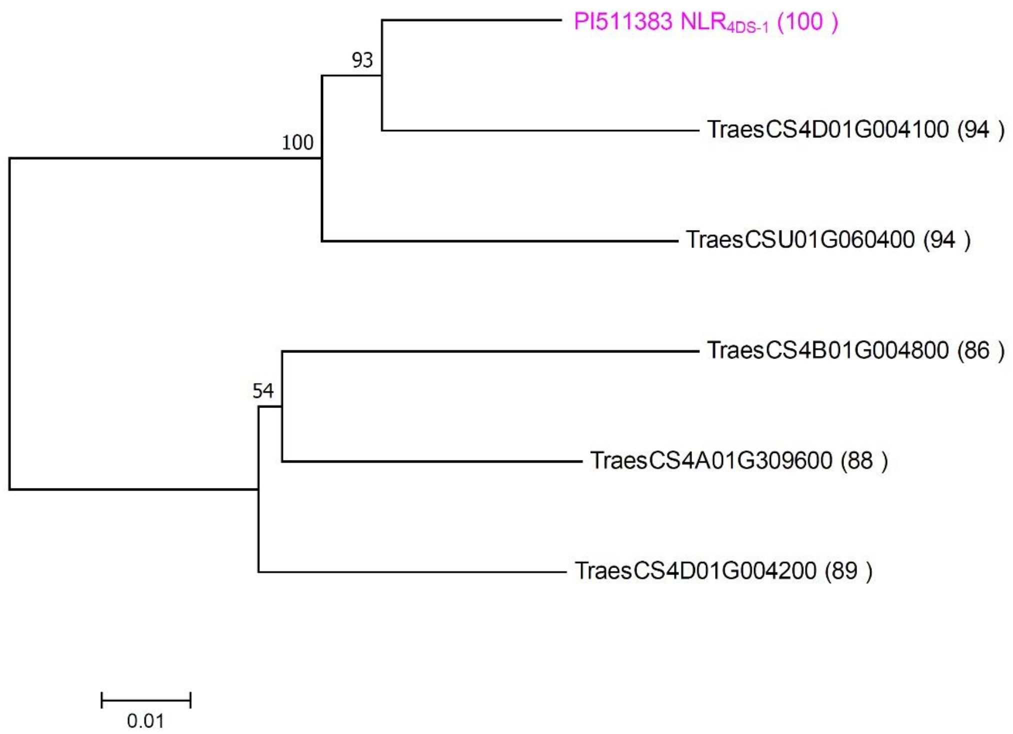


**Supplementary Fig. 10. Phylogenetic tree of five *NLR$_{4DS-1}$*-like genes in the common wheat Chinese Spring.** We used predicted cDNA of the transcriptionally active genes in Chinese Spring. MEGA 7.0.26 [7] was used to align and construct a neighbor-joining tree with 1,000 bootstrap iterations. The distance scale bar indicates nucleotide differences per unit length. Bootstrap numbers are shown at the respective nodes. The gene names are based on the annotation in the IWGSC RefSeq v1.0 for CS[5]. The *NLR$_{4DS-1}$* gene from PI511383 was included as a reference. The sequence percentage identities (vs. *NLR$_{4DS-1}$* in PI511383 in magenta color) are shown in the parentheses.

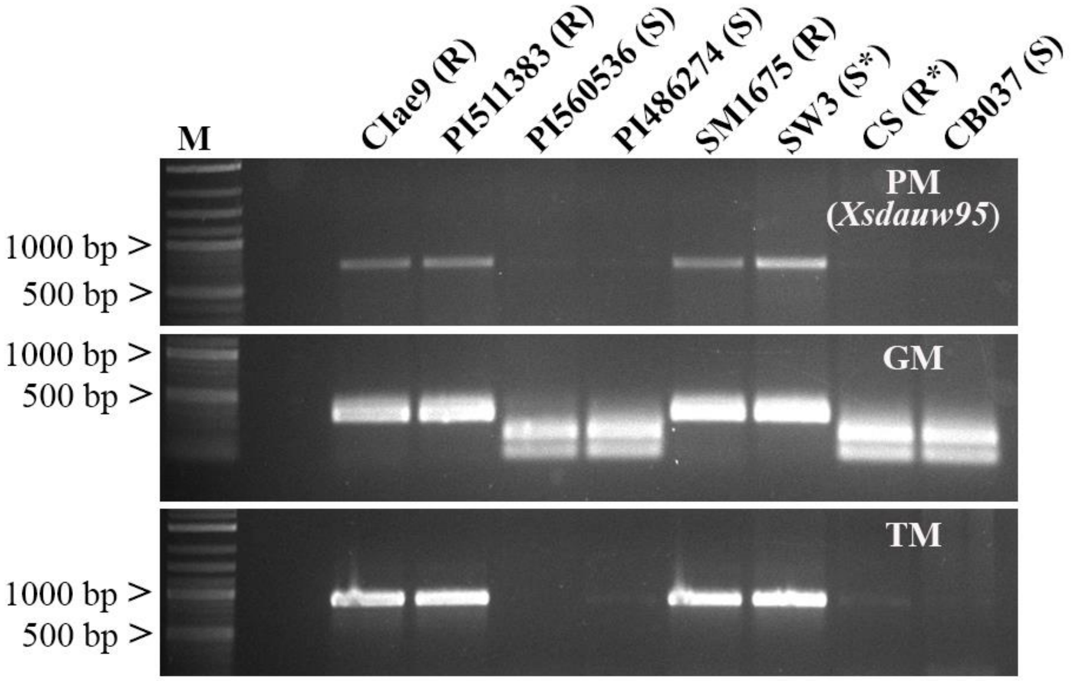


**Supplementary Fig. 11. PCR markers for *NLR*$_{4DS\text{-}1}$ from PI511383.** Promoter-derived (PM), gene-derived (GM) and terminator-derived (TM) markers were developed. The GM marker requires a *Hae*III enzyme digestion. For selected genotypes, plant responses (R=resistant; S=susceptible) to *Puccinia striiformis* f. sp. *tritici* (*Pst*) are indicated in parentheses. An asterisk indicates that the response to *Pst* is caused by other genes, i.e., *Yr18* in Chinese Spring[8] and an unknown suppressor in SW3. Source data are provided as a Source Data file. Abbreviations include base pairs (bp), Chinese Spring (CS), molecular standard (M=2-Log DNA Ladder) and Shumai 1675 (SM1675). Source data are provided as a Source Data file.

**Supplementary Table 1. Bulked segregant analysis of the *YrAS2388S* gene in 17 $F_2$ plants susceptible to *Puccinia striiformis* f. sp. *tritici***

| Markers | *YrAS2388S* | AT4D3406 | AT4D3410 | AT4D3411 | AT4D3412 | AT4D3413 | AT4D3417 | AT4D3418 | AT4D3419 | AT4D3420 | AT4D3423 | AT4D3424 | AT4D3425 | AT4D3426 | AT4D3427 | AT4D3429 | AT4D3430 | AT4D3431 | AT4D3432 | AT4D3433 | AT4D3435 | AT4D3438 |
|---|---|---|---|---|---|---|---|---|---|---|---|---|---|---|---|---|---|---|---|---|---|---|
| 4DS Location (cM)[a] | na[b] | 6.4 | 11.9 | 12.1 | 12.1 | 12.1 | 14.2 | 17.6 | 18.5 | 19.6 | 20.4 | 20.6 | 21.7 | 22.3 | 22.3 | 24.3 | 24.3 | 25.0 | 25.7 | 26.5 | 28.0 | 28.5 |
| *Pst*-susceptibel Lines | | | | | | | | | | | | | | | | | | | | | | |
| popB-L17 | B[c] | B | B | B | B | B | B | B | B | B | B | B | B | B | B | B | B | B | B | B | B | B |
| popB-L77 | B | B | B | B | B | B | B | B | B | B | B | B | B | B | B | B | B | B | B | B | B | B |
| popA-L10 | B | B | B | B | B | B | B | B | B | B | B | B | B | B | B | B | B | B | B | B | B | B |
| popA-L32 | B | B | B | B | B | B | B | B | B | B | B | B | B | B | B | B | B | B | B | B | B | B |
| popB-L80 | B | A[d] | A | A | A | A | A | A | A | A | A | A | A | A | A | A | A | A | A | A | A | A |
| popA-L60 | B | -[e] | A | A | A | A | A | A | A | A | A | A | A | A | A | A | A | A | A | A | A | A |
| popB-L34 | B | - | A | A | A | A | A | A | A | A | A | A | A | H | H | H | H | H | H | H | H | H |
| popA-L53 | B | - | H[f] | - | H | H | H | H | H | - | H | - | H | H | H | H | H | H | H | H | H | H |
| popA-L16 | B | B | H | H | H | H | H | H | H | - | H | - | H | H | H | H | H | H | H | H | H | H |
| popA-L34 | B | B | H | - | H | H | H | H | H | - | H | - | H | H | H | H | H | H | H | H | H | H |
| popA-L39 | B | B | B | B | B | B | B | B | B | - | H | - | H | H | H | H | H | H | H | H | H | H |
| popB-L84 | B | B | B | B | B | B | B | B | B | B | B | B | H | H | H | H | H | H | H | H | H | H |
| popA-L41 | B | B | B | B | B | B | B | B | B | B | B | B | B | H | H | H | H | H | H | H | H | H |
| popA-L49 | B | B | B | B | B | B | B | B | B | B | B | B | B | H | H | H | H | H | H | H | H | H |
| popB-L60 | B | B | B | B | B | B | B | B | B | B | B | B | B | B | B | B | B | H | H | H | H | H |
| popA-L61 | B | B | B | B | B | B | B | B | B | B | B | B | B | B | B | B | B | H | H | H | H | H |
| popB-L33 | B | B | B | B | B | B | B | B | B | B | B | B | B | B | B | B | B | B | H | H | H | H |

[a]Based on Luo *et al.* (2013)[1]; [b]not applicable; [c]the genotype of the *Pst*-susceptible parents; [d]the genotype of the *Pst*-resistant parents; [e]missing data; [f]a heterozygous genotype.

**Supplementary Table 2. PCR markers used in the current study**

| Markers | Types[a] | Forward Primer (5' to 3') | Reverse Primer (5' to 3') | Tm (°C)[b] | Enzymes[c] | Pop[d] |
|---|---|---|---|---|---|---|
| *Xsdauw2a* | InDel | TGCTTACTATATGAGCTGAGC | CTAGTACTCAAATTAACCGCC | 58 | - | A, B |
| *Xsdauw2b* | InDel | CGGCCTTCCTTTCTCAAAG | CTAGTACTCAAATTAACCGCC [e] | 59 | - | C |
| *Xsdauw3a* | CAPS | TATCATGCAGCTTTGCCTCG | CCACATCAAGATGACTGTACC | 59 | *Scr*FI | A, B, C |
| *Xsdauw8a* | InDel | GCTATTGCTGTCAAGGTCAG | CCAACATACTGAAACAGGGG | 59 | - | A, B |
| *Xsdauw36a* | InDel | GCGCATTTGGGTTAGAACTG | AGTGCATCAGCCAACCTTAG | 58 | - | A, B |
| *Xsdauw36b* | dCAPS | CTTCTCTGGCTCTGAGGTC | GCCTGTTACAGCAAGGTTAC | 60 | *Ava*II | C |
| *Xsdauw86* | DM | GACATATTTCTTGTAGTGGGTTC | TTTTTTTGTAGTGGGAGCAGC | 62 | - | A, B, C |
| *Xsdauw87* | DM | GTCGTTCCTAAAGTCTAGTAAC | CCAAGCCAAATTCGTCACTC | 59 | - | A, B, C |
| *Xsdauw88* | DM | CTACGTTCCCCAAAAATTAATTGG | GACTGGATGCGAAAGAATATAG | 62 | - | A, B, C |
| *Xsdauw89* | DM | AAATCCTCGCAACAGAGGTG | ATCTCGTTACTCCAAGGACC | 59 | - | A, B, C |
| *Xsdauw90* | DM | TTGCTCACTTGCTTTCTGCAG | CCAGTTAGCTATACATATCCC | 59 | - | A, B, C |
| *Xsdauw91* | CAPS | AAGCGGTGAACAGGAAGTGG | TTGGTTGAGGAGCTTGATGC | 59 | *Rsa*I | A, B |
| *Xsdauw92* | CAPS | TTATCTGCGACATACCCTCG | CAATGCTAGACACATGGGGC | 59 | *Rsa*I | A, B |
| *Xsdauw93* | DM | GAGCAGAACTGAAGAAGTACC | TGACAAAGCACTTGGGACAG | 59 | - | A, B |
| *Xsdauw94* | DM | CCACCTAAAGAAATCCCTGTC | TACTTCCACATCGAGCATGG | 59 | - | A, B |
| *Xsdauw95* | DM/InDel | TGTGTCATGTTTGGTCGATAGG | TCCTCCCTTGTAGCTTCACG | 59 | - | A, B, C |
| *Xsdauw96* | DM | CCACGCACAACAACCATAAG | TTTGGCCCAGTTATGACTCC | 59 | - | A, B, C |
| *Xsdauw97*[f] | (1st PCR) | ACGGTTTGCATGGGAAGATG | TTCTCAACCCTTGCAGCTTG | 60 | - | A, B |
| | CAPS (2nd PCR with nested | GACTACCAGAGAACATCCATG | TTCTCAACCCTTGCAGCTTG | 59 | *Nla*III | A, B |
| *Xwmc285* | SSR | TGTGGTTGTATTTGCGGTATGG | TTGTGGTGCTGAGTTAGCTTGT | 58 | - | A, B |
| *HTM1a* | DM | TGGTTCTTGGTGCAATCCTAACT | TCCTCTATAACTTGTGGGTCTAT | 58 | - | AeT collection |
| *HTM1b* | DM | GTGTCGGAGACTTTCAAGTC | CTATAGTTCCTTGCACCGTAG | 59 | - | Triticeae collection |

| | | | | | | |
|---|---|---|---|---|---|---|
| *HTM2a* | DM | CTTCGGCCATTGTGAGCG | ACTTTCTGTGAGGCGTCTGC | 58 | *Taq*I | AeT collection |
| *HTM2b* | DM | GCAACTACTAATCTCCTTCCTG | AACAAAACATGGACATGTGCAC | 58 | - | Triticeae collection |
| *HTM3a* (=*Xsdauw95*) | DM/InDel | TGTGTCATGTTTGGTCGATAGG | TCCTCCCTTGTAGCTTCACG | 59 | - | AeT collection |
| *HTM3b* | CAPS | ATATTCACCCTTCCCGTCTG | TTGGTTGAGGAGCTTGATGC | 59 | *Bsl*I | AeT collection |
| *HTM3c* | DM | TGTGCACTGTCTTTGCAAGC | GTGTAGTCCCAAACGACGTG | 60 | - | AeT collection |
| *HTM3d* (=*Xsdauw96*) | CAPS | AGGATTGGTTGAAGGAGACAC | ATCTCAAGCCTTGCAGCTTG | 59 | *Scr*FI | AeT collection |
| *HTM3e* | DM | GCACCGTCCTTCATCTCAGT | TGCTTTTCCCCGTATCCCTT | 60 | - | AeT collection |
| *HTM3f* | DM | CAAACAAAGGACAGGGCGTG | TTCATTTTCCTCTCCTGTGAC | 62 | - | Triticeae collection |
| *HTM3g* | CAPS | GCTTCCTTGACTTAAATTTCACCG | CCACATATCATCATTCAAGACG | 59 | *Hae*III | Triticeae collection |
| *PM* (=*Xsdauw95*) | DM/InDel | TGTGTCATGTTTGGTCGATAGG | TCCTCCCTTGTAGCTTCACG | 59 | - | MAS |
| *GM* (=*HTM3g*) | CAPS | GCTTCCTTGACTTAAATTTCACCG | CCACATATCATCATTCAAGACG | 59 | *Hae*III | MAS |
| *TM* | DM | AGATGAGAAGAAATGGCACGTG | CCAGTATACATCACTCTGATTCG | 60 | - | MAS |

[a]Cleavage amplification polymorphism sequence (CAPS), Degenerate cleavage amplification polymorphism sequence (dCAPS), Dominant marker (DM), Insertion/Deletion (InDel), Simple sequence repeats (SSR). [b]Annealing temperature. [c]Restriction enzymes (New England Biolabs) used to digest the PCR product; a minus sign indicates no enzyme was used. [d]Mapping populations (Pop). [e]Primers in gray color are identical to other primers in this table. [f]This marker requires two rounds of PCR; products of the second PCR were digested with *Nla*III.

**Supplementary Table 3. Haplotypes of the *YrAS2388* region in *Aegilops tauschii***

| Haplotypes | | Markers, location[a] & genotypes | | | Number of lines tested[b, c] | | |
|---|---|---|---|---|---|---|---|
| | | *RLK*$_{4DS-1}$ HTM1a +1441..+2326[a] | *RLK*$_{4DS-2}$ HTM2a -334..+669 | *NLR*$_{4DS-1}$ HTM3S[d] -2305..+3767 | *strangulata* | *tauschii* | Total |
| *Pst* resistant group | R1 | A | A | A[e] | 35 | 22 | 57 |
| | R2 | A | - | A | 2 | 0 | 2 |
| | R3 | - | - | A | 0 | 1 | 1 |
| *Pst* susceptible group | S1 | A | A | V[f] | 0 | 3 | 3 |
| | S2 | A | B | V | 0 | 8 | 8 |
| | S3 | - | A | V | 0 | 2 | 2 |
| | S4 | - | B | V | 0 | 29 | 29 |
| | S5 | A | - | V | 0 | 49 | 49 |
| | S6 | - | - | V | 0 | 8 | 8 |
| Sizes[g] | A | 886 bp | 536 bp | As[e] | na[h] | na | na |
| | Others | - | 192 bp | V[f] | | | |
| Number of lines (Subtotal and Total) | | | | | 37 | 122 | 159 |

[a]Locations are counted from “A” in the start codon (ATG) in the genomic allele (GenBank accession number MK288012). Here, we use two periods to separate the starting and ending nucleotides, in which a minus sign in the column title indicates a backward count from “A” and a plus sign in the column title indicates a forward count from “A”. Regular PCR was performed to genotype the haplotype markers (HTM). Alleles of CIae9 (which is *Pst*-resistant) are denoted by “A”; alleles that differ from those found in Clae9 are classified by other letters. A minus sign in the data area indicates a negative PCR reaction, possibly caused by a target region deletion or reduced primer efficiency. [b]Details about the tested lines are in Supplementary Data 3. [c]Subspecies of *Ae. tauschii*. [d]HTM3S represents a combination of HTM3a, HTM3b and HTM3c in the *NLR*$_{4DS-1}$ gene; specific genotypes are described in Supplementary Data 3. [e]The combination of all “A” genotypes (HTM3a to HTM3e). [f]A combination with at least one variant (V) genotype in either HTM3a, HTM3b, HTM3c, HTM3d or HTM3e. [g]Major diagnostic bands in base pairs (bp). [h]Not applicable (na).

**Supplementary Table 4. Plasmid constructs used in this study**

| Plasmid ID | Expression cassette[a] | GenBank Acc.[b] | Vector backbone |
|---|---|---|---|
| PC174 | *35S::BAR+35S::HYG* | - | pCAMBia1300 |
| PC1101 | *Ubi::NLR*$_{4DS\text{-}1\ TV1\text{-}cDNA1}$ | MK288012 | pCAMBia1300 |
| PC1102 | *Ubi::NLR*$_{4DS\text{-}1\ TV2\text{-}cDNA2}$ | MK288012 | pCAMBia1300 |
| PC1104 (F2-1) | *RLK*$_{4DS\text{-}1}$, *RLK*$_{4DS\text{-}2}$, *NLR*$_{4DS\text{-}1}$[c] | MK288012 | pCC1FOS |
| PC322 | *ADH1::AD:TaRAR1* | EF202841 | pGADT7 |
| PC324 | *ADH1::AD:wXb12IP2* | JX424313 | pGADT7 |
| PC332 | *ADH1::BD:wXb12* | JX424306 | pGBKT7 |
| PC1156 | *ADH1::AD:NLR*$_{4DS\text{-}1\ TV1}$ | MK288012 | pGADT7 |
| PC1157 | *ADH1::BD:NLR*$_{4DS\text{-}1\ TV1}$ | MK288012 | pGBKT7 |
| PC1158 | *ADH1::AD:NLR*$_{4DS\text{-}1\ TV2}$ | MK288012 | pGADT7 |
| PC1159 | *ADH1::BD:NLR*$_{4DS\text{-}1\ TV2}$ | MK288012 | pGBKT7 |
| PC1170 | *ADH1::AD:NLR*$_{4DS\text{-}1\ TV1\text{-}G117D}$[d] | - | pGADT7 |
| PC1171 | *ADH1::BD:NLR*$_{4DS\text{-}1\ TV1\text{-}G117D}$[d] | - | pGBKT7 |
| PC1172 | *ADH1::AD:NLR*$_{4DS\text{-}1\ TV2\text{-}G117D}$[d] | - | pGADT7 |
| PC1173 | *ADH1::BD:NLR*$_{4DS\text{-}1\ TV2\text{-}G117D}$[d] | - | pGBKT7 |
| PC1174 | *ADH1::AD:NLR*$_{4DS\text{-}1\ TV1\text{-}V267I}$[e] | - | pGADT7 |
| PC1175 | *ADH1::BD:NLR*$_{4DS\text{-}1\ TV1\text{-}V267I}$[e] | - | pGBKT7 |
| PC1176 | *ADH1::AD:NLR*$_{4DS\text{-}1\ TV2\text{-}V267I}$[e] | - | pGADT7 |
| PC1177 | *ADH1::BD:NLR*$_{4DS\text{-}1\ TV2\text{-}V267I}$[e] | - | pGBKT7 |
| PC1178 | *ADH1::AD:NLR*$_{4DS\text{-}1\ TV1\text{-}L421F}$[f] | - | pGADT7 |
| PC1179 | *ADH1::BD:NLR*$_{4DS\text{-}1\ TV1\text{-}L421F}$[f] | - | pGBKT7 |
| PC1184 | *ADH1::AD:NLR*$_{4DS\text{-}1\ TV4}$ | MK288012 | pGADT7 |
| PC1185 | *ADH1::BD:NLR*$_{4DS\text{-}1\ TV4}$ | MK288012 | pGBKT7 |

[a]*Ubi*, the maize ubiquitin promoter[9]; *ADH1*, a truncated promoter of the *alcohol dehydrogenase 1* gene from *Saccharomyces cerevisiae*[10]; *35S*, the CaMV *35S* promoter[11]; *BAR*: bialaphos resistance gene[12]; *HYG*: hygromycin resistance gene[13]. Expression cassettes are cDNA copies except as indicated with footnote "c." [b]GenBank accessions of the target genes; a minus sign=not applicable or not accessible. [c]Genomic DNA copy of the wheat gene. [d]Single-base mutation G350A caused the residue change G117D. [e]Single-base mutation G805A caused the residue change V267I. [f]Single-base mutation C1560T caused the residue change L421F.

**Supplementary Table 5. Haplotypes of the *YrAS2388* region in Triticeae**

| Haplotypes | | Markers, location & genotypes[a] | | | | Number of lines[b, c] | | | | |
|---|---|---|---|---|---|---|---|---|---|---|
| | | *RLK*$_{4DS-1}$ HTM1b +911..+2118 | *RLK*$_{4DS-2}$ HTM2b -1103..-449 | *NLR*$_{4DS-1}$ HTM3f +271..+1367 | *NLR*$_{4DS-1}$ HTM3g +1157..+1517 | Ae | Tt | Ta | Other | Total |
| AeT group | Ae1 | A | A | A | A | 36 | 0 | 0 | 0 | 36 |
| | Ae2 | - | A | A | A | 15 | 0 | 0 | 0 | 15 |
| | Ae3 | - | A | A | H[d] | 4 | 0 | 0 | 0 | 4 |
| | Ae4 | A | - | A | A | 2 | 0 | 0 | 0 | 2 |
| | Ae5 | A | A | - | A | 4 | 0 | 0 | 0 | 4 |
| | Ae6 | A | A | - | B | 4 | 0 | 0 | 0 | 4 |
| | Ae7 | - | A | - | B | 19 | 0 | 0 | 0 | 19 |
| | Ae8 | - | B | - | B | 4 | 0 | 0 | 0 | 4 |
| | Ae9 | A | - | - | A | 3 | 0 | 0 | 0 | 3 |
| | Ae10 | A | - | - | B | 9 | 0 | 0 | 0 | 9 |
| | Ae11 | - | - | - | A | 3 | 0 | 0 | 0 | 3 |
| Null group | Nu1 | - | - | - | B | 37 | 17 | 426 | 0 | 480 |
| | Nu2a | - | - | O[e] | - | 0 | 0 | 0 | 41 | 41 |
| | Nu2b | - | - | - | - | 0 | 124 | 35 | 91 | 250 |
| Sizes[f] | A | 1208 bp | 655 bp | 1097 bp | 361 bp | na | na | na | na | na |
| | B | na[g] | ≈1000 bp | O[e] | 240 bp[h] | | | | | |
| Number of lines (Subtotal and Total) | | | | | | 140 | 141 | 461 | 132 | 874 |

[a]'Locations' are described in Supplemental Table 2. Regular PCR was performed to genotype the haplotype markers (HTM). Alleles of the *Pst*-resistant CIae9 alleles are denoted by "A"; different alleles are marked by other letters. A minus sign in the data area indicates a negative PCR reaction, possibly caused by a low primer efficiency due to substantial variation in *RLK*$_{4DS-1}$, *RLK*$_{4DS-2}$ and *NLR*$_{4DS-1}$ genes. Sizes of PCR products for A and B type alleles are

indicated at the bottom of the table. [b]Details about tested lines are in Supplementary Data 6. [c]Ae=*Aegilops tauschii* and synthetic hexaploid wheat (SHW); Tt=*Triticum turgidum*; Ta=*Triticum aestivum* (common wheat); Other=*Ae. comosa* (23 lines), *Ae. longissima* (8 lines), *Ae. sharonensis* (38 lines), *Dasypyrum villosum* (5 lines), *Hordeum vulgare* (10 lines) and *T. monococcum* (48 lines). [d]Heterozygous for both A and B genotypes, which only occurred in SHW lines. [e]Other (O) non-$NLR_{4DS\text{-}1}$ type bands that are about 1,200 base pairs (bp). [f]Major diagnostic bands in bp. [g]Not applicable (na). [h]With complete digestion, the upper and lower bands are 240 bp and 121 bp, respectively.

**Supplementary Table 6. Wheat yield and stripe rust resistance in Sichuan**

| Tested Lines | Mg $ha^{-1}$ | Yield vs. Check (%)[a] | Stripe rust resistance[b] |
|---|---|---|---|
| Hongyumai 3 | 6.08 | + 16.32 ** | MR |
| Shumai 1675[c] | 5.80 | + 11.01 ** | R |
| Mianmai 902 | 5.76 | + 10.28 ** | R |
| Chuanmai 96 | 5.71 | + 9.27 ** | R |
| Chuanmai 83 | 5.67 | + 8.47 ** | R |
| Shumai 114 | 5.66 | + 8.25 ** | R |
| Chuanyu 31 | 5.59 | + 7.01 ** | MR |
| Chuannong 33 | 5.55 | + 6.28 ** | R |
| Yumai 2 | 5.06 | - 3.28 * | R |
| Zhongkemai 108 | 4.99 | - 4.46 ** | R |
| Chuanmai 1566 | 4.89 | - 6.46 ** | MR |
| Nanmai 988 | 4.57 | - 12.53 ** | MS |
| Xikemai 26 | 4.22 | - 19.29 ** | MR |
| Mianmai 367 | 5.23 | Check | S |

[a]Yield trials were performed by the Sichuan Seed Administration Station in seven experimental sites in 2016-2017 season, using a randomized block design with three replications. The plot size was 13.3 $m^2$. Mianmai 367 was used as a standard. A plus sign indicated yield increase and a minus sign indicated yield decrease. The Fisher's Least Significant Difference test was used to compare each cultivar to the standard. Yield differences are marked with one asterisk if $P<0.05$ and two asterisks if $P<0.01$.[b]Resistance at the adult stage was evaluated at three sites after inoculating with *Pst* races that were predominant in Sichuan. [c]Shumai 1675 is the only breeding line that has the *YrAS2388R* gene. Plants were either resistant (R, ITs=0-3), moderately resistant (MR, ITs=4-5), moderately susceptible (MS, ITs=6), or susceptible (S, ITs=7-9) to *Pst*.

**Supplementary Table 7. Plant materials used in the current study**

| ID or accession No.[a] | Notes on parentage or alternative names | Species (pedigree) | Genomes | References |
|---|---|---|---|---|
| AL8/78 | -[b] | *Aegilops tauschii*. | DD | [4] |
| AS87 | - | *Ae. tauschii* | DD | [14] |
| CIae9 | - | *Ae. tauschii* | DD | [14] |
| PI486274 | - | *Ae. tauschii* | DD | [14] |
| PI511383 | - | *Ae. tauschii* | DD | [14] |
| PI511384 | - | *Ae. tauschii* | DD | [14] |
| PI560536 | - | *Ae. tauschii* | DD | [14] |
| popA | PI511383/PI486274 | *Ae. tauschii* | DD | [14] |
| popB | CIae9/PI560536 | *Ae. tauschii* | DD | [14] |
| popC | PI511384/AS87 | *Ae. tauschii* | DD | [14, 15] |
| CItr13165 | Langdon | *Triticum turgidum* | AABB | [16] |
| SW3 | Langdon/CIae9 | Synthetic wheat[c] | AABBDD | [17] |
| SW58 | Langdon/AL8/78 | Synthetic wheat | AABBDD | [17] |
| Syn-SAU-93 | AS2382/PI511384 | Synthetic wheat | AABBDD | [16] |
| W7984 | Altar84/*Ae. tauschii* (219) | Synthetic wheat | AABBDD | [18] |
| AvS | Avocet (without *Yr28*) | *T. aestivum* | AABBDD | [19] |
| AvSYr28NIL | Avocet (with *Yr28*) | *T. aestivum* | AABBDD | [19] |
| Bobwhite | - | *T. aestivum* | AABBDD | [20] |
| CB037 | - | *T. aestivum* | AABBDD | [21] |
| CItr 14108 | Chinese Spring (CS) | *T. aestivum* | AABBDD | [22] |
| Shumai 1675 | - | *T. aestivum* | AABBDD | This study |
| PI343079 | Golden Promise | *Hordeum vulgare* | HH | [23] |

[a]Wheat cultivars/lines used for haplotype analysis and suppression of *Pst*-resistance are listed in Supplementary Data 3, 5 and 6. All *Ae. tauschii* accessions except for AL8/78 and AS87 were provided by Dr H.E. Bockelman at the USDA-ARS, Aberdeen, ID, USA. [b]A minus sign indicates no additional note. [c]Synthetic wheats are contemporary crosses of *Ae. tauschii* and *T. turgidum*, the ancestors of *T. aestivum*. SW3 and SW58 were from Dr. J.D. Faris and Dr. S.S. Xu at the USDA-ARS, Fargo, ND, USA.

## Supplementary Table 8. Other PCR primers used in this study

| Primer | Primer sequence (5' to 3') | Application |
|---|---|---|
| P160 | TGTGGCTAGGGATGAAACAC | Fosmid library screening |
| P161 | CATCATATGGTCCTTCCTCG | Fosmid library screening |
| P162 | GATGAAGATAGGGATGCCGG | Mutant detection and cDNA expression of $RLK_{4DS\text{-}1}$ |
| P163 | AGAACTTCTGTCTCAGCGCC | Mutant detection for $RLK_{4DS\text{-}1}$ |
| P164 | TTTCTGCTTCGGGGACTGTG | Mutant detection for $RLK_{4DS\text{-}1}$ |
| P165 | AACAGAAACAATTCACCATGGC | Mutant detection for $RLK_{4DS\text{-}1}$ |
| P166 | CTTCACATGTGCACATGTCC | Mutant detection for $RLK_{4DS\text{-}2}$ |
| P167 | ACACAGGTATGACACGCACC | Mutant detection for $RLK_{4DS\text{-}2}$ |
| P168 | TATTCATACAATAGCACACGCTC | Mutant detection for $RLK_{4DS\text{-}2}$ |
| P169 | TGTGTCATGTTTGGTCGATAGG | Mutant detection for $NLR_{4DS\text{-}1}$ (promoter region) |
| P170 | CCTCTTTAGCCATCTCGGTG | Mutant detection for $NLR_{4DS\text{-}1}$ (promoter region) |
| P171 | TGTGCACTGTCTTTGCAAGC | Mutant detection for $NLR_{4DS\text{-}1}$ |
| P172 | GTGTAGTCCCAAACGACGTG | Mutant detection for $NLR_{4DS\text{-}1}$ |
| P173 | GCATGATGTACGGCTTCTCA | Mutant detection for $NLR_{4DS\text{-}1}$ |
| P174 | GAGTGGAGACATTGGACGCT | Mutant detection for $NLR_{4DS\text{-}1}$ |
| P175 | GCACCGTCCTTCATCTCAGT | Mutant detection for $NLR_{4DS\text{-}1}$ |
| P176 | TGCTTTTCCCCGTATCCCTT | Mutant detection for $NLR_{4DS\text{-}1}$ |
| P177 | CTGTAGTTGAACTCGAATTGGG | Mutant detection and cDNA expression of $NLR_{4DS\text{-}1}$ |
| P178 | CCAGTATACATCACTCTGATTCG | Mutant detection and cDNA expression of $NLR_{4DS\text{-}1}$ |
| P181 | ttaatGCGGCCGCTAGTTCAAGCGTGAGCAAAC | cDNA cloning for $NLR_{4DS\text{-}1\ TV1}$ |
| P182 | ttataGGCGCGCCTCGTTCGGCTGGAGTTTCAG[a] | cDNA cloning for $NLR_{4DS\text{-}1\ TV1}$ |
| P183 | atttaGGCGCGCCATATGGTCCTTCCTCGTTCG[a] | cDNA cloning for $NLR_{4DS\text{-}1\ TV2}$ |
| P184 | AAGCACGGTCAACTTCCGTA | DNA intergration of *BAR* |
| P185 | GAAGTCCAGCTGCCAGAAAC | DNA intergration of *BAR* |
| P186 | TTTAGCCCTGCCTTCATACG[b] | cDNA intergration of $NLR_{4DS\text{-}1\ TV1\ \&\ TV2}$ |
| P187 | TTTAGGTTCTTCGCATACTCG | cDNA expression of $RLK_{4DS\text{-}2}$ |
| P188 | AAAATCACTTCCGGGCAAGC | cDNA expression of $RLK_{4DS\text{-}2}$ |
| P189 | TAGTTCAAGCGTGAGCAAACC | cDNA expression of $NLR_{4DS\text{-}1\ TV1\ \&\ TV2}$ |
| P190 | TCGTTCGGCTGGAGTTTCAG | cDNA intergration and cDNA expression of $NLR_{4DS\text{-}1\ TV1\ \&}$ |
| P191 | TATGCCAGCGGTCGAACAAC | DNA or cDNA expression of *ACTIN*[c] |
| P192 | GGAACAGCACCTCAGGRCAC | DNA or cDNA expression of *ACTIN*[c] |
| P193 | GTGTCGGAGACTTTCAAGTC | cDNA expression of $RLK_{4DS\text{-}1}$ |
| P194 | GATGTCGGCCCTGTGAGAA | cDNA expression of $RLK_{4DS\text{-}1}$ |
| P195 | CTTCGGCCATTGTGAGCG | cDNA expression of $RLK_{4DS\text{-}2}$ |
| P196 | ACTTTCTGTGAGGCGTCTGC | cDNA expression of $RLK_{4DS\text{-}2}$ |
| P197 | CAAGAAGGAGAAAACACGACG | cDNA expression of five transcript variants of $NLR_{4DS\text{-}1}$ |

| | | |
|---|---|---|
| P198 | ACTCCATTAGTTGCTTGCACT | cDNA expression of five transcript variants of $NLR_{4DS-1}$ |
| P199 | TAGAACAACATAGTTGGGTGC | cDNA expression of $RLK_{4DS-1}$ |
| P200 | TCTGCAAGAGCACCCATAGC | cDNA expression of $RLK_{4DS-2}$ |
| P201 | CCATGTTTCTTCACCAGCTG | cDNA expression of $NLR_{4DS-1}$ |
| P202 | ACTACTTGCGAGACAGCACG | cDNA expression of $NLR_{4DS-1}$ |
| P203 | GTCAAATAATACAGTCGGGGC | DNA intergration and cDNA expression of $RLK_{4DS-2}$ |
| P204 | TGAAGGTATGCAAGAGCTTTGCA | DNA intergration and cDNA expression of $RLK_{4DS-2}$ |
| P205 | ATGGCTGATGCTTTTCCCCG | cDNA expression of $NLR_{4DS-1}$ |
| P206 | GAAGCATGAAAGCCTTTCATCC | cDNA expression of $NLR_{4DS-1}$ |
| P207 | TCCATTAGTTGCTTGCACTGC | cDNA expression of $NLR_{4DS-1}$ |
| P208 | AGCGAGTGATATAGATGCGC | DNA intergration and cDNA expression of $RLK_{4DS-1}$ |
| P209 | TGCAAATGGCCAGAGTTCAC | DNA intergration and cDNA expression of $RLK_{4DS-1}$ |
| P213 | TGCCATTGGGGCAGATCTTC | DNA intergration of $NLR_{4DS-1}$ |
| P214 | TTCATTTTCCTCTCCTGTGAC | DNA intergration of $NLR_{4DS-1}$ |
| P215 | TCCATGTGGGTTGGTGAAGG | Real-time PCR for $NLR_{4DS-1\ TV1}$ |
| P216 | GTTGCAAATAAGTTGACTAGGG | Real-time PCR for $NLR_{4DS-1\ TV1}$ |
| P217 | TGCAAGAAGGAGAAAACACG | Real-time PCR for $NLR_{4DS-1\ TV2}$ |
| P218 | CAGTCACCATCAATATAGGCACA | Real-time PCR for $NLR_{4DS-1\ TV2}$ |
| P219 | TCCATGTGGGTTGGTGAAGG | Real-time PCR for $NLR_{4DS-1\ TV3}$ |
| P220 | CCTTTCGCCTAAACTCGTTC | Real-time PCR for $NLR_{4DS-1\ TV3}$ |
| P221 | TGCAAGAAGGAGAAAACACG | Real-time PCR for $NLR_{4DS-1\ TV3\ \&\ TV4}$ |
| P222 | CCTTTCGCCTAAACTCGTTC | Real-time PCR for $NLR_{4DS-1\ TV3\ \&\ TV4}$ |
| P223 | GCCATGTACGTCGCAATTCA | Real-time PCR for *ACTIN* |
| P224 | AGTCGAGAACGATACCAGTAGTACGA | Real-time PCR for *ACTIN* |

[a]To assist in cloning, *Not*I or *Asc*I were included in the primer. The restriction sites are highlighted by an underline. [b]This primer is based on the vector backbone sequence. [c]The *ACTIN* primers amplify both wheat, barley and *Aegilops tauschii*.

## Supplementary References


1. Luo M-C, *et al.* A 4-gigabase physical map unlocks the structure and evolution of the complex genome of *Aegilops tauschii*, the wheat D-genome progenitor. *Proceedings of the National Academy of Sciences* **110**, 7940-7945 (2013).

2. Ni F, *et al.* Wheat *Ms2* encodes for an orphan protein that confers male sterility in grass species. *Nature Communications* **8**, 15121 (2017).

3. Chapman JA, *et al.* A whole-genome shotgun approach for assembling and anchoring the hexaploid bread wheat genome. *Genome Biology* **16**, 26 (2015).

4. Luo M-C, *et al.* Genome sequence of the progenitor of the wheat D genome *Aegilops tauschii*. *Nature* **551**, 498-502 (2017).

5. IWGSC. Shifting the limits in wheat research and breeding using a fully annotated reference genome. *Science* **361**, eaar7191 (2018).

6. Cantu D, *et al.* Comparative analysis of protein-protein interactions in the defense response of rice and wheat. *BMC Genomics* **14**, 166 (2013).

7. Kumar S, Stecher G, Tamura K. MEGA7: Molecular evolutionary genetics analysis version 7.0 for bigger datasets. *Molecular Biology and Evolution* **33**, 1870-1874 (2016).

8. Krattinger SG, *et al.* A putative ABC transporter confers durable resistance to multiple fungal pathogens in wheat. *Science* **323**, 1360-1363 (2009).

9. Christensen AH, Sharrock RA, Quail PH. Maize polyubiquitin genes: structure, thermal perturbation of expression and transcript splicing, and promoter activity following transfer to protoplasts by electroporation. *Plant Molecular Biology* **18**, 675-689 (1992).

10. Louvet O, Doignon F, Crouze M. Stable dna-binding yeast vector allowing high-bait expression for use in the two-hybrid system. *BioTechniques* **23**, 816-820 (1997).

11. Kay R, Chan A, Daly M, Mcpherson J. Duplication of CaMV 35S promoter sequences creates a strong enhancer for plant genes. *Science* **236**, 1299-1302 (1987).

12. Thompson CJ, *et al.* Characterization of the herbicide-resistance gene bar from *Streptomyces hygroscopicus*. *The EMBO Journal* **6**, 2519-2523 (1987).

13. van den Elzen PJM, Townsend J, Lee KY, Bedbrook JR. A chimaeric hygromycin resistance gene as a selectable marker in plant cells. *Plant Molecular Biology* **5**, 299-302 (1985).

14. Liu M, *et al.* Stripe rust resistance in *Aegilops tauschii* germplasm. *Crop Science* **53**, 2014-2020

(2013).

15. Huang L, *et al.* Molecular tagging of a stripe rust resistance gene in *Aegilops tauschii*. *Euphytica* **179**, 313-318 (2011).

16. Zhang L-Q, *et al.* Frequent occurrence of unreduced gametes in *Triticum turgidum–Aegilops tauschii* hybrids. *Euphytica* **172**, 285-294 (2010).

17. Friesen TL, Xu SS, Harris MO. Stem rust, tan spot, stagonospora nodorum blotch, and hessian fly resistance in Langdon durum-*Aegilops tauschii* synthetic hexaploid wheat lines. *Crop Science* **48**, 1062-1070 (2008).

18. Sorrells ME, *et al.* Reconstruction of the synthetic W7984 × Opata M85 wheat reference population. *Genome* **54**, 875-882 (2011).

19. Wan A, Chen X. Virulence characterization of *Puccinia striiformis* f. sp. *tritici* using a new set of *Yr* single-gene line differentials in the United States in 2010. *Plant Disease* **98**, 1534-1542 (2014).

20. Warburton M, Skovmand B, Mujeeb-Kazi A. The molecular genetic characterization of the 'Bobwhite' bread wheat family using AFLPs and the effect of the T1BL.1RS translocation. *Theoretical and Applied Genetics* **104**, 868-873 (2002).

21. Peña PA, *et al.* Molecular and phenotypic characterization of transgenic wheat and sorghum events expressing the barley alanine aminotransferase. *Planta* **246**, 1097-1107 (2017).

22. Sears ER, Miller TE. The history of Chinese Spring wheat. *Cereal Research Communications* **13**, 261-263 (1985).

23. Thomas WTB, Powell W, Wood W. The chromosomal location of the dwarfing gene present in the spring barley variety Golden Promise. *Heredity* **53**, 177 (1984).
