# Supplementary material for: An ancestral NB-LRR with duplicated 3′UTRs confers stripe rust resistance in wheat and barley
Source: Nat Commun. 2019 Sep 6;10:4023. doi: 10.1038/s41467-019-11872-9 (PMC6731223; doi:10.1038/s41467-019-11872-9)
Supplement: Supplementary file 4 — Description of Additional Supplementary Files [file 41467_2019_11872_MOESM4_ESM.docx]

**Description of Additional Supplementary Files**

File Name: Supplementary Data 1 Virulence and host response to the tested races of *Puccinia striiformis* f. sp. *tritici* (*Pst*)
Description: ^a^Based on the WSU stripe rust web (https://striperust.wsu.edu/races/data/) and references. ^b^Seedling test on wild-type plants. ^c^Adult plant test on wild-type plants. ^d^Seedling test on T_3_ transgenic plants (all from the No. 5 and 10 T_2_ subfamilies). ^e^Adult plant test on T_3_ transgenic plants (all from the No. 5 and 10 T_2_ subfamilies). ^f^Wan, A., and Chen, X. (2014) Virulence characterization of *Puccinia striiformis* f. sp. *tritici* using a new set of *Yr* single-gene line differentials in the United States in 2010. Plant Disease 98: 1534-1542. ^g^Wan, A., Chen, X., and Yuen, J. (2016) Races of *Puccinia striiformis* f. sp. *tritici* in the United States in 2011 and 2012 and comparison with races in 2010. Plant Disease 100: 966-975. ^h^The predominant races in Latah county, Idaho (USA) in June, 2018 were PSTv-37 (70%) and PSTv-52 (20%).

File Name: Supplementary Data 2 DNA and protein differences in resistant versus susceptible alleles of the *NLR_4DS-1_* gene in *Aegilops tauschii* and common wheat
Description: ^a^Their physical locations are counted from “A” in the start codon (ATG) in the genomic allele (GenBank accession number MK736661); for an InDel, two periods were used to separate the starting and ending nucleotides. Only polymorphic bases that cause an amino acid change are shown. For each polymorphic location, those identical to the PI511383 resistance allele are shaded in blue, and polymorphisms are shaded in either orange or light green. ^b^Sequences of AL8/78, Chinese Spring and W7984 are from public databases, including AL8/78 pseudomolecules v4.0 (http://aegilops.wheat.ucdavis.edu/ATGSP/blast.php), *Triticum aestivum* (IWGSC) in EnsemlPlants (http://plants.ensembl.org/Triticum_aestivum/Tools/Blast?db=core), and w7984_Meraculous Scaffolds (http://www.cerealsdb.uk.net/cerealgenomics/CerealsDB/blast_WGS.php). ^c^The 6-bp Insertion (Ins)-Deletion (Del) in the *Pst*-susceptible genotypes causes one amino acid substitution (A96G) and two deletions (C97 and R98) in the *Pst*-susceptible alleles.

File Name: Supplementary Data 3 A complete list of *Aegilops tauschii* accessions used for haplotype analysis in the *YrAS2388* region
Description: ^a^The *Ae. tauschii* accessions were maintained by the Triticeae Research Institute, Sichuan Agricultural University, China. ^b^HT3S represents a combination of HTM3a, HTM3b, HTM3c, HTM3d and HTM3e in the *NLR_4DS-1_* gene. Alleles as in *Pst*-resistant PI511383 are denoted by “A”; different alleles are marked by other letters. An "A" in HT3S represents a combination of all “A” alleles in HTM3a, HTM3b, HTM3c, HTM3d and HTM3e; a "V" represents a combination with at least one non-A allele in HTM3a, HTM3b, HTM3c, HTM3d and HTM3e. ^c^This marker is likely located in the promoter of the *NLR_4DS-1_* gene. ^d^‘Locations’ of detected fragments are counted from “A” in the start codon (ATG) in the genomic allele (GenBank accession number MK288012). Here, we use two periods to separate the starting and ending nucleotides, in which a minus sign in the column title indicates a backward count from “A” and a plus sign in the column title indicates a forward count from “A”. ^e^Not determined. Note: A minus sign in the data area indicates a negative PCR amplification, which is caused by either low primer efficiency, a target region deletion, or a target region insertion. Note: Stripe rust reaction is based on Liu et al. (2013) Crop Science 53:2014-2020, and Liu et al. (2010) Genetic Resources and Crop Evolution 57:325-328.

File Name: Supplementary Data 4 Genotypes of *RLK_4DS-1_*, *RLK_4DS-2_* and *NLR_4DS-1_* in selected stripe rust-susceptible mutants of the *YrAS2388R*-positive wheat
Description: ^a^‘Locations’ of detected fragments are counted from “A” in the start codon (ATG) in the genomic allele (GenBank accession number MK288012). Here, we use two periods to separate the starting and ending nucleotides, in which a minus sign in the column title indicates a backward count from “A” and a plus sign in the column title indicates a forward count from “A”. *ACTIN* primers P191/P192 were used to confirm that all DNA samples had sufficient quantity and quality for PCR. Lines with *RLK_4DS-2_* were sequenced; no mutation was found in the PCR products except for the C(-221)T replacement identified in S19. Lines with the *NLR_4DS-1_* promoter were sequenced; no mutation was found in the PCR products. A plus sign in the data area denotes a positive PCR amplification. A minus sign in the data area denotes a negative PCR amplification, indicating a complete or partial deletion of the target region. Specific isoform(s) carrying the mutation are indicated in parentheses. ^b^Location of a nucleotide replacement was counted from the start codon forward (regular number) or backward (negative number). Location of a residue change was counted from the first methionine forward. "no" indicates no changes in DNA or protein. ^c^An additional mutation, G5702A, was also identified in L30, L59, L68 and L75, suggesting these four plants were derived from the same M_1_ plant.

File Name: Supplementary Data 5 Responses of the synthetic hexaploid wheat and their donor parental lines to *Puccinia striiformis* f. sp. *tritici*
Description: ^a^Inoculated 6 wk after planting. ^b^Inoculated 7 wk after planting. ^c^Based on Liu *et al*. (2013) Crop Science 53: 2014-2020. ^d^Grouped by accessions each with or without gray shading. ^e^Also based on Liu et al. (2010) Genetic Resources and Crop Evolution 57:325-328. ^f^F_1_ hybrids. ^g^Also based on the 2016-2017 test (inoculated 7 wk after planting) in Wenjiang, Sichuan, China. The stripe rust responses were recorded as resistant (R, ITs=0-3), moderate resistance (MR, ITs=4-5), moderate susceptibility (MS, IT=6) and susceptible (S, ITs=7-9). In the data area, a minus sign indicates missing data, a plus sign indicates the presence of the *YrAS2388R*, and 'nd' indicates that the presence of the *YrAS2388R* is not determined.

File Name: Supplementary Data 6 A complete list of Triticeae materials used for haplotype analysis in the *YrAS2388* region
Description: ^a^The *Aegilops tauschii* accessions were received from Dr. H.E. Bockelman at the USDA-ARS, Aberdeen, ID, USA. Synthetic hexaploid wheat (SHW) accessions were from Dr. T. Payne at the International Maize and Wheat Improvement Center, El Batán, Texcoco, México. ^b^Not tested (nt).
