# Supplementary material for: An ancestral NB-LRR with duplicated 3′UTRs confers stripe rust resistance in wheat and barley
Source: Nat Commun. 2019 Sep 6;10:4023. doi: 10.1038/s41467-019-11872-9 (PMC6731223; doi:10.1038/s41467-019-11872-9)
Supplement: Supplementary file 2 — Peer Review [file 41467_2019_11872_MOESM2_ESM.pdf]

Reviewers' comments:

Reviewer #1 (Remarks to the Author):

The manuscript by Zhang et al. presents a comprehensive data on the map-based cloning of the wheat stripe rust resistance gene YrAS2388 from the wheat D-genome progenitor, Aegilops tauschii.

Overall impression:
This is one of the most difficult manuscripts I have ever reviewed. The problem with this manuscript is the writing style, masses of supplementary data files and figures, several of which are difficult to follow. Many figures and tables instead of being stand along, actually cross reference other Tables, datafiles etc. This distracts the attention from the story and makes it incredibly difficult to identify a specific information. This manuscript in its current form does not read as a story I am afraid. Reading this manuscript is going to be a hard job for any reader. I feel this manuscript requires a major overhaul as a regular reader will not be able to follow the storyline. The story needs to be simplified, and any nonessential information removed, etc.

Major comments:
1. The gene cloned is another typical NLR, yet it appears to confer broad spectrum resistance. This is interesting, however the authors failed to demonstrate the underlying mechanism or come up with a plausible hypothesis of the mechanism.
2. YrAS2388 has 5 different alternative transcripts, only two (TV1 and TV2) of which appear to produce full-length NLR protein isoform. Transformation of susceptible wheat with the most prevalent isoform (TV1) failed to confer resistance to Pst. Whereas, no attempts were made to test the potential of other alternative transcripts for conferring Pst resistance. Therefore, even if a transcription of YrAS2388 is what determines resistance, the precise mechanism has not been revealed in this study.
3. It also appears that YrAS2388 is frequently suppressed when transferred to the hexaploid bread wheat. Therefore, this gene could probably be mainly utilised as a transgene (using a cisgenic approach).

Minor comments (these are numerous but I only list a few to exemplify the problems):
1. In the abstract, it would be useful to indicate that this gene is located on 4DS and that it encodes a typical NLR. No need to hide this fact. Then spell out a fairly unusual feature of this gene i.e. generation of 5 alternative transcripts. Although I am not sure how rare this feature is among the NLR genes. Is the occurrence of an alternative splicing is unique for YrAS2388? The following sentence in the abstract "at it has alternative splicing in conserved domains and two paralogous 3' untranslated regions" does not make sense to me and I feel it needs to be revised.
2. L42 in the Introduction: surely there are other regions affected by Pst apart from those two specifically mentioned? For example, what about Europe and the rest of USA, China?
3. L85-87, Suppl. Data 1 and Table 1: What 9 races out of 19 shown Suppl. Data 1 were used? Also, the data for CIae9 is not shown in this file. Suppl. Table 1 contains no data for PI511384 responses to Pst under HT.
4. L91: No data is presented for PI560536 in Table1 or Suppl. data 1 file.
5. L87-88: The sentence "The transgene YrAS2388 was later shown to be effective …" should probably removed, as this this is not an appropriate place (to early) to talk about this result.
6. L93: "the same gene" or different allelic variants?
7. L97: "'Langdon'-based hexaploid wheats" - what does this mean?
8. L100-101: should probably read "when introgressed into certain hexaploid wheat genotypes".
9. L102: replace "from" with "derived from".
10. L102-108, Figure S1: Not sure why this data is needed and what can be concluded from this. This data is not discussed or mentioned in the rest of the manuscript. Does this mean PI511384 contains an additional ASR gene or what?
11. L114-115: How is it possible to generate a linkage map with a cumulative map distance of 1,145 cM based on the genotyping of only 34 F2 plants?

12. L123-124: “In nature, the large 4DS QTL is inherited as a Mendelian trait and masks other minor QTL”. Not sure I understand the meaning of this sentence.
13. L127-128: “Thus we conclude that the 4DS QTL is the YrAS2388 gene, and that the gene is common in the Ae. tauschii ssp. strangulata.” This could only be hypothesized as no allelism test has been carried out to prove this.
14. L135: Please explain how the additional markers were generated.
15. L136: “4,205 popA plants”. What generation of plants of used, F2 or F3?
16. L140-141: “After assembling physical maps … we primarily analyzed the Xsdauw91-Xsdauw96 region”. It is not clear why this region was selected. There are appears to be no recombinants identified between CNL-4DS1 and MLK-4DS-1. Therefore, the latter could also be considered a candidate R gene.
17. L163-165: where do these new markers (HTM) come from, and what is their physical location in the YrAS2388 region?
18. L178: “after mutagenesis”. What type of mutagenesis was used here?
19. L198-203: not sure the analyses carried out here are legitimate. Every wheat genotype has different sensitivity to ems, and at concentration of 0.8% ems may induce very different rates of mutation in different genotypes. So that 1 mutation per each 30kb may only be achieved in that specific genotyped analysed in ref. 38.
20. L222-230: I do not understand these experiments at al. Restriction digestion should remove an entire wheat genomic DNA fragment rather than specific genes. This is assuming the diagram shown in Fig S7 is correct.
21. L230-231: what promoter was used to drive the expression of TV1 or TV2 in the transgenic plants? These experiments are is not detailed enough.
22. L273: “TV1 was expressed more in the seedling stages and less in the older plants”. I disagree with this statement. Only in L6 but not in L8 the TV1 expression seems to be low. Could it be an artefact of PCR? To firmly conclude on this, it is necessary to carry out a qRT-PCR analysis followed by assessing the data statistically to confirm/refute the above statement.
23. L275-276: Again, a potential difference in TV2 expression between the different samples/conditions needs to be verified by qRT-PCR.
24. L285-295: The yeast two-hybrid analysis data doesn't seem to provide any useful insight regarding the mechanisms underlying CNL-4DS-1 mediated resistance.
25. Figure 1: What isolate of Pst was used to generate Fig 1a? Were the plants naturally infected or in the lab? Fig 1c need to show direction of each genes (as in Figure S4). How was the sequence of MLK-4DS-1 gene determined as the two cosmids Fe-19 and Fa-13 do not overlap, and only one Fa-13 (according to the diagram) contains a partial sequence of this gene? A term “paralogous 3’UTRs” is confusing. Suggest indicating in Fig 1e the location of primers P197 and P198 used for the analyses of 5 alternative transcripts.
26. Figure S2: What crosses were used for this QTL analysis, and how many F2 plants were used?
27. Figure S3: what generation of plants (for each of the 3 crosses) were used?
28. Figure S4 is very confusing. Where does the marker O13 come from? The multicolour scheme used needs to be explained in the legend.
29. Figure S5: need to define the abbreviations used e.g. TV1, TV2, etc.
30. Many supplementary data file are too large and I am not sure whether all these are needed.

Reviewer #2 (Remarks to the Author):

Zhang et al., describe the mapping and cloning of the 4HB-NLR YrAS2388, which provides resistance to Puccinia when transformed into wheat and barley. The authors detail the characteristics of the gene and protein, with special interest given to the apparent splice variants, a cryptic intron and the four-helical bundle N-terminal domain.

Considering that the gene confers broad, durable resistance, it is very surprising that it is not in any hexaploid wheat lines. This could suggest that it is linked to very detrimental traits. Therefore, the

GMO route may be the only way to go.

Though the work is good, the writing is very long winded, they focus on the wrong parts of the story and the whole thing could be condensed significantly.

Notes to Authors:
It is described that there are DNA polymorphisms between YrAS2388-containing lines and non-containing lines. What are these polymorphisms? What do they suggest about the function of YrAS2388? This is interesting information that is left out. In addition to the GenBank accession for the functional YrAS2388 allele, they should submit the versions that they sequenced that appear to be non-functional for reference and include a figure showing where the mutations are in the gene.

Moreover, they frequently refer to organisms not having YrAS2388/CNL4DS-1, but this is not true, they just seem to have different versions that do not perform the resistance function you are assaying for. Therefore, it is improper to write this throughout the manuscript. They fix this in the paragraph starting at line 296, but should be fixed elsewhere.

Line 24-25: Describe what YrAS2388 is. i.e., an NLR. This also makes it unnecessary to refer to the NB and LRR domain in the abstract, so you can rewrite to focus just on the four-helical bundle that makes it interesting.

Lines 37-39: Weirdly small first paragraph

Line 46-48: Sentence is long and confusing, please rewrite.

Line 73: Undesirable is misspelt

Line 75: Add "rust" after "stripe"

Line 77: add "disease," after devastating

Line 92: Reword sentence

Mentions that the SW3 line, derived from the resistant A. tauschii line AvSYr28NIL, is not resistant, therefore YrAS2388 is suppressed in hexaploid genotypes. However, it is not described how SW3 was derived from AvSYr28NIL. Is it possible that there were any crosses or genetic events that could have removed YrAS2388 from the AvSYR28NIL D genome? To say that it is being suppressed, they would need to sequence it to confirm it is present.

Line 104: "Syn-SAU-93 only has adult plant resistance" and "Syn-SAU-93 is susceptible in the first leaf juvenile stage" are pretty much the same thing. Reword or remove one of these.

Is the revelation of CIae9 being PI511383 necessary for this story? Removing it would make these sections streamlined. If kept, this should be mentioned at the beginning of the results and then only one of them should be mentioned for the rest of the paper unless there are any other differences found.

Furthermore, if CIae9 = PI511383, and PI486274 ~= PI560536, then the two populations, popA and popB are almost exactly the same. They even removed the SNPs that were polymorphic between the two susceptible lines from the analysis. This makes the story a lot more confusing and could be condensed significantly!

Line 114: Should clarify that it was 34 F2 in total.

Lines 129-137: Explanation of mapping is too brief, saying you used the data to map does not explain what you did.

Line 230: Couldn't this suggest that neither TV1 or TV2 could be the important isoforms?

It is mentioned that CNL4DS-1 function is independent of RAR1, SGT1 and HSP90 due to a lack of interaction. However, this conclusion is flawed because evidence in R genes that do require these co-chaperones, e.g., the Mla locus, do not appear to interact when tested in their full-length forms but do so when only their LRR domains are expressed. If the authors wish to test this, they should repeat the interactions with a deletion series of TV1, especially sections of the LRR domain.

It would also make the figure much clearer if they showed a diagram of the gene regions used for Y2H, rather than just abbreviations (i.e., TV1, TV2)

Additionally, could it be possible that the mutant versions of TV2a actually sequester the non-mutant versions of TV1, and thus lower their activity? Therefore, acting as the suppressor you have implicated?

Line 386-389: Long convoluted sentence, please rewrite.

They should mention how their observations of YrAS2388 acting after 5dpi (line 105-108) suggest what the mechanism of action of the protein is.

Reviewer #3 (Remarks to the Author):

This work describes the isolation by positional cloning of a novel gene conferring stripe (yellow) rust resistance in wheat. Mutational analysis and transformation experiments confirmed the identity of the cloned gene. The gene YrAS2833 is derived from the wheat D genome ancestor species Ae. tauschii and encodes a canonical NLR immune receptor. Several splicing variants were identified and characterized for function and expression. Furthermore, the gene contains a duplicated 3' region. The gene is common in Ae. tauschii and synthetic wheat, but absent in all other bread wheat genotypes tested. This suggests that it was never incorporated (or was lost) in the hexaploid gene pool. No negative effect on yield and other studied agronomic traits was found.

The work is novel and of interest to the community. There are very few Yr resistance genes cloned and there is little information on their diversity and usefulness for breeding. The work presented in this manuscript adds highly valuable information and is of relevance for the characterization and practical use of genes in resistance breeding. However, the current manuscript is in many aspects unclear and lacks a detailed description of the work and the performed analysis.

Essential revisions
Alternative splicing:
This is an important part of the data but it is presented and described in insufficient detail. Much of lower part in Figure 1 needs clarification and expansion.

In Fig1d, the dotted description of the protein structure is unclear. Is the length of the LRR domain really very similar to the 4HB domain? This is hardly possible.
From where does the large red sequence in TV1 and TV2 derive from? It is impossible to correlate the information with the intro exon structure in Fig1d.
What does the labeling TV2a and TV2b mean in the figure. What is the difference between the two forms?

TV3: according to Fig1d there is a cryptic intro in E5. What is then the cryptic intron in TV3? Is it only partial, merged with which red exon? This is completely unclear.
In the figure legend of Figure 1 pseudogenes are mentioned. This is not described in the text and the reader wonders what else is lacking in the main text. Why are these genes pseudogenes? The text should allow the reader to follow the arguments of the authors.
Figure legend 1e lacks details (explanation of red segments, start and stop codons both with down arrows? There are also up arrows, what do they mean? What does M1 and M2 mean?

The whole Figure 1d and e needs to be redrawn. Possibly, and for the sake of clarity, this should be an own figure, providing much more detail and information.

Most importantly, the expression levels of the five different splice variants must be quantified by qRT-PCR. For none of the experiments and information in supplementary figures quantitative data are shown. Rather, unprecise description is provided, e.g. L273. It is possible that some variants are only present in very minor amounts and are simply due to inefficient splicing. This must be clarified and the data must be added. The model presented in L283 should then be based on better data.

L 109 ff: the development of populations is not clear and the reader has to go to Materials and Methods to find out what was done. The basic information on population development must be given here.

L. 140: Give explanation why "we primarily analyzed the … region". According to the physical map in Fig1c Xsdauw96 is a sequence located in the candidate gene (is this an intragenic recombinant, similar to Huang et al. Genetics, 2003, http://www.genetics.org/content/164/2/655.short; cloning of Lr21 from Ae. tauschii?). The description and logic of the presentation needs more detail and clarify. Is the gap in the physical map of the fosmids a problem, as the first flanking marker not located in the CNL candidate gene is on the right of the gap?

The description of the mutants is poor. Where does the reader find the information on the mutations (affected amino acids)? The main text refers to Supp data 5, but there the text refers to Supp table 1, which does not contain the information. The whole part has to be rewritten and clarified.

L211: Transgenic families 5 and 10 are mentioned. But they are not introduced and not described. The description of the transgenic validation experiment is basically missing.

As already stated above for Fig1e, the description of alternative splicing lacks detail and clarity.

L381: these data mentioned in the discussion should be presented in detail in the results section.

The methods section (starting at L585) needs expansion and clarification. What sequences were used for which genes, transformations? Bilaphos should be bialaphos? The selection plasmids are not described at all, there are no references, etc.

Additional points / at least partially essential:
The gene YrAS2833 was suppressed in many hexaploid wheat genotypes. This suppression effect is reminiscent of the Pm8 suppression by Pm3 homoeologs (Hurni et al. 2014). As the authors have identified homoeologs/homologs of YrAS2833 in wheat (see L381, L695) mechanistic studies using the transient N. benthamiana could clarify the molecular basis of suppression. The identification of the suppressor gene would be of great value for breeders so they could avoid introgression of the gene into suppressive germplasm. As the manuscript puts a lot of emphasis on breeding applications, a diagnostic marker for an active suppressor gene would be highly desirable.

L. 100: The homoeologs should be introduced here and presented as candidate suppressor genes.

Furthermore, the origin of the suppressing hexaploid lines should be presented and possible progenitors of the suppressor should be identified. This part of the manuscript is too short and lacks details.

Supp Fig2: Please indicate the position of YrAS2833.

Figure Legend Supp Figure 4: What do the colors mean? The whole figure needs more clarity (what is the correlation with Figure 1?) and description.

L 198: Does not fit in main text. It is sufficient to mention in the main text that EMS mutagenesis was used.

L264: Of, the… : needs rephrasing

L265: have … identity in the conserved region: the text needs clarification. Clearly, Fig1e does not give sufficient information to be able to follow the text.

The final paragraph of the results starting L309 needs language editing.

L366: Needs clarification.

REVIEWERS'S COMMENTS IN GREEN

OUR RESPONSES IN BLUE

I. Reviewer #1 (Remarks to the Author):

Overall impression:

This is one of the most difficult manuscripts I have ever reviewed. The problem with this manuscript is the writing style, masses of supplementary data files and figures, several of which are difficult to follow. Many figures and tables instead of being stand along, actually cross reference other Tables, datafiles etc. This distracts the attention from the story and makes it incredibly difficult to identify a specific information. This manuscript in its current form does not read as a story I am afraid. Reading this manuscript is going to be a hard job for any reader. I feel this manuscript requires a major overhaul as a regular reader will not be able to follow the storyline. The story needs to be simplified, and any nonessential information removed, etc.

Response: Thank you for your overall comment. We apologize for writing confusing text. We have tried to present a clearer narrative and to have removed nonessential information. Specific changes are indicated below.

Major comments:

I-1. The gene cloned is another typical NLR, yet it appears to confer broad spectrum resistance. This is interesting, however the authors failed to demonstrate the underlying mechanism or come up with a plausible hypothesis of the mechanism.

Response: There are hundreds of *NLR* genes in each major crop species. How a *NLR* gene might confer broad spectrum resistance is unknown. However, in response to this comment, we have included a plausible model that fits all our current data. The following description was added to the result and discussion sections.

"We hypothesize that TV1 plays a major role in the induction of stripe rust resistance, TV2 acts as a positive co-factor, and TV4 (or possibly TV3) act either as negative regulators when its expression is high or as positive regulators when its expression is low (Figure S8)."

"As a typical *NLR* gene, we hypothesized that the NLR_{4DS-1} proteins change their state via a competition model (Figure S8). The full-length TV1 protein plays a central role in signal transduction, but it requires other variant proteins (TV2 and TV4) for a proper conformation, which together form an active TV1 complex for defense signaling. When there is no *Pst* infection, TV4 is highly expressed, competing with TV2 for binding to TV1, forming an inactive TV1 complex, in which no defense responses are induced and plants save energy. When there is a *Pst* infection, an intermediate messenger affects the NLR_{4DS-1} transcription: TV2 is increased and TV4 and possibly TV3 is reduced, which permits the joining of TV2 to the complex, and consequent defense signaling."

"Figure S8 | A competition model for NLR_{4DS-1} resistance to wheat stripe rust. This model is proposed using a limited known data. The protein complex illustrates only the potential types of proteins involved; however, whether there are other proteins involved and whether they form the complex through

dimerization or polymerization are not experimentally documented. When there is no *Pst* infection, TV4 is highly expressed and competes with TV2 for binding to TV1, forming an inactive TV1 complex, in which no defense responses are induced and plants save energy. When there is a *Pst* infection, an intermediate messenger results in the reduction of TV4 and possibly TV3 transcription, which permits the joining of TV2 to the TV1-TV4 complex, thereby forming an active TV1 complex for defense signaling."

Finally, we would like to provide a brief justification for publication in Nature Communications without an elucidated mechanism. First, partly because of the importance of wheat in the human diet, the cloning of the *YrAS2388R* gene is an accomplishment. While this is not the first report of alternative splicing in an NLR, as far as we know, this is the first report of duplicated 3'UTRs in a rust resistance gene. Indeed, a number of groups worldwide have been trying to clone this gene for years. However, cloning has been extremely difficult; it took us nearly 10 years to finish the cloning part. Now we know the gene. The next logical step is to elucidate the mechanism of action. Because of its complex transcription, we believe that it will be another slow process, possibly another 10 years, to fully understand the mechanism of action.

I-2. *YrAS2388* has 5 different alternative transcripts, only two (TV1 and TV2) of which appear to produce full-length NLR protein isoform. Transformation of susceptible wheat with the most prevalent isoform (TV1) failed to confer resistance to *Pst*. Whereas, no attempts were made to test the potential of other alternative transcripts for conferring Pst resistance. Therefore, even if a transcription of *YrAS2388* is what determines resistance, the precise mechanism has not been revealed in this study.

Response: We appreciate the comment. But first, we want to clarify that only TV1 is a full-length NLR protein. The other three variants are incomplete proteins that lack the LRR domain. We transformed TV1 and TV2 cDNAs individually into susceptible wheat, and neither conferred disease resistance. Although this was unexpected at the time, it indicated that the function or mechanism of the *NLR*$_{4DS-1}$ gene is complicated. Certainly, we could test TV3 and TV4. However, we haven't done this for the following reasons. **First**, we have two types of mutations, S394N and Q557* (Supplementary data 5), that occurred only in the TV1 transcripts, which suggests that TV1 plays a central role in defense signaling. **Second**, TV3, similar to TV2, is not highly expressed. While TV4 is relatively highly expressed, it appears to be negative regulator when its expression is high. **Third,** overexpressing the cDNA of a classic *R* gene often does not confer the expected resistance; although this is widely known in the research community, few reports have been made about these important negative results. **Fourth**, as far as we know, only 13 resistance genes to a wheat rust have been cloned, including *Lr1* (Cloutier et al. 2007), *Lr10* (Feuillet et al. 2003), *Lr21* (Huang et al. 2003), *Lr34* (Krattinger et al. 2016; Rinaldo et al. 2017; Risk et al. 2013; Schnippenkoetter et al. 2017), *Lr67* (Moore et al. 2015), *Yr15* (Klymiuk et al. 2018), *Yr36* (Fu et al. 2009), *Sr13* (Simons et al. 2011), *Sr21* (Chen et al. 2018), *Sr22* (Steuernagel et al. 2016), *Sr33* (Periyannan et al. 2013), *Sr35* (Saintenac et al. 2013), and *Sr50* (Mago et al. 2015). All these studies focused on gene validation using genomic alleles rather than using cDNAs. **Fifth**, wheat transformation is not trivial, and we would like to better understand the mechanism of resistance before we proceed with a transformation strategy further. We believe that the qRT-PCR that we have added to the manuscript help in this regard.

Similar to our responses in I-1, we proposed that "The full-length TV1 protein plays a central role in signal transduction, but it requires other variant proteins (TV2 and TV4) for a proper conformation, which together form an active TV1 complex for defense signaling."

I-3. It also appears that *YrAS2388* is frequently suppressed when transferred to the hexaploid bread wheat. Therefore, this gene could probably be mainly utilised as a transgene (using a cisgenic approach).

Response: We somewhat agree. In the original manuscript, we stated… "Thus cisgenic *YrAS2388* is an example where a cisgenic approach can provide an advantage to consumers in comparison to traditional breeding." We also added the following sentence to the discussion. "Regardless, because the transgene $NLR_{4DS\text{-}1}$ induces effective *Pst* resistance in hexaploid wheat, we predict that sustainable *Pst* resistance can be achieved with either a cisgenic strategy with $NLR_{4DS\text{-}1}$ and/or a conventional strategy that combine both the incorporation of a resistant $NLR_{4DS\text{-}1}$ and the avoidance of the apparently linked latent suppressor(s) from *Ae. tauschii*."

However, in response to the reviewer comment, here we give a lengthier response to why utilization of *YrAS2388* may not be limited to a cisgenic approach. Most importantly, at this point, we have no idea how the suppression occurs. In a synthetic hexaploid wheat, the suppressor itself is either from the diploid parent (= D genome with the *YrAS2388R* gene, e.g. AS2388, CIae9, or PI511383), from the tetraploid parent (= AB genomes, e.g. the durum wheat 'Langdon'), or from their interaction.

1) If the suppressor is in the D genome, it is inactive since *YrAS2388R* confers strong resistance to *Pst* in the diploid genetic background. However, this D-genome suppressor may become active when in contact with A and B genomes from the durum partner. If this is the case, there are two approaches to use the *YrAS2388R* gene in a hexaploid genetic background. First, we can use this gene as a cisgenic, as suggested by the reviewer. This could be done by either traditional transformation or genome editing. But secondly, we could introgress the *YrAS2388R* region through repeated backcrosses (e.g. BC_6F_1) and start to identify recombinants of the *YrAS2388R* region in the BC_6F_2 and BC_6F_3 generations. Only the recombinant lines that carry a short fragment (e.g. 50 kb) of the *YrAS2388R* region from the diploid donor would be maintained for breeding.
2) If the suppressor is in the A or B genomes, we can use mutagenesis to inactivate the suppressor. For example, we have a synthetic hexaploid wheat with the *YrAS2388R* gene, but it is only moderately resistant or susceptible to *Pst*. We postulate that the most effective way to annul the suppressor is to mutagenize the synthetic hexaploid lines and to identify mutants with enhanced *Pst* resistance. The lines that stably inherit the enhanced *Pst* resistance can be used in breeding programs. The mutagenesis approach can also be used to knock out the D-genome suppressor. In the original manuscript, we also discussed the mutagenesis approach: "Nonetheless, when *YrAS2388R* is suppressed in a specific hexaploid wheat such as SW3, *Pst*-resistance levels might be increased by disrupting the unknown suppressor, as was previously done by inactivating a suppressor of stem rust resistance."

In the future, when the suppressor is cloned, other approaches could be developed for using the *YrAS2388R* gene.

Minor comments (these are numerous but I only list a few to exemplify the problems):

Response: We have tried to check the entire manuscript thoroughly.

I-1. 1) In the abstract, it would be useful to indicate that this gene is located on 4DS and that it encodes a typical NLR. No need to hide this fact. 2) Then spell out a fairly unusual feature of this gene i.e. generation of 5 alternative transcripts. Although I am not sure how rare this feature is among the NLR genes. Is the occurrence of an alternative splicing is unique for *YrAS2388*? The following sentence in the abstract "at it has alternative splicing in conserved domains and two paralogous 3' untranslated regions" does not make sense to me and I feel it needs to be revised.

Response : 1) Addressed as: "… is the source of the *YrAS2388*-resistant allele (*YrAS2388R*) that is **on chromosome arm 4DS** …. *YrAS2388R* encodes a nucleotide oligomerization domain **(NOD)-like receptor (NLR)** with a N-terminal four-helix bundle (4HB which was previously classified as a coiled-coil)."

Response: 2) We apologize for confusion by using paralogous 3'UTRs. We now changed it to duplicated 3'UTRs. As far as we know, this is the first report of duplicated 3'UTRs in a rust resistance gene. To streamline the narrative, we now state that "Unlike the *YrAS2388*-susceptible allele (*YrAS2388S*), **the resistant allele *YrAS2388R* evolved with duplicated 3' untranslated regions (3'UTR1 and 3'UTR1); each is associated with alternative splicing in the nucleotide-binding domain: two with 3'UTR1 and three with 3'UTR2**."

I-2. L42 in the Introduction: surely there are other regions affected by Pst apart from those two specifically mentioned? For example, what about Europe and the rest of USA, China?

Response: We replaced "such as the Pacific Northwest in the U.S. and the Sichuan basin in China" WITH "**including major wheat-producing countries such as Australia, Canada, China, France, India, United States, and many others**"

I-3. L85-87, Suppl. Data 1 and Table 1:1) What 9 races out of 19 shown Suppl. Data 1 were used? 2) Also, the data for CIae9 is not shown in this file. 3) Suppl. Table 1 contains no data for PI511384 responses to *Pst* under HT.

Response: Thank you for making these points.

1) In the main text, we now state "At the 2-leaf (juvenile) stage, **PI511383 and PI511384** were resistant with infection types (or IT scores) between 1 and 5 to all nine tested *Pst* races (**PSTv-3, PSTv-4, PSTv-11, PSTv-18, PSTv-37, PSTv-41, PSTv-51, PSTv-52 and PSTv-172**). These races are virulent on a wide range of wheat germplasm (Supplementary Data 1), under low temperature (LT) **and/or** high temperature (HT) regimes."
2) Based on the 90K SNP Chip, Clae9 and PI511383 are indistinguishable; consequently, we think that these two accessions are nearly identical in genotype. In the main text, we added a sentence: "CIae9 and PI511383 are genetically similar or identical (explained below)".
3) Due to sometimes unanticipated lack of availability of plant and/or fungal genotypes, we don't have data for PI511384 responses to *Pst* under HT (see "and/or" edit above).

In the Supplementary Data 1, we now specify the *Pst* responses of each resistant parent.

I-4. L91: No data is presented for PI560536 in Table1 or Suppl. data 1 file.

Response: PI560536 was tested under natural conditions in the field. PI560536 was a susceptible parent for popB. Fine mapping was done in popA and popC from different parents. Therefore, we did not test PI560536 under controlled conditions.

To clarify this. We revised as "In contrast, all the tested *Ae. tauschii* subsp. *tauschii* accessions (AS87, PI486274 and PI560536) do not have the *YrAS2388R* gene[26], and were always susceptible **under either natural and/or controlled conditions** (IT scores=7-9; Figure 1a; Table 1)."

I-5. L87-88: The sentence "The transgene YrAS2388 was later shown to be effective …" should probably remove, as this this is not an appropriate place (to early) to talk about this result.

Response: The sentence was deleted. The same information was provided later in the validation section. "… and tested the T_3 plants against **nine *Pst* races (PSTv-37, PSTv-39, PSTv-47, PSTv-143, PSTv-221, PSTv-239, PSTv-306, PSTv-352 and PSTv-353)** and *Pst* spores from the field, which represent a broad range of virulence (Supplementary Data 1)."

I-6. L93: "the same gene" or different allelic variants?

Response: We changed "the same **gene**" TO "the same **locus**."

I-7. L97: "'Langdon'-based hexaploid wheats" - what does this mean?

Response: "'Langdon'-based hexaploid wheats" means that they are synthetic wheats produced by 'Langdon' hybridized with different *Ae. tauschii* accessions.

To avoid confusion, we revised as "We additionally tested the **SHW accessions;** SW3 and SW58 are **both** derived from **the durum wheat 'Langdon' but have different D-genome donors:** the *Pst*-resistant CIae9 and the *Pst*-susceptible AL8/78, respectively."

I-8. L100-101: should probably read "when introgressed into certain hexaploid wheat genotypes".

Response: Incorporated

I-9. L102: replace "from" with "derived from".

Response: Considering the comment I-10, we deleted this paragraph.

I-10. L102-108, Figure S1: Not sure why this data is needed and what can be concluded from this. This data is not discussed or mentioned in the rest of the manuscript. Does this mean PI511384 contains an additional ASR gene or what?

Response: Thanks. We agree that these data do not add much to the manuscript. The paragraph and the associated figure were deleted.

I-11. L114-115: How is it possible to generate a linkage map with a cumulative map distance of 1,145 cM based on the genotyping of only 34 F2 plants?

Response: This is a valid point, and it concerned us. In the last version, we did not state that we also did bulk segregant analysis (BSA); both datasets gave us the same results for the *YrAS2388* locus. And, indeed the BSA are more robust. Thus, we deleted the original Figure S2, the original Supplementary Data 2, and methods, but **added the Supplementary Table S1**. We provide a major revision for the related sections.

In the result section, we revised as below.

"Using the 10k SNP assay, we genotyped 17 *Pst*-susceptible F_2 plants, ten from popA (PI511383/PI486274) and seven from popB (CIae9/PI560536), for bulked segregant analysis (BSA) of the *YrAS2388S* gene. The infection types (IT) of the selected F_2 plants were obtained in the field in 2011. Among 3,276 SNP loci inspected, the frequency of their association with a homozygous "B" (=*Pst*-susceptible phenotype) was sorted in descending order. Interestingly, eight of the top 20 SNPs, including AT4D3406, AT4D3410, AT4D3411, AT4D3412, AT4D3413, AT4D3417, AT4D3418 and AT4D3419 (Table S1), were mapped to the 4DS distal region[40]. In combination with specific genotypes of the linked markers, we mapped the *YrAS2388* locus distal to the AT4D3406 marker (Table S1)."

In the methods, we added the following information.

"**Bulked segregant analysis of the *YrAS2388* gene**

Genomic DNA was extracted using the Sarkosyl method[20], quantified on a ND-1000 spectrophotometer (Thermo Fisher Scientific, Wilmington, DE, USA), and diluted to 200 ng μl^{-1} for the Infinium iSelect genotyping. Single nucleotide polymorphisms (SNPs) were assayed at the Genome Center (University of California, Davis, CA, USA) according to the manufacturer's protocol (Illumina, Inc., San Diego, CA, USA). Normalized Cy3 and Cy5 fluorescence for each DNA sample was plotted with the GenomeStudio program (Illumina), resulting in genotype clustering for each SNP marker[24, 118].

We initially performed bulked segregant analysis (BSA) on 17 *Pst*-susceptible F_2 plants, ten from popA and seven from popB, using the 10K Infinium iSelect SNP oligonucleotide array. Starting with the 8,608 SNP loci, we sequentially eliminated: 1) 887 SNP loci that had missing data or were heterozygous on any parent line, 2) 308 SNP loci that were polymorphic between the two susceptible lines (PI560536 and PI486274), and 3) 2,777 SNP loci were identical in the four parent lines (CIae9, PI486274, PI511383 and PI560536), 4) 1,360 SNP loci that had four or more missing data points amongst 17 *Pst*-susceptible F_2 plants. The remaining 3,276 SNP loci were used for BSA analysis. Among *Pst*-susceptible F_2 plants with a clear genotype, the frequency of a homozygous "B" genotype (=susceptible phenotyp) was calculated and sorted in descending order for each SNP. The top twenty SNPs were prioritized for further analysis."

I-12. L123-124: "In nature, the large 4DS QTL is inherited as a Mendelian trait and masks other minor QTL". Not sure I understand the meaning of this sentence.

Response: Also, please see our response to I-11. We made a major revision of this section. It is not an issue anymore.

I-13. L127-128: "Thus we conclude that the 4DS QTL is the *YrAS2388* gene, and that the gene is common in the *Ae. tauschii* ssp. *strangulata*." This could only be hypothesized as no allelism test has been carried out to prove this.

Response: Please see our response to I-11. We made a major revision of this section, and it is not an issue anymore. Just for your information, we previously performed an allelism test for the *YrAS2388* gene between CIae9, PI511383, PI511384 and eleven other accessions. Based on the allelism test, the *YrAS2388* gene is common in *Ae. tauschii* subsp. *strangulata*[24].

I-14. L135: Please explain how the additional markers were generated.

Response: We incorporated the following information in the text.

"Additional markers were designed from the linkage map[40] and genome sequence[44] of *Ae. tauschii*. First, we retrieved the AT4D3403, AT4D3404 and AT4D3405 (Figure S1a) corresponding genomic sequence[40], prioritized the low-copy number regions for marker development, created ten PCR markers (*Xsdauw86* to *Xsdauw91*, *Xsdauw93*, *Xsdauw95* to *Xsdauw97*) among six parental lines, and located *YrAS2388* in the interval between *Xsdauw91* and *Xsdauw96* (Figure S1d). Second, we constructed a fosmid genomic library from the *Pst*-resistant genotype PI511383. Two additional markers (*Xsdauw92* and *Xsdauw94*) were developed using the fosmid clones of the *YrAS2388* region. After analyzing 4,205 popA $\mathbf{F_3}$ plants, that were from 11 F_2 plants heterozygous in the *Xsdauw2a-Xsdauw36a* region, we precisely mapped *YrAS2388* between *Xsdauw92* and *Xsdauw96*, about a 0.13-cM interval, and saturated this interval with three completely linked markers to *YrAS2388* (Figure 1b; Figure S1d; Supplementary Data 2). "

I-15. L136: "4,205 popA plants". What generation of plants of used, F2 or F3?

Response: They are F_3 plants. The information was added to the text (as in our response to I-14).

I-16. L140-141: "After assembling physical maps … we primarily analyzed the Xsdauw91-Xsdauw96 region". It is not clear why this region was selected. There are appears to be no recombinants identified between CNL-4DS1 and MLK-4DS-1. Therefore, the latter could also be considered a candidate R gene.

Response: We apologize for the imprecise language. **"we precisely mapped *YrAS2388* between *Xsdauw92* and *Xsdauw96*, about a 0.13-cM interval."** In this case, *Xsdauw96* is the proximal flanking marker. Therefore, any genes in the *Xsdauw92-Xsdauw96* interval, including $RLK_{4DS\text{-}2}$ and $NLR_{4DS\text{-}1}$ genes, are candidates of *YrAS2388* gene. $MLK_{4DS\text{-}1}$ is proximal to the *Xsdauw96* marker, and is not a candidate. To avoid confusion, we updated the ***Xsdauw96* marker with one between** $NLP_{4DS\text{-}1}$ and $MLK_{4DS\text{-}1}$. (updates are made in Figure 1b and Supplementary Data 2).

I-17. L163-165: where do these new markers (HTM) come from, and what is their physical location in the *YrAS2388 region*?

Response: We apologize. We added this information in Methods: **"Haplotype markers (HTM) were specifically designed for *RLK_{4DS-1}*, *RLK_{4DS-2}*, and *NLR_{4DS-1}***. Their physical locations (in Tables S2 and S4 and Supplementary Data 4) are counted from "A" in the start codon (ATG) in the genomic allele (GenBank accession number MK288012); for each marker, two periods (..) separate the starting and ending nucleotides, and a "-" indicates a backward count from "A" and a "+" indicates a forward count from "A"."

Their physical location in a specific gene is indicated in Tables S2 and S4, and in the Supplementary Data 4.

I-18. L178: "after mutagenesis". What type of mutagenesis was used here?

Response: Revised as "After **ethyl methanesulfonate (EMS)** mutagenesis."

I-19. L198-203: not sure the analyses carried out here are legitimate. Every wheat genotype has different sensitivity to ems, and at concentration of 0.8% ems may induce very different rates of mutation in different genotypes. So that 1 mutation per each 30kb may only be achieved in that specific genotyped analysed in ref. 38.

Response: Thank you for this comment. The average EMS mutation frequency apparently ranges from 1 in 17.6 kb to 1 in 37.5 kb[55, 56, 57, 58]. We recalculated the chi-square using the 1 in 17.6 kb rate, which is still significant at the alpha=0.05 level. To avoid further confusion and to condense the manuscript, we decided to delete this part of the manuscript.

I-20. L222-230: I do not understand these experiments at al. Restriction digestion should remove an entire wheat genomic DNA fragment rather than specific genes. This is assuming the diagram shown in Fig S7 is correct.

Response: We carefully selected combinations of restriction enzymes that would cleave at least one of the genes in the F2-1 plasmid. As background here, this experiment was designed to identify which of the three genes in the F2-1 plasmid confers *Pst* resistance. Fig. S6 shows the restriction sites on the F2-1 plasmid. When the plasmid is completely digested, the DNA will consist of a set of linear fragments that are used for genetic transformation. For example, with the *Bsr*GI digestion, both *RLK_{4DS-1}* and *NLR_{4DS-1}* genes are cleaved and thus non-functional but the *RLK_{4DS-2}* gene is intact and thus potentially functional. After transformation of wheat with the *Bsr*GI-digested F2-1, we expect that transgenic plants may contain a functional *RLK_{4DS-2}*, but should not contain either a functional *RLK_{4DS-1}* or a *NLR_{4DS-1}*. In this way, we generated a transgenic plant that had only one or two active genes amongst the three candidates in the plasmid F2-1. Thus, by comparing *Pst* phenotypes with wheat genotypes, we could deduce which candidate gene does or does not confers *Pst* resistance.

I-21. L230-231: what promoter was used to drive the expression of TV1 or TV2 in the transgenic plants? These experiments are is not detailed enough.

Response: The maize ubiquitin (*Ubi*) promoter was used. Information is added in text, method and tables (Tables 2 and S3).

I-22. L273: "TV1 was expressed more in the seedling stages and less in the older plants". I disagree with this statement. Only in L6 but not in L8 the TV1 expression seems to be low. Could it be an artefact of PCR? To firmly conclude on this, it is necessary to carry out a qRT-PCR analysis followed by assessing the data statistically to confirm/refute the above statement.

Response: Thank you for this comment. We have now performed independent trials with qRT-PCR and concur that the result with L6 was an error. We rewrote the text and figure legend. In the updated Figure S3, the c-e sections were replaced with the qRT-PCR data.

I-23. L275-276: Again, a potential difference in TV2 expression between the different samples/conditions needs to be verified by qRT-PCR.

Response: Thank you. This is addressed as in I-22.

I-24. L285-295: The yeast two-hybrid analysis data doesn't seem to provide any useful insight regarding the mechanisms underlying *CNL-4DS-1* mediated resistance.

Response: That's true. We removed all unnecessary panels from the figure to make it more assessable. Nonetheless, we would like to retain some of the Y2H because we believe that it provides some evidence that supports our mode of action.

I-25. 1) Figure 1: What isolate of *Pst* was used to generate Fig 1a? Were the plants naturally infected or in the lab? 2) Fig 1c need to show direction of each genes (as in Figure S4). 3) How was the sequence of MLK-4DS-1 gene determined as the two cosmids Fe-19 and Fa-13 do not overlap, and only one Fa-13 (according to the diagram) contains a partial sequence of this gene? 4) A term "paralogous 3'UTRs" is confusing. Suggest indicating in Fig 1e the location of primers P197 and P198 used for the analyses of 5 alternative transcripts.

Response to: 1) Information added: "to natural spores in the field." 2) Direction added. 3) The 3.9-kb gap was closed by PCR and sequencing. This information is added to the figure legend. The Fa-13 clone contains the 5' end (841 bp) of this gene. 4) We replaced "paralogous" with "duplicated". P197 and P198 were added to the figure.

I-26. Figure S2: What crosses were used for this QTL analysis, and how many F2 plants were used?

Response: We made a major revision of this section. The QTL analysis is replaced with BSA analysis. We added population and individuals to the text: "Using the 10k SNP assay, we genotyped 17 *Pst*-susceptible F_2 plants, ten from popA (PI511383/PI486274) and seven from popB (CIae9/PI560536) for bulked segregant analysis (BSA) of the *YrAS2388* gene."

I-27. Figure S3: what generation of plants (for each of the 3 crosses) were used?

Response: F_2 plants in popA-1 and popC-1; F_3 plants in popA-2. Information was added to the Figure S1 and the figure legend.

I-28. Figure S4 is very confusing. Where does the marker O13 come from? The multicolour scheme used needs to be explained in the legend.

Response: We apologize. We modified the figure, which is now Figure S2. Basically there are four physical maps and one composite diagram. *O13* was designed from the *NLR*$_{4DS-1}$ gene, which was identified during fine mapping. The following information was added to the legend: "***O13* was originally designed to amplify the *NLR*$_{4DS-1}$ gene with primers P160 and P161 (Supplementary Data 8); *O13* is present in both the *NLR*$_{4DS-1}$ and the *NLR*$_{4DS-2}$ genes.**"

The color scheme is explained in the legend: "**We illustrate the *YrAS2388* region in four selected genotypes: *Ae. tauschii* accessions AL8/78 (in purple) and PI511383 (in green); synthetic hexaploid wheat W7984 (in blue), and common wheat 'Chinese Spring' (in brown)** …. A composite map (**the top diagram in black**) was made using the sequence data from the synthetic hexaploid wheat W7984[59] (**four scaffolds in blue**), PI511383 (**three fosmids in green**), common wheat 'Chinese Spring' (**one contig in brown**; IWGSC RefSeq v1.0[116]) and the *Ae. tauschii* accession Al8/78[41] (**one contig in purple**)."

I-29. Figure S5: need to define the abbreviations used e.g. TV1, TV2, etc.

Response: Thanks. Information added to the figure legend.

I-30. Many supplementary data files are too large, and I am not sure whether all these are needed.

Response: Thanks. We agree. We removed supplementary data 2 and 6.

II. Reviewer #2 (Remarks to the Author):

Zhang et al., describe the mapping and cloning of the 4HB-NLR YrAS2388, which provides resistance to Puccinia when transformed into wheat and barley. The authors detail the characteristics of the gene and protein, with special interest given to the apparent splice variants, a cryptic intron and the four-helical bundle N-terminal domain.

Considering that the gene confers broad, durable resistance, it is very surprising that it is not in any hexaploid wheat lines. This could suggest that it is linked to very detrimental traits. Therefore, the GMO route may be the only way to go.

Though the work is good, the writing is very long winded, they focus on the wrong parts of the story and the whole thing could be condensed significantly.

Response: Thank you for your comments.

Regarding the absence of *YrAS2388R* in hexaploid wheat, we agree that the *YrAS2388R* gene may have linkage drag, particularly when it is in a hexaploid background. And we agree that GMO or genome editing may be an effective strategy to use this gene in hexaploid wheat.

We have condensed the narrative.

Notes to Authors:

II-1. 1) It is described that there are DNA polymorphisms between YrAS2388-containing lines and non-containing lines. What are these polymorphisms? What do they suggest about the function of YrAS2388? This is interesting information that is left out. 2) In addition to the GenBank accession for the functional *YrAS2388* allele, they should submit the versions that they sequenced that appear to be non-functional for reference and include a figure showing where the mutations are in the gene.

Response to: 1) Thank you. We prepared a haplotype map of the NLR_{4DS-1} gene in nine selected genotypes. Crucial polymorphisms are shown in the map (Supplementary Data 3). Yes, the haplotype map suggests that the expression of NLR_{4DS-1} is important for the *YrAS2388*-based *Pst* resistance. The revised narrative is: "However, there was substantial DNA and protein variation between the *Pst*-resistant (PI511383 and PI511384) and *Pst*-susceptible (AS87 and AL8/78) genotypes that express NLR_{4DS-1} (Supplementary Data 3); in the NLR_{4DS-1} gene, PI511383 and AL8/78 are only 94% and 91% identical in cDNA and protein, respectively." 2) As suggested, we submitted susceptible alleles of the NLR_{4DS-1} gene to GenBank and revised the text as: "GenBank accessions include MK288012 for the fosmid F2-1 (=PC1104), MK288013 for the *YrAS2388R* contig, **MK736661-MK736663 for the *YrAS2388R* gene in PI511383, CIae9 and AS2388, and MK736664-MK736666 for the *YrAS2388S* gene in PI560536, PI486274 and AS87, respectively**." Crucial variations between *Pst*-resistant and *Pst*-susceptible alleles are illustrated in the haplotype map (Supplementary Data 3).

II-2. Moreover, they frequently refer to organisms not having *YrAS2388/CNL4DS-1*, but this is not true, they just seem to have different versions that do not perform the resistance function you are assaying for. Therefore, it is improper to write this throughout the manuscript. They fix this in the paragraph starting at line 296 but should be fixed elsewhere.

Response: Thank you. We classified *YrAS2388* into the *YrAS2388*-resistant allele (*YrAS2388R*) and the *YrAS2388*-susceptible allele (*YrAS2388S*). We now state that the *YrAS2388R* allele is not present in certain germplasm. We fixed this problem throughout the manuscript.

II-3. Line 24-25: Describe what *YrAS2388* is. i.e., an NLR. This also makes it unnecessary to refer to the NB and LRR domain in the abstract, so you can rewrite to focus just on the four-helical bundle that makes it interesting.

Response: Suggestion adopted

"… we reveal that *YrAS2388R* encodes **a nucleotide oligomerization domain (NOD)-like receptor (NLR) with a N-terminal four-helical bundle** (4HB which was previously classified as a coiled-coil)"

II-4. Lines 37-39: Weirdly small first paragraph

Response: Paragraph modified

"**Wheat (*Triticum* spp.) is the largest acreage crop in the world, with an approximate 220 million hectares in 2018, with a global production of 760 million tons in 2018, which places it second after maize[1].** As a staple food crop, wheat provides about 20% of global calories for human consumption[2]. **Because the world population is projected to increase nearly two billion people within the next three decades[3],** the increasing human population worldwide will place an even greater demand for wheat production globally."

II-5. Line 46-48: Sentence is long and confusing, please rewrite.

Response: Suggestion adopted

"In wheat, ASR confers high levels of resistance to specific *Pst* races, **but the underlying genes, such as *Yr9*[9], *Yr17*[10], and *Yr26*[7], are often circumvented by the emergence of new virulent races**."

II-6. Line 73: Undesirable is misspelt

Response: Corrected

II-7. Line 75: Add "rust" after "stripe"

Response: Added

II-8. Line 77: add "disease," after devastating

Response: Added as "the devastating wheat stripe rust disease"

II-9. Line 92: Reword sentence

Response: Modified

"AvSYr28NIL and AvS are near-isogenic lines (NILs) for the *Yr28* locus (As we explain below, *YrAS2388* and *Yr28* are proposed to be the same locus)."

II-10. Mentions that the SW3 line, derived from the resistant A. tauschii line AvSYr28NIL, is not resistant, therefore YrAS2388 is suppressed in hexaploid genotypes. However, it is not described how SW3 was derived from AvSYr28NIL. Is it possible that there were any crosses or genetic events that could have removed YrAS2388 from the AvSYR28NIL D genome? To say that it is being suppressed, they would need to sequence it to confirm it is present.

Response: Thank you. SW3 was derived from the durum wheat 'Langdon' and the *Pst*-resistant CIae9. This is a good point. We checked the *NLR4DS-1* gene in SW3. It is present and transcribed. We added the following information in the text.

"For example, the SHW line 'SW3' is derived from *Pst*-resistant *Ae. tauschii* CIae9[51], **harbors the *Pst*-resistant NLR_{4DS-1} allele, and shows alternative splicing of this gene**; however, SW3 was susceptible (IT scores=7-9) to *Pst* in Moscow, Idaho, USA (Table 1), presumably because of a suppressor in its genetic background."

II-11. Line 104: "Syn-SAU-93 only has adult plant resistance" and "Syn-SAU-93 is susceptible in the first leaf juvenile stage" are pretty much the same thing. Reword or remove one of these.

Response: In order to condense the manuscript, the paragraph has been removed.

II-12. Is the revelation of CIae9 being PI511383 necessary for this story? Removing it would make these sections streamlined. If kept, this should be mentioned at the beginning of the results and then only one of them should be mentioned for the rest of the paper unless there are any other differences found.

Response: Thanks for your suggestion. Mostly, this manuscript was based PI511383. However, there were two places where we used CIae9. In the BSA analysis, we included F_2 plants from popB (CIae9/PI560536). In addition, mutagenesis was partially performed in the synthetic hexaploid wheat SW3, which was derived from the durum wheat Langdon and the *Aegilops tauschii* accession CIae9. For these reasons, we prefer to keep it in the story.

In accordance with this comment, we now state that "**CIae9 and PI511383 are genetically similar or identical (explained below)**" in the first part of the results, and tried to only use PI511383 in the rest of the manuscript.

II-13. Furthermore, if CIae9 = PI511383, and PI486274 ~= PI560536, then the two populations, popA and popB are almost exactly the same. They even removed the SNPs that were polymorphic

between the two susceptible lines from the analysis. This makes the story a lot more confusing and could be condensed significantly!

Response: Thank you. We understand your point. To avoid confusion, we rewrote this part. For the BSA analysis, we used 3,276 SNPs that consistently separated *Pst*-resistant and *Pst*-susceptible genotypes. If we included the 308 SNPs that separate the *Pst*-susceptible genotypes, the analysis would be more confusing.

II-14. Line 114: Should clarify that it was 34 F_2 in total.

Response: After major revision, this is not an issue anymore.

II-15. Lines 129-137: Explanation of mapping is too brief, saying you used the data to map does not explain what you did.

Response: Thank you. We incorporated the following information in the text.

"Additional markers were designed from the linkage map[40] and genome sequence[44] of *Ae. tauschii*. First, we retrieved the AT4D3403, AT4D3404 and AT4D3405 (Figure S1a) corresponding genomic sequence[40], prioritized the low-copy number regions for marker development, created ten PCR markers (*Xsdauw86* to *Xsdauw91*, *Xsdauw93*, *Xsdauw95* to *Xsdauw97*) among six parental lines, and located *YrAS2388* in the interval between *Xsdauw91* and *Xsdauw96* (Figure S1d). Second, we constructed a fosmid genomic library from the *Pst*-resistant genotype PI511383. Two additional markers (*Xsdauw92* and *Xsdauw94*) were developed using the fosmid clones of the *YrAS2388* region. After analyzing 4,205 popA $\mathbf{F_3}$ plants, that were from 11 F_2 plants heterozygous in the *Xsdauw2a-Xsdauw36a* region, we precisely mapped *YrAS2388* between *Xsdauw92* and *Xsdauw96*, about a 0.13-cM interval, and saturated this interval with three completely linked markers to *YrAS2388* (Figure 1b; Figure S1d; Supplementary Data 2). "

II-16. Line 230: Couldn't this suggest that neither TV1 or TV2 could be the important isoforms?

Response: No, we don't think so. Indeed, the mutagenesis data indicate that TV1 is critical, and TV1 is the most highly expressed of the transcripts.

In the text, "We hypothesize that TV1 plays a major role in the induction of stripe rust resistance, TV2 acts as a positive co-factor, and TV4 (or possibly TV3) act either as negative regulators when its expression is high or as positive regulators when its expression is low (Figure S8)."

II-17. It is mentioned that CNL4DS-1 function is independent of RAR1, SGT1 and HSP90 due to a lack of interaction. However, this conclusion is flawed because evidence in R genes that do require these co-chaperones, e.g., the Mla locus, do not appear to interact when tested in their full-length forms but do so when only their LRR domains are expressed. If the authors wish to test this, they should repeat the interactions with a deletion series of TV1, especially sections of the LRR domain.

Response: Thank you. We agree. In order to condense the manuscript, we removed this part.

II-18. It would also make the figure much clearer if they showed a diagram of the gene regions used for Y2H, rather than just abbreviations (i.e., TV1, TV2)

Response: The current Figure S7 was modified as suggested.

II-19. Additionally, could it be possible that the mutant versions of TV2a actually sequester the non-mutant versions of TV1, and thus lower their activity? Therefore, acting as the suppressor you have implicated?

Response: Thank you for this idea; we have now presented a plausible model (Figure S8), which is somewhat similar. Currently, our evidence indicates that multiple isoforms are essential for resistance, but how they interact, by competition and/or sequestration, is unknown. We believe that it will take many years to fully understand the actual mechanism of action.

II-20. Line 386-389: Long convoluted sentence, please rewrite.

Response: Thank you. Revised. "The "coiled coil" domain of the potato virus X resistance protein (Rx) actually forms a four-helix bundle (4HB)[97]. We note that the N-terminal domain of NLR_{4DS-1} is now classified as a Rx-CC-like in the NCBI CDD ($E=9\times10^{-9}$) and Rx_N in the Pfam database ($E=6\times10^{-16}$). Furthermore, the N-terminal domain of NLR_{4DS-1} is predicted to fold into four helixes."

II-21. They should mention how their observations of *YrAS2388* acting after 5dpi (line 105-108) suggest what the mechanism of action of the protein is.

Response: Thank you for your suggestion. We now included 5dpi and more time points in qRT-PCR (Figure S3). At 5 dpi, TV2 transcript increased, which agrees with the up-regulation of TV2 under higher temperature. From these result, we believe that TV2 acts as a positive co-factor for stripe rust resistance. We have revised our text.

III. Reviewer #3 (Remarks to the Author):

This work describes the isolation by positional cloning of a novel gene conferring stripe (yellow) rust resistance in wheat. Mutational analysis and transformation experiments confirmed the identity of the cloned gene. The gene YrAS2833 is derived from the wheat D genome ancestor species Ae. tauschii and encodes a canonical NLR immune receptor. Several splicing variants were identified and characterized for function and expression. Furthermore, the gene contains a duplicated 3' region. The gene is common in Ae. tauschii and synthetic wheat, but absent in all other bread wheat genotypes tested. This suggests that it was never incorporated (or was lost) in the hexaploid gene pool. No negative effect on yield and other studied agronomic traits was found.

The work is novel and of interest to the community. There are very few Yr resistance genes cloned and there is little information on their diversity and usefulness for breeding. The work presented in this manuscript adds highly valuable information and is of relevance for the characterization and practical use of genes in resistance breeding. However, the current manuscript is in many aspects unclear and lacks a detailed description of the work and the performed analysis.

Response: Thank you. We added details in the revised version, as indicated in the response to more specific comments.

Essential revisions

Alternative splicing:

III-1. This is an important part of the data but it is presented and described in insufficient detail. Much of lower part in Figure 1 needs clarification and expansion.

Response: Thank you. We revised the figure legend and Figure 1d-e.

"(**d**) Genomic structure of *NLR_{4DS-1}* in PI511383. The conserved domains and the duplicated 3'UTRs are labelled; **their approximate genomic locations are highlighted with dotted lines.** The 3'UTR duplication was caused by a 2,668-bp insertion (red region), which has three regions (with a prime symbol) similar to exons 5, 6 and 7. Intron 7a and 7b, and exon 8 are **the original components of the ancestral 3'UTR, but the 2,668-bp insertion disrupted the ancestral 3'UTR and then formed two 3'UTRs, each containing both ancestral (black dots) and inserted (red dots) segments. The cryptic intron in exon 5 is highlighted by a gray box. Intron 7a, 7a' and 7b are highlighted by size bars below their name.** (**e**) Transcript variants of *NLR_{4DS-1}* in accession PI511383. Cloning and sequencing of the *NLR_{4DS-1}* cDNA clones identified five transcript variants, designated TV1 to TV4b, of *NLR_{4DS-1}* in accession PI511383. Grey boxes indicate portions of the retained intron in mature messenger RNA. **Rectangles and straight lines indicate regions present in mRNA; the caret-shaped lines represent regions that are absent in mRNA. Part of the cryptic intron in exon 5 is retained in TV3. The TV4a and TV4b encode an identical protein, called TV4. P197 and P198 are primers for detecting all five splicing variants in one PCR**. Abbreviations include exon (E), four-helical bundle (4HB), intron (In), leucine-rich repeat (LRR), two miniature inverted-repeat transposable elements (**M1 and M2**), nucleotide-binding (NB), and start (↓ in blue) and stop (↓ in red) codons."

III-2. In Fig1d, the dotted description of the protein structure is unclear. Is the length of the LRR domain really very similar to the 4HB domain? This is hardly possible.

Response: Thank you. The original diagram was based on the NCBI blast result (pasted below). In response to this comment, we analyzed the protein sequence manually. Whether or not the LRR region should extend further to the 3' is unclear; there are some additional repeat modules toward the C-terminus but they don't have the exact LxxLxLxxN signature {Padmanabhan, 2009 #6168}.

We revised the figure, and we also indicate in the revised legend: "their **approximate** genomic locations are highlighted by dotted lines.

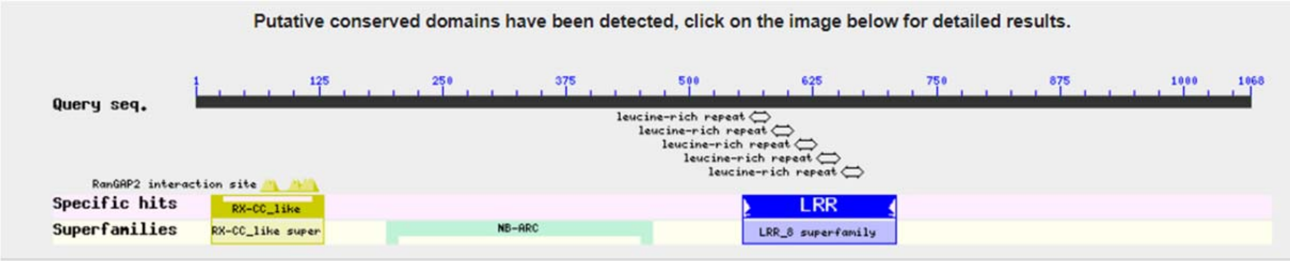

III-3. From where does the large red sequence in TV1 and TV2 derive from? It is impossible to correlate the information with the intro exon structure in Fig1d.

Response: We apologize. We changed the red boxes to lines. We also added "Rectangles and **straight lines** indicate regions in the mRNA; the caret-shaped lines represent regions that are absent in mRNA."

III-4. What does the labeling TV2a and TV2b mean in the figure. What is the difference between the two forms?

Response: When the cryptic intron in TV2 is skipped, there are two potential ORFs. We originally termed the first ORF TV2a and the second ORF TV2b. As in our comment regarding III-6, we treated the TV2b ORF as a pseudo product. However, there is only a single TV2 transcript. To avoid confusion, we have removed TV2b, the pseudo product.

III-5. TV3: according to Fig1d there is a cryptic intro in E5. What is then the cryptic intron in TV3? Is it only partial, merged with which red exon? This is completely unclear.

Response: TV3 contains only part of the cryptic intron. Information was added to the legend: "Part of the cryptic intron in exon 5 is retained in TV3."

III-6. In the figure legend of Figure 1 pseudogenes are mentioned. This is not described in the text and the reader wonders what else is lacking in the main text. Why are these genes pseudogenes? The text should allow the reader to follow the arguments of the authors.

Response: We apologize. We originally treated TV2b as a pseudo product. We have now removed TV2b from the entire manuscript because it is irrelevant

III-7. Figure legend 1e lacks details (explanation of red segments, start and stop codons both with down arrows? There are also up arrows, what do they mean? What does M1 and M2 mean?

Response: We revised the figure: red segments were replaced with red lines that match 1d, and up arrows were removed. We also added more information in the legend: "two miniature inverted-repeat transposable elements (M1 and M2)."

III-8. The whole Figure 1d and e needs to be redrawn. Possibly, and for the sake of clarity, this should be an own figure, providing much more detail and information.

Response: Thanks. We modified Figure 1d and the legend. For clarity, we prepared a supplementary Figure 9a that resembles 1d but has many more details.

III-9. Most importantly, the expression levels of the five different splice variants must be quantified by qRT-PCR. For none of the experiments and information in supplementary figures quantitative data are shown. Rather, unprecise description is provided, e.g. L273. It is possible that some variants are only present in very minor amounts and are simply due to inefficient splicing. This must be clarified and the data must be added.

Response: Thank you. We performed qRT-PCR, rewrote the results, revised the Figure S3c-e, and rewrote the figure legend. From the current data, it appears that the two variants, TV2 and TV3 with low levels of expression, are not due to inefficient splicing.

In results, we revised as below.

"In *Pst*-resistant PI511383, expression of the NLR_{4DS-1} variants is regulated by both endogenous and external factors. All four major transcripts (TV1 to TV4) were expressed in both the seedling and in the adult leaves (Figure S3c). When exposed to alternating low (10 ^{o}C) and high (25 ^{o}C) temperature, the high temperature caused an increase in the expression of TV2 and a decrease in the expression of TV4 (Figure S3d), which is correlated with increased *Pst*-resistance at elevated temperatures. We then studied how *Pst* strain PSTv-306 affects NLR_{4DS-1} expression. TV1 appears to be constitutively expressed; their levels in the *Pst*-infected plants were comparable to those in the mock-inoculated control plants (Figure S3e). In contrast, in *Pst* infections TV2 increased at 2, 5 and 10 days post inoculation (2, 5, 10 dpi) but not at 3 dpi, TV3 decreased at 3, 7, and 14 dpi, and TV4 decreased at 3 dpi (Figure S3e). Thus, both temperature and *Pst* infection regulate the transcription of NLR_{4DS-1}. However, a change in the relative levels of either the four transcripts and/or the proteins or protein complexes may affect the induction of stripe rust resistance."

The Figure S3c-e legend is added as below.

“ (**c-e**) cDNA expression of four *NLR*$_{4DS-1}$ isoforms in PI511383. Primers used were P215 and P216 for *NLR*$_{4DS-1\ TV1}$, P217 and P218 for *NLR*$_{4DS-1\ TV2}$, P219 and P220 for *NLR*$_{4DS-1\ TV3}$, P221 and P222 for *NLR*$_{4DS-1\ TV4+}$, and P223 and P224 for *ACTIN*. TV4^{+} also contains TV3, but TV3 only accounts for 2-5% of the total transcripts. Pairs of treatments (juvenile- vs. adult- stages; 10 ^{o}C vs. 25 ^{o}C; *Pst*- vs. mock-inoculation) that are marked with an asterisk are significantly different (α=0.05) by Student's t-test. (**c**) Developmental responses were conducted on newly expanded leaves at the juvenile (2 and 4-leaf) and adult (6 and 8-leaf) plants which were maintained at 25 ^{o}C during the day and 15 ^{o}C at night with a 16 h photoperiod. (**d**) Temporal responses were conducted on the 4th-leaf at the 5-leaf stage. Plants were alternated every 24h between 10 ^{o}C (day 1 and day 3) and 25 ^{o}C (day 2 and day 4), each with an 16-h photoperiod. (**e**) Responses to *Pst* were determined at the 2-leaf stage. After plants were mock- or inoculated with PSTv-306, they were maintained at 10 ^{o}C in darkness for 48 h, and were then maintained under a low temperature cycle (4 ^{o}C / 20 ^{o}C) with a 16-h photoperiod. A mock- or inoculated leaf was sampled after 1, 2, 3, 5, 7, 10 and 14 days post inoculation (dpi). Six biological replicates were used for each data point. (**a-e**) All samples except those at 1 and 2 dpi were collected in the middle of the indicated photoperiod. Samples at 1 and 2 dpi were collected in the 10 ^{o}C dark cycle. Abbreviations include base pairs (bp), resistance (R) or susceptible (S) to *Pst*, transcript variants (TV1 to TV4 of *NLR*$_{4DS-1}$), days post inoculation (dpi) and molecular standard (M=2-Log DNA Ladder, New England Biolabs, Beverly, MA, USA).”

III-10. The model presented in L283 should then be based on better data.

Response: Thank you. We agree. A new model is proposed. It is based on qRT-PCR and Y2H results.

In this new model, “We hypothesize that TV1 plays a major role in the induction of stripe rust resistance, TV2 acts as a positive co-factor, and TV4 (or possibly TV3) act either as negative regulators when its expression is high or as positive regulators when its expression is low (Figure S8).”

III-11. L109 ff: the development of populations is not clear and the reader has to go to Materials and Methods to find out what was done. The basic information on population development must be given here.

Response: Thank you. Information was added. “To map the resistance gene, **three F$_2$ populations** were constructed; in **popA (PI511383/PI486274)**, **popB(CIae9/PI560536)**, and **popC(PI511384/AS87)**, the *Pst*-resistance trait is inherited as a Mendelian trait[26]. Here, among 1,910 F$_2$ plants of **popC-2 (PI511384/AS87)**, 1,432 were resistant and 478 were susceptible in Wenjiang, Sichuan, China, which is consistent with the expected 3:1 ratio for single gene inheritance ($\chi^2_{3:1}$=0.001, *P*=0.98).”

III-12. 1) L. 140: Give explanation why “we primarily analyzed the … region”. According to the physical map in Fig1c Xsdauw96 is a sequence located in the candidate gene (is this an intragenic recombinant, similar to Huang et al. Genetics, 2003, http://www.genetics.org/content/164/2/655.short; cloning of Lr21 from Ae. tauschii?). The description and logic of the presentation needs more detail and clarify. 2) Is the gap in the physical map of the fosmids a problem, as the first flanking marker not located in the CNL candidate gene is on the right of the gap?

Response to: 1) Yes, the closest crossover must be an intragenic recombination within the *NLR$_{4DS-1}$* gene. During our cloning of the male-sterility gene *MS2*, we also identified an intragenic recombination in the *MS2* gene (Nature Communications 8: 15121). This occurs when a large mapping population is used, for example 4,205 F_3 plants in this study.

This already confused the first reviewer (see comment I-16) and possibly future readers. To avoid confusion, we updated the ***Xsdauw96* marker with one between** *NLR$_{4DS-1}$* and *MLK$_{4DS-1}$*. Updates are made in Figure 1b and Supplementary Data 2.

In accordance with this comment, we added the following information: "After assembling physical maps (Figure 1c; Figure S2), we noted that the two overlapping fosmids, F2-1 and Fe-19, contained both *Xsdauw91* and *Xsdauw96* markers, which also contained the minimal interval of *YrAS2388*. After sequencing F2-1 and Fe-19, we primarily analyzed the *Xsdauw91-Xsdauw96* region (ca. 50kb)."

2) Thanks. The gap was filled by PCR clones and sequencing. We modified the figure and legend.

III-13. The description of the mutants is poor. Where does the reader find the information on the mutations (affected amino acids)? The main text refers to Supp data 5, but there the text refers to Supp table 1, which does not contain the information. The whole part has to be rewritten and clarified.

Response: All mutation data is in the Supplementary data 5. We revised the manuscript and provided more details in the Supplementary data 5.

Protein variations are added as below.

"Eighteen *Pst*-susceptible mutants had at least one base change in the *NLR$_{4DS-1}$* gene, and 16 of those mutations (89%) either caused an amino acid change or formed a premature stop codon (Supplementary Data 5). **Seven types of amino acid variations were identified: Gly117Asp, Val267Ile, Leu421Phe, Val482Ile, Ser394Asn, Thr456Ile and Gln557Stop(*) (Supplementary Data 5**)."

"**In *Pst*-susceptible mutants, of the seven types of amino acid variations, Ser394Asn and Gln557Stop(*) only affect TV1 and Thr456Ile only affects TV4, which indicates that both TV1 and TV4 are essential for stripe rust resistance (Supplementary Data 5).** Collectively, we hypothesize that TV1 plays a major role in the induction of stripe rust resistance, TV2 acts as a positive co-factor, and TV4 (or possibly TV3) act either as negative regulators when its expression is high or as positive regulators when its expression is low (Figure S8)."

III-14. L211: Transgenic families 5 and 10 are mentioned. But they are not introduced and not described. The description of the transgenic validation experiment is basically missing.

Response: We apologize. We revised the results, figure legends, and supplementary data notes.

In results, we revised as below.

"After bombarding 1,590 wheat immature embryos, we obtained 24 putative transgenic plants. All three target genes, *NLR$_{4DS-1}$*, *RLK$_{4DS-1}$*, and *RLK$_{4DS-2}$*, were detected in four T_0 plants (No. 4, 5, 10 and 22), but only two plants (No. 5 and 10) were positive for the *NLR$_{4DS-1}$* cDNA (Table 2; Figure S4). For No. 5 and 10 transgenic plants, we selected 13 subfamilies that were homozygous resistant to PSTv-40 in the T_2 generation, and tested the T_3 plants against nine *Pst* races (PSTv-37, PSTv-39, PSTv-47, PSTv-143,

PSTv-221, PSTv-239, PSTv-306, PSTv-352 and PSTv-353) and *Pst* spores from the field, which represent a broad range of virulence (Supplementary Data 1)."

We also added "**all from the No. 5 and 10 T_2 subfamilies**" to the Figure 2b and S5 legends and the Supplementary Data 1 notes.

II-15. As already stated above for Fig1e, the description of alternative splicing lacks detail and clarity.

Response: We revised the abstract and the text as below.

In abstract: "Unlike the *YrAS2388*-susceptible allele (*YrAS2388S*), the resistant allele *YrAS2388R* evolved with duplicated 3' untranslated regions (3'UTR1 and 3'UTR1); each is associated with alternative splicing in the nucleotide-binding domain: two with 3'UTR1 and three with 3'UTR2."

In results: "Based on RNA-seq and RT-PCR, the *Pst*-resistant NLR_{4DS-1} (i.e., in PI511383 and PI511384) has duplicated 3'UTRs (Figure 1d-e), while the *Pst*-susceptible genotype that expresses NLR_{4DS-1} (e.g. AL8/78) has a single 3'UTR. In the *Pst*-resistant NLR_{4DS-1}, the duplicated 3'UTRs each are associated with alternative splicing in the NB domain, resulting in five transcript variants (TV1, TV2, TV3, TV4a and TV4b) (Figure 1e)."

III-16. L381: these data mentioned in the discussion should be presented in detail in the results section.

Response: Thank you. We reorganized these data in the result section. We also prepared a phylogenetic tree (Figure S10) for the *Pst*-resistant NLR_{4DS-1} and their close homologues that are transcriptionally active genes in 'Chinese Spring'.

In results, we revised as: "In common wheat 'Chinese Spring' (CS), there are at least five transcriptionally active homologues of the NLR_{4DS-1} gene (Figure S10). None of the NLR_{4DS-1}-like homologues in CS have duplicated 3'UTRs. **T**he *Pst*-susceptible NLR_{4DS-1} homologues in CS shares 86%-94% identity with the *Pst*-resistant NLR_{4DS-1} in PI511383 at the cDNA level."

III-17. 1) The methods section (starting at L585) needs expansion and clarification. What sequences were used for which genes, transformations? 2) Bilaphos should be bialaphos? 3) The selection plasmids are not described at all, there are no references, etc.

Response: Thank you. 1) Text was revised as: "**The fosmid F2-1 (=PC1104; Table S3) from PI511383 is about 47.7 kb (GenBank MK288012). The F2-1 clone** contains genomic copies of ***RLK_{4DS-1}*, *RLK_{4DS-2}* and *NLR_{4DS-1}***. Both intact and/or the restriction enzyme-cleaved F2-1 were used for wheat and barley transformation." 2) Corrected. 3) We revised as "The fosmid F2-1 has no plant selection marker, and thus required co-transformation with PC174, which has the bialaphos (*BAR*)[122] and hygromycin (*HYG*)[123] selection markers **both under the CaMV *35S* promoter** (Table S3)." The vector backbone is pCAMBia1300 (Table S3).

Additional points / at least partially essential:

III-18. The gene YrAS2833 was suppressed in many hexaploid wheat genotypes. This suppression effect is reminiscent of the Pm8 suppression by Pm3 homoeologs (Hurni et al. 2014). As the authors have identified homoeologs/homologs of YrAS2833 in wheat (see L381, L695) mechanistic studies using the transient N. benthamiana could clarify the molecular basis of suppression. The identification of the suppressor gene would be of great value for breeders so they could avoid introgression of the gene into suppressive germplasm. As the manuscript puts a lot of emphasis on breeding applications, a diagnostic marker for an active suppressor gene would be highly desirable.

Response: Thank you for your comments. We understand that this is a very important issue. However, we really have no idea how suppression occurs. It could be from either linkage drag or from either the A or B genomes. As suggested, we are interested in studying the currently know homologs, and we could address this with wheat mutagenesis. However, we believe it will take some years to get a solid answer.

In discussion, we add sentences about this idea: "**To answer this question, in the future, one could mutagenize a SW3 line, screen for truncation mutations in the *NLR*$_{4DS-1}$ homologues, and test whether the homologues' mutations have any effect on stripe rust resistance**. Regardless, because the transgene *NLR*$_{4DS-1}$ induces effective *Pst* resistance in hexaploid wheat, we predict that sustainable *Pst* resistance can be achieved with either a cisgenic strategy with *NLR*$_{4DS-1}$ and/or a conventional strategy that combine both the incorporation of a resistant *NLR*$_{4DS-1}$ and the avoidance of the apparently linked latent suppressor(s) from *Ae. tauschii*."

III-19. L. 100: The homoeologs should be introduced here and presented as candidate suppressor genes. Furthermore, the origin of the suppressing hexaploid lines should be presented and possible progenitors of the suppressor should be identified. This part of the manuscript is too short and lacks details.

Response: We have tried to deal with this in a more straightforward manner. However, in the interests of streamlining the manuscript, we address this issue in the later sections in results. As we stated above, we believe that it will take quite a few years to get a solid answer about the suppressor and its mechanism.

The revised text is: "In Triticeae, there are multiple *NLR*$_{4DS-1}$-like genes; three copies were identified in the *YrAS2388* region (Figure S2). In common wheat 'Chinese Spring' (CS), there are at least five transcriptionally active homologues of the *NLR*$_{4DS-1}$ gene (Figure S10). None of the *NLR*$_{4DS-1}$-like homologues in CS have duplicated 3'UTRs. **T**he *Pst*-susceptible *NLR*$_{4DS-1}$ homologues in CS shares 86%-94% identity with the *Pst*-resistant *NLR*$_{4DS-1}$ in PI511383 at the cDNA level."

III-20. Supp Fig2: Please indicate the position of YrAS2833.

Response: In order to streamline the narrative, this figure was deleted from the revised manuscript.

III-21. Figure Legend Supp Figure 4: What do the colors mean? The whole figure needs more clarity (what is the correlation with Figure 1?) and description.

Response: We apologize. We modified the figure. Basically there are four physical maps and one composite diagram. The color scheme is explained in the legend: "**We illustrate the *YrAS2388* region in four selected genotypes: *Ae. tauschii* accessions AL8/78 (in purple) and PI511383 (in green); synthetic hexaploid wheat W7984 (in blue), and common wheat 'Chinese Spring' (in brown)** …. A composite map (**the top diagram in black**) was made using the sequence data from the synthetic hexaploid wheat W7984[59] (**four scaffolds in blue**), PI511383 (**three fosmids in green**), common wheat 'Chinese Spring' (**one contig in brown**; IWGSC RefSeq v1.0[116]) and the *Ae. tauschii* accession Al8/78[41] (**one contig in purple**)."

For Figure S2, we added the following description.

"All three genes are highly conserved among *Ae. tauschii* accessions and the co-localization of various genes in the *YrAS2388* region is highly conserved between *Ae. tauschii* and common wheat (Figure S2)."

"In Triticeae, there are multiple NLR_{4DS-1}-like genes; three copies were identified in the *YrAS2388* region (Figure S2)."

III-22. L 198: Does not fit in main text. It is sufficient to mention in the main text that EMS mutagenesis was used.

Response: Thanks. We have deleted this paragraph.

III-23. L264: Of, the… : needs rephrasing

Response: Thanks. Fixed. Rephrased to "In addition, the duplicated 3'UTR1 and 3'UTR2 DNA of NLR_{4DS-1} in PI511383 (Figure S9b) are only 87% identical in the conserved 373 bp."

III-24. L265: have … identity in the conserved region: the text needs clarification. Clearly, Fig1e does not give sufficient information to be able to follow the text.

Response: Thank you. We specified in this way: "In addition, the duplicated 3'UTR1 and 3'UTR2 DNA of NLR_{4DS-1} in PI511383 (Figure S9b) are only 87% identical in the conserved 373 bp (GenBank MK736661: 3735..4107 versus 6409..6781, counted forward from the start codon ATG)."

III-25. The final paragraph of the results starting L309 needs language editing.

Response: Some edits were made to this paragraph.

"We compared the stripe rust resistance in 81 SHW lines[52] and their original parents, including 30 SHW lines with the *YrAS2388R* gene (Supplementary Data 7). *YrAS2388R* confers a strong *Pst* resistance (IT scores=1-3) in *Ae. tauschii*[26]. However, 27% of SHW wheat had significantly less resistance than the parental lines (*T. turgidum* and/or *Ae. tauschii*). **For example, the SHW line 'SW3,' which is derived from *Pst*-resistant *Ae. tauschii* CIae9[51], has the *Pst*-resistant NLR_{4DS-1} allele, with the characteristic expression of alternatively spliced transcripts; however, SW3 was susceptible (IT scores=7-9) to *Pst***

in Moscow, Idaho, USA (Table 1), presumably because of a suppressor in its genetic background. Nonetheless, strong resistance to *Pst* in *Ae. tauschii* was frequently conferred as moderate to high *Pst* resistance in other SHW wheat (Supplementary Data 7), indicating that *Ae. tauschii* is valuable for breeding resistant NLR_{4DS-1}. For example, the SHW wheat 'Syn-SAU-S9' is based on LDN/AS313//AS2399, in which the *Ae. tauschii* AS2399 is positive for the *YrAS388R* gene[26]. Although Syn-SAU-S9 displayed only moderate resistance to *Pst* (IT scores=4-5), we used Syn-SAU-S9 to transfer the *YrAS388R* gene into common wheat. **Three co-segregating markers were developed for *YrAS2388R* (Figure S11; Supplementary Data 2); marker-assisted selection expedited the breeding process.** In 2015, we developed an elite line 'Shumai 1675', which is a F_6 line of 'Syn-SAU-S9'/'Chuan 07001'//'Shumai 969'. In 2017, 'Shumai 1675' outcompeted the check variety 'Mianmai 367' with an 11% increase in yield in the regional variety trials of the Sichuan province, China (Table S5)."

III-26. L366: Needs clarification.

Response: More specific information is provided as below.

"However, in 2018, the SHW 'SW3' adult plants were fully susceptible to natural *Pst* races in field (Moscow, ID, USA), probably because the suppressor responds more to the cooler night temperature in the Moscow area."

Reviewers' comments:

Reviewer #1 (Remarks to the Author):

The manuscript has been substantially revised, and somewhat simplified making the story easier to follow for the readers. It also appears that the authors have answered and/or reasonably well rebutted most of my comments. Nevertheless, some parts of the manuscript are still quite difficult to follow, and in my opinion the Abstract requires further major rewrite/improvement, spelling out novelty and importance of this study. From the Abstract alone it is not clear at all why this work should be published in such a high-ranking journal as Nature Communications? What makes this R gene so special? What key discoveries were made in this study making it stand out from other map-based gene isolation studies? Is this the fact that the cloned gene is a typical NLR, but instead of controlling gene-for-gene resistance shown for many other cloned R genes, YrAS2388 controls broad spectrum resistance? Or is it all about alternative splicing?

The resistance gene cloned should be referred to as a typical NLR (or NB-LRR with an N-terminal CC-domain). Referring to the N -terminal feature of the encoded R protein as a "four helix bundle" doesn't make it any more special. In fact, the N-terminal feature I believe should still be referred to as a coiled-coil (or CC) domain. This is true that CC domains of some NLRs display a four-helical fold in a solution. This is yet to be formally shown for YrAS2388.

When referring to various alleles of YrAS2388 please use "resistance gene/allele" rather than "resistant gene/allele" throughout the manuscript.

It would help the reader tremendously to more clearly indicate that alleles of YrAS2388 are present in Ae. tauschii as well as in T. aestivum, and in both resistant and susceptible genotypes. This is important. I'd suggest preparing a new figure showing a comparison of susceptibility and resistance versions of this gene, and the corresponding transcripts. Another useful figure I would like to see is the one showing an alignment of protein sequences encoded by susceptibility and resistance versions of YrAS2388 (this would include all "truncated" NLR versions potentially expressed from TV1 - TV4) and indicating the recognised protein domains and key motifs. How many leucine-rich repeats are present in the "full-length" YrAS2388?

I can't fully agree with defining the 833-bp intron as "cryptic". This intron is flanked by canonical GT-AG splice sites in YrAS2388, and it is correctly spliced in TV2. This intron is simply "retained" in TV1. This intron also must have an alternative 5' splice site (what it is? – please specify), which allows alternative splicing as in TV3. In addition, I guess that TV3 and TV4 resulted from exon skipping events. I feel this needs to be clearly explained in the manuscript text.

Now, the authors state (see L227-236) that Pst susceptible wheats AS87 and AL87/8 express only TV1 (an isoform that retains a 833-bp intron) and no fully spliced isoforms such as TV2. I therefore am puzzled as to why the complementation study was done using TV1? Wouldn't it be more logical to expect TV2 and/or TV3 and/or TV4 transcripts, that are not expressed in susceptible wheat genotypes, to play important role in Pst resistance?

Reviewer #2 (Remarks to the Author):

Though they fixed most of our issues, it appears our concern that the manuscript was long-winded and in need of being cut down was ignored. Not only did the authors add 70 lines, but they reduced the line spacing to make it seem like they had removed seven pages instead.

Notes to Authors:

It is described that there are DNA polymorphisms between YrAS2388-containing lines and non-containing lines. What are these polymorphisms? What do they suggest about the function of YrAS2388? This is interesting information that is left out. In addition to the GenBank accession for the functional YrAS2388 allele, they should submit the versions that they sequenced that appear to be non-functional for reference and include a figure showing where the mutations are in the gene.

Line 76: Should add the name of the resistance gene at the end of the sentence, after Ae. tauschii.

Line 78: Change wording to "the devastating disease, wheat stripe rust."

They have combined the first two paragraphs of the results section, these need to be split up again, probably at line 92.

Is the revelation of CIae9 possibly being PI511383 necessary for this story? Removing it would make these sections streamlined. If kept, this should be mentioned at the beginning of the results and then only one of them should be mentioned for the rest of the paper unless there are any other differences found.

Furthermore, if CIae9 = PI511383, and PI486274 ~= PI560536, then the two populations, popA and popB are almost exactly the same. They even removed the SNPs that were polymorphic between the two susceptible lines from the analysis. This makes the story a lot more confusing and could be condensed significantly!

Lastly, history needs to be provided on how CIae9, PI511383, PI486274 and PI560536 were generated. Does it make sense that CIae9 and PI511383 are the same? If not, could there have been a mix-up during planting? It happens.

Reviewer #3 (Remarks to the Author):

The authors present a revised manuscript addressing my comments on the original version of the submission. The data presentation in Figure 1d and e is much improved and clearer, I also appreciate the new Figure S9a. The authors have also added qRT-PCR data.

The manuscript still suffers from unclear writing and some problems with the logic of the presentation. I list a few of these aspects, but the authors must go through the entire manuscript to improve clarity.

Abstract : each is associated with…. : this needs to be rephrased and clarified.
Table 1 describes the genetic material used in the study. However, there is a column «YrAS2388R b», b means + positive for YrAS2388R by PCR. Is this a diagnostic marker developed in the work here ? It is disturbing to see a table using data from later in the manuscript early in the manuscript without really explaining what was done. This needs clarification and better presentation.
Results, l83 : it is referred to (explained below). Please explain and give the necessary information where it is needed and referred to.
Similar problem on l92.
Also l92: It is not clear why the Yr28 lines are presented in the text. The whole sub-chapter needs more clarity and logical flow.
L93 : .. the same locus… Same gene, or same locus ?
L110 : .. That they are from the same population… I interpret the data that they are the same genotype. Please clarify.
L171 : Was this verified with a specific, diagnostic PCR marker ? Do you have such a marker, where is it described ?

L220 : the duplicated 3’ UTRs was mostly identified by genome sequence analysis, and not only RNA-seq and RT-PCR.
L227 : Major isoforms : how is « major » defined ?
Figure 2b: the quality of the micrographs is not sufficient. The same is true for Figure S5.
Figure S2 : the authors should orient themselves on other papers how such a graphic could look like. For example, genes of the same class could have the same color. Is this a schematic representation ? Why not show a real comparative analysis, drawn to scale, and indicating bars with Kb and bp numbers to allow the reader to understand the region ?
I am still missing a clear annotation of the identified NLR. How many LRRs are present based on manual annotation?

Language issues :
L102 : were previously showed
L122 : To assure that we cloned the correct region… The region was not cloned here, you defined a region and want to verify that it is correctly identified.
L298: homologues… shares…

REVIEWERS'S COMMENTS IN GREEN

OUR RESPONSES IN BLUE

Reviewer #1 (Remarks to the Author):

I-1. The manuscript has been substantially revised, and somewhat simplified making the story easier to follow for the readers. It also appears that the authors have answered and/or reasonably well rebutted most of my comments. Nevertheless, some parts of the manuscript are still quite difficult to follow, and in my opinion **the Abstract requires further major rewrite/improvement**, spelling out novelty and importance of this study. **From the Abstract alone it is not clear at all why this work should be published in such a high-ranking journal as Nature Communications? What makes this R gene so special? What key discoveries were made in this study making it stand out from other map-based gene isolation studies? Is this the fact that the cloned gene is a typical NLR, but instead of controlling gene-for-gene resistance shown for many other cloned R genes, YrAS2388 controls broad spectrum resistance? Or is it all about alternative splicing?**

Response：Thank you for the comments. We modified the abstract somewhat. In the current abstract, the following results are novel.

"*YrAS2388R* has duplicated 3' untranslated regions and is characterized by alternative splicing."

"Transgenic *YrAS2388R* provides resistance to eleven *Pst* races in common wheat and one race of *P. striiformis* f. sp. *hordei* in barley."

"The *YrAS2388R* allele occurs only in *Ae. tauschii* and the *Ae. tauschii*-based synthetic wheat; it is absent in 100% (n=461) of common wheat lines tested."

I-2. The resistance gene cloned should be referred to as a typical NLR (or NB-LRR with an N-terminal CC-domain). Referring to the N -terminal feature of the encoded R protein as a "four helix bundle" doesn't make it any more special. In fact, the N-terminal feature I believe should still be referred to as a coiled-coil (or CC) domain. This is true that CC domains of some NLRs display a four-helical fold in a solution. This is yet to be formally shown for YrAS2388.

Response：In the abstract, the text now states that **"*YrAS2388R* encodes a classic nucleotide oligomerization domain-like receptor (NLR)**."

We call it a "four helix bundle" because of the actual structure of the Rx protein (JBC 288: 35868-35876) and the predicted structure of YrAS2388R. While we prefer to use "four helix bundle", we understand that either a "coiled-coil" or a "four helix bundle" does not change the nature of this classic NLR protein or make the gene more special. To avoid confusion, we deleted "with a N-terminal four-helix bundle (4HB which was previously classified as a coiled-coil)" from the abstract.

I-3. When referring to various alleles of YrAS2388 please use "resistance gene/allele" rather than "resistant gene/allele" throughout the manuscript.

Response：Corrected.

I-4. It would help the reader tremendously to more clearly indicate that alleles of YrAS2388 are present in Ae. tauschii as well as in T. aestivum, and in both resistant and susceptible genotypes. This is important. I'd suggest preparing a new figure showing a comparison of susceptibility and resistance versions of this gene, and the corresponding transcripts. Another useful figure I would like to see is the one showing an alignment of protein sequences encoded by susceptibility and resistance versions of YrAS2388 (this would include all "truncated" NLR versions potentially expressed from TV1 - TV4) and indicating the recognised protein domains and key motifs. How many leucine-rich repeats are present in the "full-length" YrAS2388?

Response: Thanks.

To differentiate the *Pst*-resistant versus *Pst*-susceptible alleles, we added the following statement. "Genome sequence analysis, RNA-seq and RT-PCR further revealed that the *NLR*$_{4DS-1}$ gene in PI511383 contains a 2,668-bp insertion, which resulted in duplicated 3' untranslated regions (3'UTR1 and 3'UTR2) and five transcript variants (Fig. D1d-e[39]). However, the *NLR*$_{4DS-1}$ gene in AL8/78 lacks the 2,668-bp insertion and has only one transcript product." Differences in protein and cDNA are included the supplementary Data 3. Considering that other reviewers asked us to condense the manuscript, we did not to add more figures to the current manuscript, although we are certainly willing to do so.

The issue of the *T. aestivum* susceptible alleles is more complicated. As we state that "The *Pst*-susceptible *NLR*$_{4DS-1}$ homologues in CS only share 86%-94% identity with the *Pst*-resistant *NLR*$_{4DS-1}$ in PI511383 at the cDNA level." Given that orthologous genes between genomes (A vs. B, A vs. D, and B vs. D) are about 95% identical at the cDNA level, it is difficult to claim a *Pst*-susceptible allele may or may not be allelic.

There might be 12 or more LRR repeats but we are not sure of the exact number. We rephrased the statement the text as "*NLR*$_{4DS-1}$ has a classic four-helix bundle (4HB, which was previously classified as a coiled-coil), a NB domain, and a LRR domain with 12 or more leucine-rich repeats."

LRSLILTGNLKSQPGDSLTIFPS
LRLLHIESANIAALVESMYQLKH
LRYLALKRTDMCRLPENIHEMKF
LQHISLEGCESFMKLPDSIIKLQG
LRYLDMGDTRVSSIPRGFRALTN
LTSLFGFPAYIDGDWCSLEELGSLSQ
LNELSLQSLENVSSALLAEKARVNAKKQ
LTVLALKCGGRVGHGLVQGEVSESKEEEQIIEAVFDV
LCPQPCIEHIRIERYFGRRLPGWMASTAMVPLES
LKILCLEHLPCCTQLPDGLCR
LPYLEWIKVMNAPVIKCIGPEF
VQQYNQLHRPSSQLAATFPK
LQMLEFHGMEEWEEWVWETEVKAMPL
LEELRITSCRLSRMPPGLMSHAMA
LKKLTIWSVQCLHSLENFVS
VVELELGNIPELAMISN

LPKLQKLTIECCPKLEMLQEMAA
LRRLELTIFNSENQLPVYLQTVKPSNLLLTCN

I-5. I can't fully agree with defining the 833-bp intron as "cryptic". This intron is flanked by canonical GT-AG splice sites in YrAS2388, and it is correctly spliced in TV2. This intron is simply "retained" in TV1. This intron also must have an alternative 5' splice site (what it is? – please specify), which allows alternative splicing as in TV3. In addition, I guess that TV3 and TV4 resulted from exon skipping events. I feel this needs to be clearly explained in the manuscript text.

Response：We agree that the "cryptic intron" has canonical GT-AG splice sites in the *Pst*-resistant NLR_{4DS-1}, which indeed acts as alternative splice sites for this cryptic intron.

TV1 is the most complete protein for both *Pst*-resistant and *Pst*-susceptible alleles. The defined "cryptic intron" involves NB and LRR domains. Here, we consider TV1 as an ancestral and complete transcript of the gene. The skipping of a region in the Exon 5, namely the "cryptic intron", resulted TV2.

We agree that the alternative splicing of a region between the same 5UTR and 3UTR involves exon skipping or intron retaining, for example TV1 vs. TV2, TV3 vs. TV4. However, alternative splicing between regions with different 3UTRs, it is not simply caused by exon skipping events, for example TV1 vs. TV3, TV2 vs. TV4.

The following change was made in the text: "The less abundant isoform TV2 might result from **either a partial exon skipping from TV1 or the retention of an 833-bp cryptic intron in exon 5**, which disrupts the NB and LRR domains. TV3 is also a less abundant isoform and is structurally similar to TV4, **but retains the first 244 bp in the 833-bp intron**, which only disrupts the LRR domain."

I-6. Now, the authors state (see L227-236) that Pst susceptible wheats AS87 and AL87/8 express only TV1 (an isoform that retains a 833-bp intron) and no fully spliced isoforms such as TV2. I therefore am puzzled as to why the complementation study was done using TV1? Wouldn't it be more logical to expect TV2 and/or TV3 and/or TV4 transcripts, that are not expressed in susceptible wheat genotypes, to play important role in Pst resistance?

Response：The complementation was done using the genomic allele, which expresses TV1 to TV4. Our model for gene activity includes all four transcripts.

Reviewer #2 (Remarks to the Author):
II-1. Though they fixed most of our issues, it appears our concern that the manuscript was long-winded and in need of being cut down was ignored. Not only did the authors add 70 lines, but they reduced the line spacing to make it seem like they had removed seven pages instead.
Response: We apologize, although we note that most of the reviewers' comments requested more information and not less. In response to this request, we have revised the manuscript as indicated here.

II-2. It is described that there are DNA polymorphisms between YrAS2388-containing lines and non-containing lines. What are these polymorphisms? What do they suggest about the function of YrAS2388? This is interesting information that is left out. In addition to the GenBank accession for the functional YrAS2388 allele, they should submit the versions that they sequenced that appear to be non-functional for reference and include a figure showing where the mutations are in the gene.

Response: Thank you. We have addressed this question in the first round revision. The related information can be found in the Supplementary Data 3.

We prepared a haplotype map of the NLR_{4DS-1} gene in nine selected genotypes. Crucial polymorphisms are shown in the map (Supplementary Data 3). The revised narrative is: "However, there was substantial DNA and protein variation between the *Pst*-resistant (PI511383 and PI511384) and *Pst*-susceptible (AS87 and AL8/78) genotypes that express NLR_{4DS-1} (Supplementary Data 3); in the NLR_{4DS-1} gene, PI511383 and AL8/78 are only 94% and 91% identical in cDNA and protein, respectively."

As suggested, we submitted susceptible alleles of the NLR_{4DS-1} gene to GenBank and revised the text as: "GenBank accessions include MK288012 for the fosmid PC1104 (=F2-1), MK288013 for the *YrAS2388R* contig, **MK736661-MK736663 for the *YrAS2388R* gene in PI511383, CIae9 and AS2388 and MK736664-MK736666 for the *YrAS2388S* gene in PI560536, PI486274 and AS87, respectively**." Variations between *Pst*-resistant and *Pst*-susceptible alleles are illustrated in the haplotype map (Supplementary Data 3).

II-3. Line 76: Should add the name of the resistance gene at the end of the sentence, after Ae. tauschii.

Response：Done

II-4. Line 78: Change wording to "the devastating disease, wheat stripe rust."

Response：Done

II-5. They have combined the first two paragraphs of the results section, these need to be split up again, probably at line 92.

Response：Done. They are misplaced in the same paragraph.

II-6. Is the revelation of CIae9 possibly being PI511383 necessary for this story? Removing it would make these sections streamlined. If kept, this should be mentioned at the beginning of the results and then

only one of them should be mentioned for the rest of the paper unless there are any other differences found.

Response: Thanks for your suggestion. The revelation that CIae9 is possibly PI511383 is not necessary. We removed the related content to condense the manuscript, but kept both CIae9 and PI511383 in the manuscript.

II-7. Furthermore, if CIae9 = PI511383, and PI486274 ~= PI560536, then the two populations, popA and popB are almost exactly the same. They even removed the SNPs that were polymorphic between the two susceptible lines from the analysis. This makes the story a lot more confusing and could be condensed significantly!

Response: Thank you. We understand your point. Because BSA was performed on only 17 *Pst*-susceptible F_2 plants, including 10 from popA and 7 from popB. We could include the polymorphic SNPs between the two susceptible lines for the BSA analysis, however the current population sizes were too small, i.e., only 10 (popA) or 7 (popB), which is too small for BSA. After removing polymorphic SNPs between the two susceptible lines, we have 3,276 SNP loci that were adequate for the BSA analysis.

II-8. Lastly, history needs to be provided on how CIae9, PI511383, PI486274 and PI560536 were generated. Does it make sense that CIae9 and PI511383 are the same? If not, could there have been a mix-up during planting? It happens.

Response：Thanks. We eliminated the relationship between CIae9 and PI511383. However, we added the source of these germplasm as “All *Ae. tauschii* accessions except for ‘AL8/78’ and ‘AS87’ were provided by Dr H.E. Bockelman at the USDA-ARS, Aberdeen, ID, USA.”

Reviewer #3 (Remarks to the Author):

III-1. The authors present a revised manuscript addressing my comments on the original version of the submission. The data presentation in Figure 1d and e is much improved and clearer, I also appreciate the new Figure S9a. The authors have also added qRT-PCR data. The manuscript still suffers from unclear writing and some problems with the logic of the presentation. I list a few of these aspects, but the authors must go through the entire manuscript to improve clarity.

Response：Thanks. We have tried to clarify the story and to present it in a more logical manner.

III-2. Abstract : each is associated with…. : this needs to be rephrased and clarified.

Response：It is rephrased as “The *Pst*-resistant allele *YrAS2388R* has duplicated 3’ untranslated regions and is characterized by alternative splicing in the nucleotide-binding domain.”

III-3. Table 1 describes the genetic material used in the study. However, there is a column «YrAS2388R b», b means + positive for YrAS2388R by PCR. Is this a diagnostic marker developed in the work here ? It is disturbing to see a table using data from later in the manuscript early in the manuscript without really explaining what was done. This needs clarification and better presentation.

Response: We rephrased the table note to "[b]'+'=positive for the *YrAS2388R* (or *Yr28*) locus; '-'=negative for the *YrAS2388R* (or *Yr28*) locus."

III-4. Results, l83 : it is referred to (explained below). Please explain and give the necessary information where it is needed and referred to.

Response: This part is deleted in the current version.

III-5. Similar problem on l92. Also l92: It is not clear why the Yr28 lines are presented in the text. The whole sub-chapter needs more clarity and logical flow.

Response: Thanks. We rephrased as "Previously, we hypothesized that *YrAS2388* and *Yr28* are **the same gene** [26]. AvSYr28NIL and AvS are near-isogenic lines (NILs) for the *Yr28* locus."

III-6. L93 : .. the same locus… Same gene, or same locus ?

Response: We have now changed to "**the same gene"** (as in our response to III-5).

III-7. L110 : .. That they are from the same population… I interpret the data that they are the same genotype. Please clarify.

Response: This part was deleted from the text in response to II-6.

III-8. L171 : Was this verified with a specific, diagnostic PCR marker ? Do you have such a marker, where is it described ?

Response: Based on their pedigree, both SW3 and Syn-SAU-93 should have the *YrAS2388R* gene. Both genotypes are positive for three diagnostic PCR markers (PM, GM and TM); the *YrAS2388R* gene in SW3 is shown in Figure SD11. In an attempt to make the presentation as rational as possible, we did not include the marker information here.

III-9. L220 : the duplicated 3' UTRs was mostly identified by genome sequence analysis, and not only RNA-seq and RT-PCR.

Response: Thanks. We revised as "Genome sequence analysis, RNA-seq and RT-PCR further revealed that the NLR_{4DS-1} gene in PIPI511383 contains ……"

III-10. L227 : Major isoforms : how is « major » defined ?

Response: We revised "major" to "**four identified**"

III-11. Figure 2b: the quality of the micrographs is not sufficient. The same is true for Figure S5.

Response: We apologize, but we believe that the images are interpretable, especially if the viewer enlarges the image. We note that the orange-brown uredinial pustules of rust spores on a susceptible plant and the tannish-yellow necrotic lesions on a resistant plant are not easy to capture.

III-12. Figure S2 : the authors should orient themselves on other papers how such a graphic could look like. For example, genes of the same class could have the same color. Is this a schematic representation ? Why not show a real comparative analysis, drawn to scale, and indicating bars with Kb and bp numbers to allow the reader to understand the region ?

Response: Thanks. We now have used the same color for the same type of gene. A schematic representation and a scale bar were prepared for Chinese Spring, which has a complete genome sequence. The exact location (in bp) of two genes was added to the chromosome 4D in Chinese Spring.

III-13. I am still missing a clear annotation of the identified NLR. How many LRRs are present based on manual annotation?

Response: It appears that there might be 12 or more LRR repeats, but we are not sure of the correct number. We rephrased the statement in the text as "*NLR*$_{4DS-1}$ has a classic four-helix bundle (4HB, which was previously classified as a coiled-coil), a NB domain, and a LRR domain with 12 or more leucine-rich repeats."

LRSLILTGNLKSQPGDSLTIFPS
LRLLHIESANIAALVESMYQLKH
LRYLALKRTDMCRLPENIHEMKF
LQHISLEGCESFMKLPDSIIKLQG
LRYLDMGDTRVSSIPRGFRALTN
LTSLFGFPAYIDGDWCSLEELGSLSQ
LNELSLQSLENVSSALLAEKARVNAKKQ
LTVLALKCGGRVGHGLVQGEVSESKEEEQIIEAVFDV
LCPQPCIEHIRIERYFGRRLPGWMASTAMVPLES
LKILCLEHLPCCTQLPDGLCR
LPYLEWIKVMNAPVIKCIGPEF
VQQYNQLHRPSSQLAATFPK
LQMLEFHGMEEWEEWVWETEVKAMPL
LEELRITSCRLSRMPPGLMSHAMA
LKKLTIWSVQCLHSLENFVS
VVELELGNIPELAMISN

LPKLQKLTIECCPKLEMLQEMAA
LRRLELTIFNSENQLPVYLQTVKPSNLLLTCN

III-14. Language issues: L102 : were previously showed

Response : Corrected.

III-15. L122 : To assure that we cloned the correct region… The region was not cloned here, you defined a region and want to verify that it is correctly identified.

Response : Corrected.

III-1. L298: homologues… shares…

Response : Corrected.

REVIEWERS' COMMENTS:

Reviewer #2 (Remarks to the Author):

Zhang et al., have addressed all of our issues, we especially appreciate the inclusion of Supplemental Data 3 and the submission of the sequences for the susceptible alleles of YrAS2388. With very minor amendments, we advise acceptance.

The abstract could do with a little more refinement. Such as changing the following section:

From:
"The wheat progenitor, Aegilops tauschii, is the source of the allele YrAS2388R for resistance to Pst at the YrAS2388 locus on chromosome 4DS. We reveal that YrAS2388 encodes a classic nucleotide oligomerization domain-like receptor (NLR). The Pst-resistant allele YrAS2388R has duplicated 3' untranslated regions and is characterized by alternative splicing in the nucleotide-binding domain."

To:
"The wheat progenitor, Aegilops tauschii, is a source of resistance to Pst conferred by YrAS2388. We show that YrAS2388 encodes a classic nucleotide oligomerization domain-like receptor (NLR) on chromosome 4DS. The Pst-resistant allele, YrAS2388R, has a duplicated 3' untranslated regions and is characterized by alternative splicing in the nucleotide-binding domain."

This rewrite of the abstract is a bit easier to follow.

Reviewer #3 (Remarks to the Author):

The authors have addressed my concerns on earlier versions of the manuscript.

Reviewer #4 (Remarks to the Author):

I satisfy with the responses from the authors to the questions raised from reviewer 1.

To further improve readability of abstract, I suggest to revise part of content as:
... Aegilops tauschii, one of the wheat progenitor, carries the YrAS2388 locus for resistance to Pst on chromosome arm 4DS. We reveal that YrAS2388 encodes a typical nucleotide oligomerization domain-like receptor (NLR), but the Pst-resistance allele (YrAS2388R) has duplicated 3' untranslated regions and is characterized by alternative splicing in the nucleotide-binding domain. Mutation of the YrAS2388R allele disrupts its resistance to Pst in synthetic hexaploid wheat; transgenic plants with YrAS2388R showed resistance to 11 Pst races in common wheat and one Pst race in barley. The YrAS2388R allele occurs only in Ae. tauschii and the Ae. Tauschii-derived synthetic wheat; ...

REVIEWERS' COMMENTS IN GREEN

OUR RESPONSES IN BLUE

Reviewer #3 (Remarks to the Author):

The authors have addressed my concerns on earlier versions of the manuscript.

Response: Thank you.

Reviewer #2 (Remarks to the Author):

Zhang et al., have addressed all of our issues, we especially appreciate the inclusion of Supplemental Data 3 and the submission of the sequences for the susceptible alleles of YrAS2388. With very minor amendments, we advise acceptance.

The abstract could do with a little more refinement. Such as changing the following section:

From: "The wheat progenitor, Aegilops tauschii, is the source of the allele YrAS2388R for resistance to Pst at the YrAS2388 locus on chromosome 4DS. We reveal that YrAS2388 encodes a classic nucleotide oligomerization domain-like receptor (NLR). The Pst-resistant allele YrAS2388R has duplicated 3' untranslated regions and is characterized by alternative splicing in the nucleotide-binding domain."

To: "The wheat progenitor, Aegilops tauschii, is a source of resistance to Pst conferred by YrAS2388. We show that YrAS2388 encodes a classic nucleotide oligomerization domain-like receptor (NLR) on chromosome 4DS. The Pst-resistant allele, YrAS2388R, has a duplicated 3' untranslated regions and is characterized by alternative splicing in the nucleotide-binding domain."

This rewrite of the abstract is a bit easier to follow.

Response: Thank you. We considered your comment and the one from the #4 reviewer and revised the abstract accordingly. The revision is in our response to the #4 reviewer.

Reviewer #4 (Remarks to the Author):

I satisfy with the responses from the authors to the questions raised from reviewer 1.

To further improve readability of abstract, I suggest to revise part of content as:

... Aegilops tauschii, one of the wheat progenitor, carries the YrAS2388 locus for resistance to Pst on chromosome arm 4DS. We reveal that YrAS2388 encodes a typical nucleotide oligomerization domain-like receptor (NLR), but the Pst-resistance allele (YrAS2388R) has duplicated 3' untranslated regions and is characterized by alternative splicing in the nucleotide-binding domain. Mutation of the YrAS2388R allele disrupts its resistance to Pst in synthetic hexaploid wheat; transgenic plants with YrAS2388R showed resistance to 11 Pst races in common wheat and one Pst race in barley. The YrAS2388R allele occurs only in Ae. tauschii and the Ae. Tauschii-derived synthetic wheat; ...

Response: Thank you. We revised the abstract by considering both #2 and #4 reviewers' comments.

Here is the revised abstract: "*Aegilops tauschii*, one of the wheat progenitors, carries the *YrAS2388* locus for resistance to *Pst* on chromosome 4DS. We reveal that *YrAS2388* encodes a typical nucleotide oligomerization domain-like receptor (NLR). The *Pst*-resistant allele *YrAS2388R* has duplicated 3' untranslated regions and is characterized by alternative splicing in the nucleotide-binding domain. Mutation of the *YrAS2388R* allele disrupts its resistance to *Pst* in synthetic hexaploid wheat; transgenic plants with *YrAS2388R* show resistance to eleven *Pst* races in common wheat and one race of *P. striiformis* f. sp. *hordei* in barley. The *YrAS2388R* allele occurs only in *Ae. tauschii* and the *Ae. tauschii*-derived synthetic wheat; it is absent in 100% (n=461) of common wheat lines tested."
